# Abnormal thermally-stimulated dynamic organic phosphorescence

**DOI:** 10.1038/s41467-024-45811-0

**Published:** 2024-03-08

**Authors:** He Wang, Huili Ma, Nan Gan, Kai Qin, Zhicheng Song, Anqi Lv, Kai Wang, Wenpeng Ye, Xiaokang Yao, Chifeng Zhou, Xiao Wang, Zixing Zhou, Shilin Yang, Lirong Yang, Cuimei Bo, Huifang Shi, Fengwei Huo, Gongqiang Li, Wei Huang, Zhongfu An

**Affiliations:** 1 Key Laboratory of Flexible Electronics (KLoFE) & Institute of Advanced Materials (IAM), Nanjing Tech University, Nanjing, China; 2 Frontiers Science Center for Flexible Electronics (FSCFE), MIIT Key Laboratory of Flexible Electronics (KLoFE), Northwestern Polytechnical University, Xi’an, 710072 China; 3 College of Electrical Engineering and Control Science, Nanjing Tech University, Nanjing, China; 4 The Institute of Flexible Electronics (IFE, Future Technologies), Xiamen University, Xiamen, 361005 Fujian China; 5 Key Laboratory for Organic Electronics and Information Displays & Institute of Advanced Materials (IAM), Nanjing University of Posts & Telecommunications, Nanjing, China

**Keywords:** Self-assembly, Organic molecules in materials science, Organic molecules in materials science

## Abstract

Dynamic luminescence behavior by external stimuli, such as light, thermal field, electricity, mechanical force, etc., endows the materials with great promise in optoelectronic applications. Upon thermal stimulus, the emission is inevitably quenched due to intensive non-radiative transition, especially for phosphorescence at high temperature. Herein, we report an abnormal thermally-stimulated phosphorescence behavior in a series of organic phosphors. As temperature changes from 198 to 343 K, the phosphorescence at around 479 nm gradually enhances for the model phosphor, of which the phosphorescent colors are tuned from yellow to cyan-blue. Furthermore, we demonstrate the potential applications of such dynamic emission for smart dyes and colorful afterglow displays. Our results would initiate the exploration of dynamic high-temperature phosphorescence for applications in smart optoelectronics. This finding not only contributes to an in-depth understanding of the thermally-stimulated phosphorescence, but also paves the way toward the development of smart materials for applications in optoelectronics.

## Abstract (web summary)

In thermally stimulated phosphorescent materials a thermal stimulus inevitably quenches the emission due to intensive non radiative transition. Here, the authors report an abnormal thermally stimulated phosphorescence behavior in organic phosphors and show enhancement of phosphorescence and change of the emission color upon temperature increase.

## Introduction

Thermal stimulation plays a significant role in manipulating photoelectric properties of materials for various applications. For instance, heat can trigger the death of cancer cells for photothermal therapy^1,2^. It can also control the conductivity of thermoelectric materials^3,4^ and change the transparency of thermochromic smart windows^5,6^. Besides, in the process of luminescence, thermal field can regulate excited-state energetics, lifetimes, and spin characteristics for applications in sensing, photocatalytic reactions, and optoelectronic devices^7–12^. One of the best known is thermally activated delayed fluorescence (TADF)^13,14^, in which reverse intersystem crossing (RISC) of excitons can be thermally promoted from the lowest excited triplet states (T_1_) to the lowest singlet state (S_1_). It is favorable for the efficiency enhancement in organic light-emitting diodes (OLEDs) by converting the electronically excited triplet excitons to the singlet ones. In addition, dynamic luminescence behaviors under thermal stimulation have also been reported in some thermally activated delayed fluorescent/phosphorescent molecules, which endow materials with fascinating functionalities^15,16^. Although some progress has been made, great effort on thermally-stimulated luminescent materials and related mechanisms is still devoted.

**Fig. 1:**
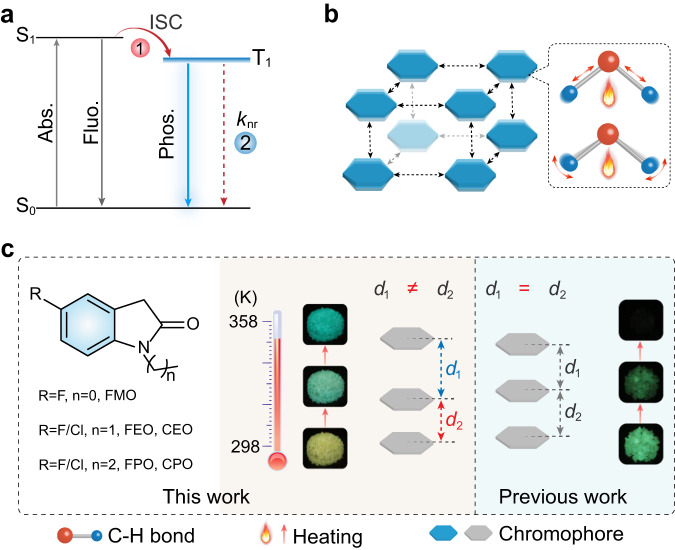
A schematic representation of thermally-stimulated dynamic organic phosphorescence. **a** Energy transfer processes for phosphorescence in organic phosphors. Promoting the intersystem crossing (ISC) process (step 1) and reducing the rate of nonradiative transition (step 2) are key points for boosting phosphorescence. **b** Organic molecular motion model under thermal stimulus. **c** Presentation for influence of molecular stacking on phosphorescent behavior under thermal stimulus in previous and this works. Notably, intermolecular stacking distance and interactions along the direction of π-π stacking are usually even. The phosphorescence fades away as temperature increasing in previous work. On the contrary, there exists uneven intermolecular stacking in this work. Under thermal stimulus, the excited molecular conformations undergo deformation, thus affording the abnormally dynamic phosphorescence.

Phosphorescence, compared to fluorescence, bearing long-lived emission lifetimes, large Stokes shifts, and rich excited states, has been extensively used in photocatalysis, bio-electronics, and optoelectronic devices^17–22^. So far, the phosphorescence is concentrated on metal-containing complexes^23,24^, on account of the heavy-atom effect on the spin-orbit coupling (SOC) enhancement. Purely organic phosphors are attractive alternatives to the organometallic molecules due to their environmentally friendly nature, structural adjustability, and low cost. Early reported organic phosphorescence is mainly achieved at low temperature (77 K)^25–27^. Because low temperature can restrict the molecular motion, thus inhibiting fast nonradiative decay of the excitons from T_1_ to the ground state (S_0_) (Fig. 1a). Recently, enormous efforts have been devoted to boosting organic phosphorescence at room temperature (~298 K) by a series of strategies, like rigid crystalline inducement^28–34^, metal-organic frameworks construction^35–37^ and host-guest doping^38–41^, etc^42–48^. Notably, optical multiplexing capability endows emissive materials with functional applications by building the dimension of temperature. There also exist some researches on temperature dependent phosphorescence between 77 K to room temperature^49^, rendering phosphorescence behavior dynamic. To date, the reports on purely organic phosphorescence are rare at higher temperature (Supplementary Fig. 21). Since thermal causes dramatic motions of the molecules as temperature increasing (Fig. 1b), thus leading to phosphorescence quenching^50^. The issue of phosphorescence thermal-quenching in organic matters remains a daunting challenge.

## Results

### Synthesis and characterizations

Coincidentally, we found a series of indolinone derivatives that exhibit abnormal thermally-stimulated dynamic phosphorescence at high temperature. As the temperature increases over room temperature, the indolinone materials display ratio-metric phosphorescent behavior along with dynamic circulation of afterglow color between yellow and indigo-blue (Supplementary Movie 1). It contrasts with the traditional phosphorescence materials, of which the phosphorescence fades away with temperature increasing (Fig. 1c and Supplementary Fig. 22). The indole oxide derivatives, 1-methyl-5-fluoro-1H-indole-2-one (FMO), 1-ethyl-5-fluoro-1H-indole-2-one (FEO), propyl-5-fluoro-1H-indole-2-one (FPO), 1-ethyl-5-fluoro-1H-indole-2-one (CEO) and propyl-5-fluoro-1H-indole-2-one (CPO) were synthesized by two-steps reaction (Supplementary Fig. 1), which showed transparent crystals by repeated recrystallization (Supplementary Fig. 2). The chemical structures of target molecules were thoroughly verified by ^1^H and ^13^C NMR spectroscopies, elemental analysis as well as X-ray single crystal analysis. Furthermore, high-performance liquid chromatography (HPLC) was also performed to rule out possible contamination of the emissive impurity in the target compounds (Supplementary Figs. 3–19). No impurity was found. Additionally, differential scanning calorimetry testing showed that, except for crystallization and melting, there was no other phase transformation for these phosphors (Supplementary Fig. 20).

### Photophysical properties of the model phosphor

**Fig. 2:**
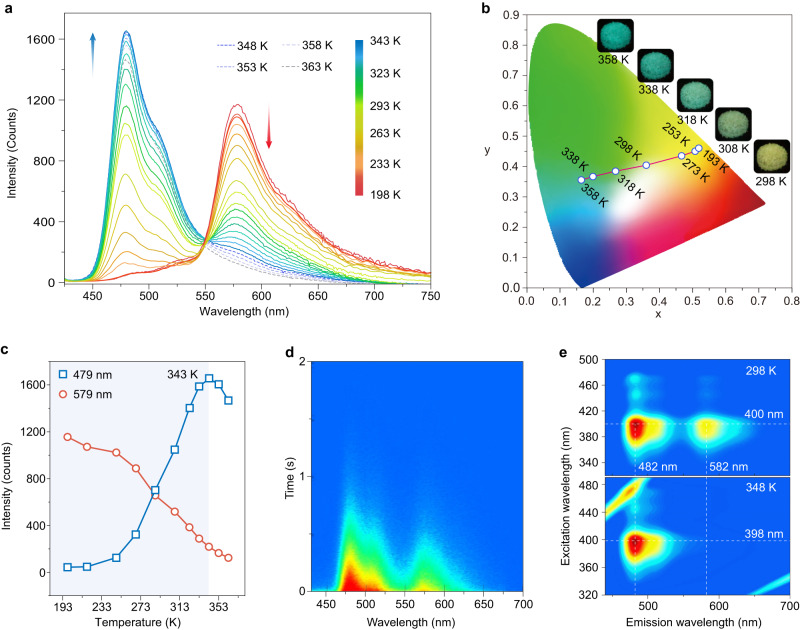
Investigation of the photoluminescence for crystalline FPO powder. **a** Temperature dependent phosphorescence spectra excited by 400 nm. Note that the phosphorescence was collected with a delay time of 8 ms. **b** A CIE chromaticity coordinate diagram of the phosphorescence at different temperature. It is recorded by regulating temperature from 193 to 358 K. The inset images show the long-lived luminescence for FPO phosphors at 298, 308, 318, 338 and 358 K, respectively. **c** A plot of phosphorescence intensity monitoring the emission bands at 479 and 579 nm as a function of temperature. **d** Time-resolved emission spectra excited by 400 nm at room temperature. **e** Excitation–phosphorescence emission mapping at 298 and 348 K.

Firstly, the FPO molecule was selected as a model for photophysical property investigation in dilute solution and solid state. The FPO molecules exhibited absorption bands at 251 and 295 nm, as well as an emission band at 322 nm in dichloromethane solution (1 × 10^−5^ M) (Supplementary Fig. 23). In solid state, the absorption spectrum of FPO molecules had a main band at around 300 nm with a broad shoulder at around 400 nm (Supplementary Fig. 24). The FPO phosphor exhibited phosphorescence efficiency of 5.7% in solid state under ambient conditions. As shown in Fig. 2a, the spectrum of FPO crystal with a delay time of 8 ms displays a main emission band at around 579 nm at 198 K. With the temperature increasing from 198 to 363 K, the emission bands at 579 nm gradually disappeared. Unexpected, a new emission band with the peak at 479 nm and a shoulder around 510 nm rise simultaneously. The lifetimes of emission bands at 479 and 579 nm are beyond 0.7 s (Supplementary Fig. 25), indicating its phosphorescent nature. Notably, there is no change in the profiles of the steady-state photoluminescence (PL) spectra but for emission intensity decreased with elevated temperature, which have emission bands at around 340 nm (Supplementary Fig. 26). The emission lifetime is only 0.44 ns at room temperature. Dynamic variation of phosphorescence color by thermal stimulus is further exhibited in the Commission International de l’Eclairage (CIE) coordinate diagram (Fig. 2b). The phosphorescence color changed from yellow to indigo-blue with good linearity of the CIE coordinates as the temperature gradually changed from 193 to 358 K.

The abnormal phosphorescence emission at 479 nm under thermal stimulus was more intuitively displayed in the plot diagram of corresponding phosphorescence intensities versus temperature (Fig. 2c). When the temperature is below 343 K, the phosphorescence intensity displays climb-out tendency as temperature increasing. It is different from conventional phosphorescent materials with thermal quenching characteristic, like the variation of the emission band at 579 nm. When the temperature is higher than 343 K, fierce molecular motion could result in the domination of adverse non-radiative transition, thus quenching the phosphorescence at 479 nm. From Fig. 2d, it was found that the profiles of the phosphorescence fixed with main peaks at 479 and 579 nm as time decay. It is worth noting that whatever at 298 and 348 K, the phosphorescence emission of the FPO phosphor is independent of excitation wavelength (Fig. 2e). In vacuum, oxygen or water environment, FPO crystal still exhibits dynamic and bright phosphorescence as temperature variation, demonstrating oxygen and moisture have no effect on dynamic phosphorescent behavior (Supplementary Figs. 27–29).

### Phosphorescence mechanism investigation

**Fig. 3:**
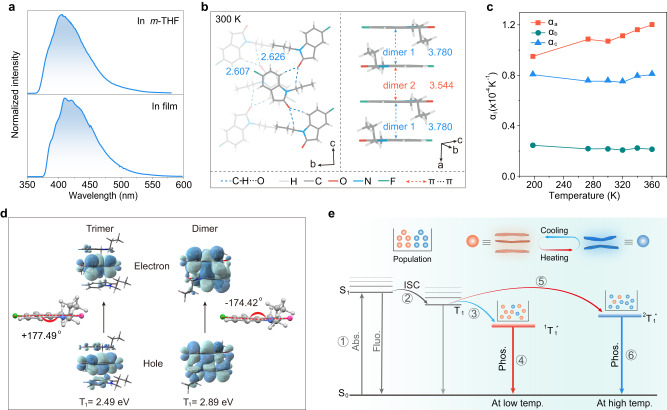
A plausible mechanism for abnormal thermally-stimulated dynamic phosphorescence. **a** Normalized phosphorescence spectra of FPO molecule in m-THF solution and PMMA film at 77 K. **b** Molecular arrangement of FPO molecules viewed along a axis (left) and π-π stacking (right) in crystal. **c** Thermal expansion coefficient of FPO crystal along the principal axes (**a**–**c**) at different temperature from 90 to 360 K. **d** Natural transition orbitals (NTOs) contributing to the lowest-energy triplet transitions of FPO trimer and dimer models in crystal. The inset structures show the molecular conformation in excited state of trimer (left) and dimer (right) models. **e** Proposed energy transfer processes for thermally-stimulated dynamic phosphorescence. Note that molecules at ground state (S_0_) reaches to S_1_ (step 1) after absorption of photons, and then transform to T_1_ through ISC (step 2). Under lower temperature, the excited molecules at T_1_ are further stabilized by the triplet excited state (^1^T_1_^*^) of trimer with a lower energy level (step 3), enabling long-lived yellow phosphorescence (step 4). As temperature increases, the excited molecule experiences large conformation adjustment in an environment of uneven intermolecular interactions, which was further stabilized for ^2^T_1_^*^ (step 5) of dimer. Finally, a bright cyan-blue phosphorescence was observed via radiative transition from ^2^T_1_^*^ (step 6).

To gain a deeper insight into the abnormal thermally-stimulated dynamic phosphorescence, a series of control experiments were conducted. In view of the large energy gap (~1.06 eV) between 340 and 479 nm, we also excluded the possibility of TADF for the emission around 479 nm, which is quite different from the classic TADF cases^14^. From time-resolved emission spectra, the short-lifetime fluorescence at 340 nm disappears, whereas long-lived emissions at 479 and 579 nm become dominant with fixed ratio as delayed time increases, further indicating the long-lived emission at 479 nm is not TADF (Supplementary Fig. 30). As shown in Fig. 3a, the FPO molecules exhibited broad blue emission band around 403 nm in a dilute solution of 2-methyltetrahydrofuran and polymethyl methacrylate-encapsulated film indicating the emission at 479 and 579 nm also did not stem from the isolated molecules in dispersed state. It is further proved by a comparison between the absorption spectrum of FPO phosphor in dilute solution and the excitation spectra of that in the crystalline state (Supplementary Fig. 31). There is almost no overlap. In addition, the possibility of new excited states caused by impurities was also excluded by the identical excitation spectra of phosphorescence emission at 488 nm at 198 K and 589 nm at 298 K for the FPO phosphor (Supplementary Fig. 32). Moreover, the similar lifetimes at low temperature also indicated phosphorescence characteristic of the emission at 579 and 750 nm are identical (Supplementary Fig. 33).

We further investigated the influence of aggregated states on the unique dynamic phosphorescence by single-crystal X-ray diffraction analysis. Each FPO molecule can form strong interaction of C-H···O with two adjacent molecules (Fig. 3b, Supplementary Fig. 34 and Supplementary Table 1), which can efficiently confine the fierce motions of FPO molecules and then reduce non-radiative transition of the triplet excitons for the phosphorescence generation. Between the neighboring molecules along π-π stacking (Fig. 3b), it exhibits face-to-face antiparallel arrangements with uneven molecular stacking (dimer 1 and dimer 2), and there are merely weak π-π interactions. We reasoned that such a unique molecular arrangement would enable the crystal thermal expanding along π-π stacking. As suspected, with temperature increases from 90 to 360 K, FPO crystal exhibited exceptionally large positive volumetric thermal expansion (Supplementary Fig. 35). According to the range of thermal expansion coefficients (in K^−1^), 9.5 × 10^−5^ < *α*_*a*_ < 1.2 × 10^−4^; 2.1 × 10^−5^ < *α*_*b*_ < 2.5 × 10^−5^ and 8.0 × 10^−5^ < *α*_*c*_ < 8.1 × 10^−5^, the direction of thermal expanding is mainly along crystallographic **a** axe which corresponds exactly π-π stacking direction (Fig. 3c, Supplementary Figs. 36 and 37). Therefore, we concluded that the variation of molecular microenvironment upon heating might play a vital role in manipulating the excited states of the molecules for unique phosphorescence properties.

To verify our hypothesis, theoretical calculations were carried out subsequently on the basis of the QM/MM model (Supplementary Fig. 38). According to simulating the T_1_ in different aggregated states, we found the calculated energy gap between dimeric and trimeric states agreed well with that of 0.44 eV between 479 and 579 nm in the experiment (Fig. 3d and Supplementary Figs. 39–41). Unexpectedly, we found the molecular conformation at the excited state experiences large deformation between the trimeric and dimeric states, in which the molecular bending direction changes from the opposite side of the propyl chain to the same direction. It causes the triplet energy level of molecules in dimer higher than that at the trimeric state (Fig. 3d). Taking the uneven intermolecular stacking in crystal, we speculated that the obvious molecular deformation might stem from the inherent characteristic of the FPO molecule and the variation of microenvironment within the crystal upon heating. From electrostatic potential (δ) analysis in Supplementary Fig. 42, it was found that negative δ (δ^−^) distributed on both ends of the FPO molecule and a small part of benzene units, while positive δ (δ^+^) localized on nitrogen heterocycle and alkyls. Considering face-to-face antiparallel arrangements of the FPO molecules in crystal, we reasoned that the molecular deformation toward the direction of propyl chain was ascribed to electrostatic repulsion at both ends of FPO molecule and electrostatic attraction at the middle region.

Taking the experimental and theoretical simulation, we proposed a plausible mechanism for dynamic phosphorescence stimulated upon thermal. Under lower temperature, the molecules at the ground state transfer to the excited molecular conformation through ISC after UV-light irradiation, which is like that in the trimer simulated by TD-DFT. Then the phosphorescence was produced by radiative transition of exited molecules in crystal. As temperature increases, the molecules show strenuous exercise owing to uneven interactions along **a** axe (Supplementary Fig. 43). With thermal assistance, the molecules overcome the energy barrier and then convert into the molecular conformation like that in dimer after photo-excitation (Supplementary Fig. 44 and Fig. 3e), thus boosting phosphorescence with high energy. Notably, the two populations related to the emissions at low and high temperatures are in thermally equilibrium but not kinetically linked to each other in their excited states based on the large energy gap (0.44 eV). Namely, the population ratio corresponding to the emissions at 479 and 579 nm changes with temperature, but no interconversion between the two populations occurs during their excited states. It was also proved by the superposition of both excitation spectra of the emissions at 479 and 579 nm at the same temperatures (Supplementary Fig. 31).

### Material expansion

**Fig. 4:**
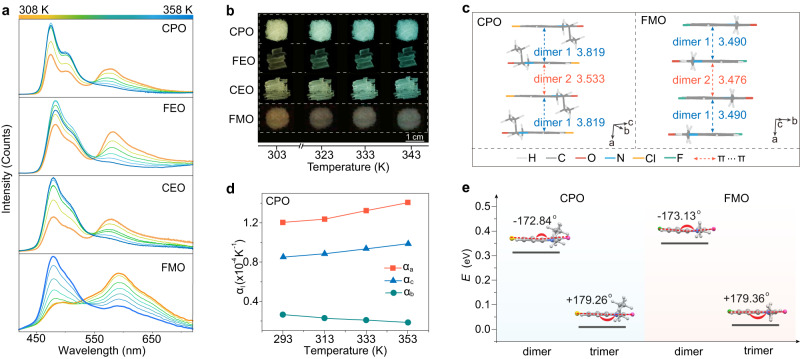
Phosphorescence properties and crystal structures of the control phosphors. **a** Temperature dependent phosphorescence spectra of the CPO, FEO CEO, and FMO phosphors excited by 400 nm. **b** Afterglow photographs of the crystalline CPO, FEO CEO, and FMO materials taken by varying temperature from 303 to 343 K. **c** Intermolecular π-π stacking in CPO and FMO crystals at room temperature. **d** Thermal expansion coefficient of the CPO crystal along the principal axes (**a**–**c**) at the temperature from 273 to 353 K. **e** Conformational energies estimated for the optimized structures of the CPO (left) and FMO (right) molecules in dimer and trimer state.

The universality of the hypothesis was also demonstrated by a series of molecules (FMO, FEO, CEO, and CPO). As shown in Fig. 4a and Supplementary Figs. 45–49, these molecules all manifested thermally-stimulated dynamic phosphorescence emission in the crystalline state. Like crystalline FPO material, the phosphorescence emission around higher energetic bands enhanced and lower ones declined as temperature increasing. Ultralong phosphorescence colors were also dynamically tuned upon heating (Fig. 4b and Supplementary Fig. 50). As anticipated, there also existed uneven molecular stacking and weak π-π interactions in these molecules (Fig. 4c, Supplementary Figs. 51–54 and Supplementary Tables 2–4), which resulted in thermal expanding mainly along π-π stacking direction (Fig. 4d and Supplementary Figs. 55–58). Moreover, the electrostatic potential distribution of these phosphors was similar to the FPO (Supplementary Fig. 59), offering possibilities for the deformation of excited state molecules and thus dynamic transition of phosphorescence (Supplementary Figs. 60–62). Based on the understanding of above results, we believe the materials with uneven π-π stacking of molecules would present more possibilities to obtain stimulus-responsive dynamic phosphorescence.

### Potential applications of the phosphors

**Fig. 5:**
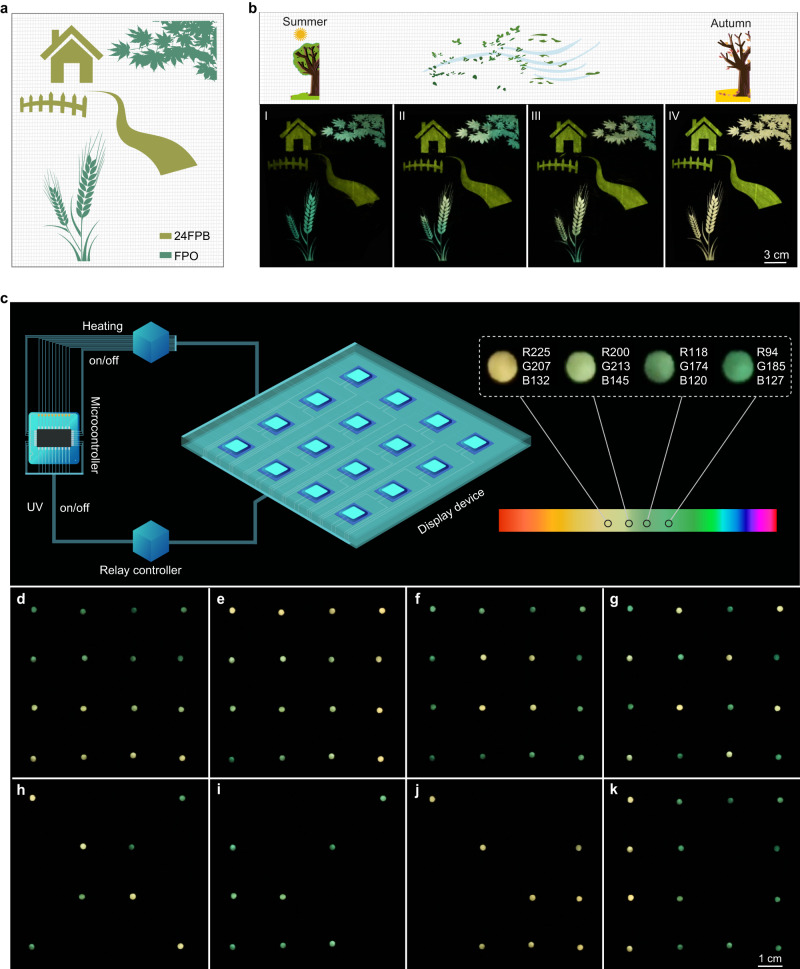
Demonstration of thermal-responsive phosphors for smart pigments and colorful afterglow display. **a** Schematic diagram for screen printing pattern of landscape. **b** Afterglow photographs of landscape pattern taken as temperature change from 323 K to room temperature. Note that the photographs from I to IV represents the seasonal change from summer to autumn. **c** A schematic of colorful afterglow display device (left) and afterglow photographs with specific color values through heating for different times (right). **d**–**k** Colorful afterglow patterns with integrated circuit by programmable regulation of the UV lamps and heating plate on-off, respectively.

According to the unique thermally-stimulated dynamic phosphorescence, we demonstrated their potential applications in smart luminescent dyes and colorful afterglow display devices. The PFO phosphor exhibits good reversibility and stability after multiple cycles of variable temperature (Supplementary Fig. 63). After grinding, it still displays dynamic phosphorescent behavior (Supplementary Fig. 64). As shown in Fig. 5a, the pattern of landscape was fabricated through a silk-screen printing technique with the grinding PFO powders and 2,4­difluorophenylboronic acid (24FPB) dyes as solid inks. The house and fence were printed with 24FPB phosphor, while maple leaves and wheat ears were printed using the FPO ink. With the temperature decreasing from 50 °C to room temperature that simulates the season changing from summer to autumn, the colors of maple leaves and wheat ears gradually become yellow, while that of the house and fence are invariable (Fig. 5b). Such temperature-sensitive luminescent dyes might dress the art of painting up to be charming and extraordinary splendid. Besides, more dynamically colorful paintings were also demonstrated (Supplementary Fig. 65). In addition, the ultralong phosphorescence changes upon heating might provide a sight for colorful afterglow displayer. Thereout, we first designed a prototype device as a pixel, mainly including heating plate, UV bead, sample layer, and mask (Supplementary Fig. 66), and then integrated them into a colorful afterglow displayer of 4*4 matrix along with a microcontroller and relay controller (Supplementary Figs. 67 and 68). As shown in Fig. 5c, the displayer can show specific afterglow color at corresponding temperature, and change ranging from yellow to green, by controllably tuning the heating and the lamp bead on-off through a microcontroller. As shown in Fig. 5d–k and Supplementary Fig. 69, versatile colorful afterglow patterns with contrast visual effect, including color gradients, indications, and numbers, were dynamically presented over time (Supplementary movie 2 and Supplementary Table 5), which offers the possibility for fabricating display and recording equipment with high spatial resolution and data capacity.

## Discussion

In conclusion, we reported abnormal thermally-stimulated dynamic organic phosphorescence in a series of organic molecules in crystal. The phosphorescence colors can be gradually tuned from yellow to cyan-blue with temperature increasing from 298 to 358 K. Combined with experimental results and theoretical simulation, we found that the variation of the excited molecular conformation played a vatial role in manipulating phosphorescence behavior by temperature, which was ascribed to the uneven intermolecular π-π interactions in crystal. In view of abnormal dynamic phosphorescence upon heating, the potential applications for smart pigment and colorful afterglow display were demonstrated. This finding not only breaks the traditional understanding of the influence of temperature on phosphorescence, but also outlines a principle to construct more functional phosphors for applications in optoelectronics.

## Methods

### Reagents and materials

Unless other noted, all the reagents and solvents used in the experiments were purchased from chemical sources without further treatment. All final products were purified by flash column chromatography and sublimation, and the resulting crystals were obtained using repeated recrystallization techniques of slow evaporation in ethyl acetate. For flash column chromatography, silica gel with 200–300 mesh was used.

### Calculation of the coefficient of thermal expansion (CTE)

Coefficient of thermal expansion of FPO, FMO and CPO crystals are calculated by the following equations.

1 $${\alpha }_{l}=\frac{\left(l-{l}_{0}\right)}{{l}_{0}\times \left(T-{T}_{0}\right)}$$

2 $${\alpha }_{V}=\frac{\left(V-{V}_{0}\right)}{{V}_{0}\times \left(T-{T}_{0}\right)}$$

Where *α*_*l*_ and *α*_*V*_ refer to linear and volume CTE, respectively. *l* and *V* are the length of crystal axis and volume at a certain temperature. *l*_0_ and *V*_0_ are the length of crystal axis and volume at initial temperature. *T* and *T*_0_ refer to a certain temperature and initial temperature.

### Calculation of RGB values

RGB value of afterglow color was obtained through the software of Adobe illustrator. We selected different points in the photo and calculated their RGB average.

### Supplementary information

Supplementary Information

Description of Additional Supplementary Files

Supplementary Movie 1

Supplementary Movie 2

### Source data

Source Data

## Data Availability

The authors declare that all other data supporting the findings of this study are provided in the Supplementary Information/Source Data file. Source data are provided with this paper. All other data are available from the corresponding author upon request. The X-ray crystallographic coordinates for structures reported in this study have been deposited at the Cambridge Crystallographic Data Centre (CCDC), under deposition numbers 2070267, 2283852, 2070541, 2070549, 2070551, 2070552, 2070555, 2283869, 2283870, 2071008, 2071009, 2071059, 2071411, 2116228, 2116229, 2116230, 2116231, 2116232, 2116233. These data can be obtained free of charge from The Cambridge Crystallographic Data Centre via www.ccdc.cam.ac.uk/data_request/cif. Source data are provided with this paper.
