# Supplementary material for: Abnormal thermally-stimulated dynamic organic phosphorescence
Source: Nat Commun. 2024 Mar 8;15:2134. doi: 10.1038/s41467-024-45811-0 (PMC10923930; doi:10.1038/s41467-024-45811-0)
Supplement: Supplementary file 1 — Supplementary Information [file 41467_2024_45811_MOESM1_ESM.pdf]

Supplementary Information for

Abnormal Thermally-stimulated Dynamic Organic Phosphorescence

He Wang,[1,†] Huili Ma,[1,†] Nan Gan,[2] Kai Qin,[3] Zhicheng Song,[1] Anqi Lv,[1] Kai Wang,[1] Wenpeng Ye,[1] Xiaokang Yao,[1] Chifeng Zhou,[1] Xiao Wang,[4] Zixing Zhou,[1] Shilin Yang,[3] Lirong Yang,[1] Cuimei Bo,[3] Huifang Shi,[1] Fengwei Huo,[1] Gongqiang Li,[1*] Wei Huang,[1, 2, 4, 5*] Zhongfu An[1, 4*]

[1]Key Laboratory of Flexible Electronics (KLoFE) & Institute of Advanced Materials (IAM), Nanjing Tech University, Nanjing, China.

[2] Frontiers Science Center for Flexible Electronics (FSCFE), MIIT Key Laboratory of Flexible Electronics (KLoFE), Northwestern Polytechnical University, Xi'an 710072, China.

[3]College of Electrical Engineering and Control Science, Nanjing Tech University, Nanjing, China.

[4]The Institute of Flexible Electronics (IFE, Future Technologies), Xiamen University, Xiamen 361005 Fujian, China.

[5]Key Laboratory for Organic Electronics and Information Displays & Institute of Advanced Materials (IAM), Nanjing University of Posts & Telecommunications, Nanjing, China.

†These authors contributed equally to this work.

**e-mail: iamzfan@njtech.edu.cn; iamgqli@njtech.edu.cn; vc@nwpu.edu.cn.*

Contents

I. **Experimental details**

II. **Photophysical properties of the reported and model phosphors**

III. **Single crystal data**

IV. **Density function theory (DFT) calculation**

V. **Expansion of abnormal thermally-stimulated dynamic organic phosphors**

VI. **Applications of thermally-stimulated dynamic organic phosphors**

VII. **Supplementary movies**

VIII. **References**

I. Experimental details

Measurements. Nuclear magnetic resonance (^{1}H and ^{13}C NMR) spectra were obtained on a Bruker Ultra Shield Plus 400 MHz spectrometer. Chemical shifts were calibrated by using tetramethylsilane (TMS) in deuterated solvents as the internal standard. Resonance patterns were noted by s (singlet), d (double), t (triplet), q (quartet), and m (multiplet). High performance liquid chromatography (HPLC) was performed using a SunFireTM C18 column conjugated to an ACQUITY UPLCH-class water HPLC system. Elemental analysis was conducted on a Vario EL Cube. The differential scanning calorimetry (DSC) measurements were performed on a TA Instrument DSC Q1000. Steady-state photoluminescence, phosphorescence, and excitation-phosphorescence emission mapping were measured using Hitachi F7100. Temperature dependent phosphorescence emission spectra from 198 to 363 K were measured using Hitachi F7100. Phosphorescent lifetimes, time-resolved emission spectra (TRES) were obtained on an Edinburgh FLS1000 fluorescence spectrophotometer equipped with a xenon arc lamp (Xe900) and a microsecond flash-lamp (μF900), respectively. Temperature dependent luminescent photographs and videos were taken on a heating stage by a Cannon EOS 700D camera under the irradiation of a hand-held UV lamp (365 nm). X-ray crystallography was completed using a Bruker SMART APEX-II CCD diffractometer with graphite monochromated Mo-K_α radiation.

Synthesis of 1-methyl-5-fluoro-1H-indole-2,3-dione (FMDO). 5-Fluoroindoline-2,3-dione (2 g, 12.11 mmol) was added into a mixture of iodomethane (5.16 g, 36.34 mmol) and K_2CO_3 (3.35 g, 24.22 mmol) in acetonitrile (50 mL). After that, the reaction mixture was stirred at 45 °C for 48 h. Then, the solvent was evaporated under vacuum, and the reaction crude was purified by column chromatography on silica gel to yield FMDO (2.09 g, 96.5%) as a red solid. ^{1}H NMR (400 MHz, $CDCl_3$) δ 7.36-7.24 (m, 2H), 6.89-6.82 (m, 1H), 3.25 (s, 3H).

Synthesis of 1-methyl-5-fluoro-1H-indole-2-one (FMO). FMDO (1.0 g, 5.58 mmol) was dissolved in 30 mL hydrazine hydrate (50%) and the reaction mixture was heated at 130 °C for 24 hours. Then, the solution was concentrated under reduced pressure, then extracted the residue with dichloroform (DCM, 20mL×3), the organic phase was dried over anhydrous Na_2SO_4. After filtration and removal of solvent, the product was purified by column chromatography on silica gel. Remove pigment by vacuum sublimation at 90 °C to give FMO (0.63g, 67.8%) as a light-yellow solid. ^{1}H NMR (400 MHz, $CDCl_3$) δ 7.07-6.89 (m, 2H), 6.71 (dd, *J*= 8.3, 4.2 Hz, 1H), 3.51 (s, 2H), 3.19 (s, 3H); ^{13}C NMR (101 MHz, $CDCl_3$): δ 174.61, 160.03, 158.12, 141.16, 126.00, 125.93, 114.13, 113.95, 112.61, 112.41, 108.40, 108.33, 36.00, 26.32. HPLC-MS (m/z): calcd for C_9H_8FNO, 165.06. Found: 165.92. Anal. Calculated for C_9H_8FNO: C, 65.45; H, 4.88; N, 8.48. Found: C, 65.47; H, 4.57; N, 8.40.

Synthesis of 1-ethyl-5-fluoro-1H-indole-2,3-dione (FEDO). 5-Fluoroindoline-2,3-dione (2.0 g, 12.11 mmol) was added into a mixture of iodoethane (5.67 g, 36.34 mmol) and K_2CO_3 (3.35 g, 24.22 mmol) in acetonitrile (50 mL). After that, the reaction mixture was stirred at 45 °C for 48 h. Then, the solvent was evaporated under vacuum, and the reaction crude was purified by column chromatography on silica gel to yield FEDO (2.18 g, 93.2%) as a red solid. ^{1}H NMR (400 MHz, $CDCl_3$) δ 7.32-7.29 (m, 2H), 6.86 (dd, *J*=8.0, 4.0 Hz, 1H), 3.79 (q, 2H), 1.31 (t, 3H).

Synthesis of 1-ethyl-5-fluoro-1H-indole-2-one (FEO). FEDO (1.0 g, 5.18 mmol) was dissolved in 30 mL hydrazine hydrate (50%) and the reaction mixture was heated at 130 °C for 24 hours. Then, the solution was concentrated under reduced pressure, then extracted the residue with DCM (20mL×3), the organic phase was dried over anhydrous Na_2SO_4. After filtration, the product was purified by column chromatography on silica gel. Remove pigment by vacuum sublimation at 80 °C to give product (0.60 g, 65%) as a light-yellow solid. ^{1}H NMR (400 MHz, $CDCl_3$) δ 7.05-6.88 (m, 2H), 6.73 (dd, *J*= 8.0, 4.0 Hz, 1H), 3.74 (q, *J*= 8.0 Hz, 2H), 3.50 (d, *J*=12.0 Hz, 2H), 1.26-1.22(t, *J*= 8.0 Hz, 3H); ^{13}C NMR (101 MHz, $CDCl_3$): δ 174.23, 159.86, 157.95, 140.23, 126.29, 126.22, 114.07, 113.88, 112.75, 112.56, 108.49, 108.42, 36.07, 34.79, 12.58. HPLC-MS (m/z): calcd for $C_{10}H_{10}FNO$, 179.07. Found: 179.93. Anal. Calculated for $C_{10}H_{10}FNO$: C, 67.03; H, 5.63; N, 7.82. Found: C, 67.02; H, 5.33; N, 7.83.

Synthesis of 1-propyl-5-fluoro-1H-indole-2,3-dione (FPDO). 5-Fluoroindoline-2,3-dione (2.0 g, 12.11 mmol) was added into a mixture of the reagent bromopropane (4.47 g, 36.34 mmol) and K_2CO_3 (3.35 g, 24.22 mmol) in acetonitrile (50 mL). After that, the reaction mixture was stirred at 45 °C for 48 h. Then, the solvent was evaporated under vacuum, and the reaction crude was purified by column chromatography on silica gel to yield FPDO (2.16 g, 86.3%) as a red solid. ^1H NMR (400 MHz, $CDCl_3$) δ 7.33 (dd, *J*=16.9, 8.7 Hz, 2H), 6.91 (dd, *J*=8.8, 3.6 Hz, 1H), 3.73 (t, *J*=7.3 Hz, 2H), 1.78 (q, *J*=7.4 Hz, 2H), 1.04 (t, *J*=7.4 Hz, 3H).

Synthesis of propyl-5-fluoro-1H-indole-2-one (FPO). FPDO (1.0 g, 5.18 mmol) was dissolved in 30 mL hydrazine hydrate (50%) and the reaction mixture was heated at 130 °C for 24 hours. Then, the solution was concentrated under reduced pressure, then extracted the residue with DCM (20mL×3), the organic phase was dried over anhydrous Na_2SO_4. After filtration, the product was purified by column chromatography on silica gel. Remove pigment by vacuum sublimation at 80 °C to give product (0.65 g, 69.2%) as a light-yellow solid. ^1H NMR (400 MHz, $CDCl_3$) δ 7.00-6.93 (m, 2H), 6.72 (dd, *J*=8.0, 4.0 Hz, 1H), 3.65 (t, *J*=4.0 Hz, 2H), 3.51 (s, 2H), 1.68 (m, 2H), 0.95 (t, *J*=8.0 Hz, 3H); ^{13}C NMR (101 MHz, $CDCl_3$): δ 174.57, 159.84, 157.93, 140.65, 126.19, 126.12, 114.04, 113.85, 112.68, 112.48, 108.67, 108.60, 41.72, 36.02, 20.69, 11.40. HPLC-MS (m/z): calcd for $C_{11}H_{12}FNO$, 193.09. Found: 193.94. Anal. Calculated for $C_{11}H_{12}FNO$: C, 68.38; H, 6.26; N, 7.25. Found: C, 68.37; H, 6.24; N, 7.26.

Synthesis of 1-ethyl-5-fluoro-1H-indole-2,3-dione (CEDO). 5-Chloroindoline-2,3-dione (2.0 g, 11.01 mmol) was added into a mixture of the reagent iodoethane (5.15 g, 33.03 mmol) and K_2CO_3 (3.04 g, 24.22 mmol) in acetonitrile (50 mL). After that, the reaction mixture was stirred at 45 °C for 48 h. Then, the solvent was evaporated under vacuum, then extracted the residue with DCM (20mL×3), the organic phase was dried over anhydrous Na_2SO_4. After filtration, the reaction crude was purified by column chromatography on silica gel to yield CEDO (1.93 g, 83.5%) as a red solid. ^1H NMR (400 MHz, $CDCl_3$) δ 7.56-7.53 (m, 2H), 6.86 (d, *J*=8.0 Hz, 1H), 3.77 (dd, *J*=12.0, 8.0 Hz, 2H), 1.29 (t, *J*=8.0 Hz, 3H).

Synthesis of 1-ethyl-5-fluoro-1H-indole-2-one (CEO). CEDO (1.0 g, 4.77 mmol) was dissolved in 30 mL hydrazine hydrate (50%) and the reaction mixture was heated at 130 °C for 24 hours. Then, the solution was concentrated under reduced pressure, then extracted the residue with DCM (20mL×3), the organic phase was dried over anhydrous Na_2SO_4. After filtration, the product was purified by column chromatography on silica gel. Remove pigment by vacuum sublimation at 85 °C to give product (0.58 g, 62%) as a light-yellow solid. ^1H NMR (400 MHz, $CDCl_3$) δ 7.25 (s, 1H), 7.23 (d, *J*=78.0 Hz, 1H), 6.74 (d, *J*=8.0 Hz, 1H), 3.74 (q, *J*=8.0 Hz, 2H), 3.50 (s, 2H), 1.24 (t, *J*=8.0 Hz, 3H); ^{13}C NMR (101 MHz, $CDCl_3$): δ 174.16, 142.94, 127.82, 127.52, 126.43, 125.02, 109.12, 35.80, 34.87, 12.66. HPLC-MS (m/z): calcd for $C_{10}H_{10}FNO$, 195.05. Found: 195.91. Anal. Calculated for $C_{10}H_{10}FNO$: C, 61.39; H, 5.15; N, 7.16. Found: C, 61.47; H, 5.28; N, 7.04.

Synthesis of 1-propyl-5-fluoro-1H-indole-2,3-dione (CPDO). 5-Chloroindoline-2,3-dione (2.0 g, 11.01 mmol) was added into a mixture of the reagent bromopropane (4.06 g, 33.03 mmol) and K_2CO_3 (3.04 g, 22.02 mmol) in acetonitrile (50 mL). After that, the reaction mixture was stirred at 45 °C for 48 h. Then, the solvent was evaporated under vacuum, then extracted the residue with DCM (20mL×3), the organic phase was dried over anhydrous Na_2SO_4. After filtration, the reaction crude was purified by column chromatography on silica gel to yield CPDO (2.02 g, 82%) as a red solid. ^1H NMR (400 MHz, $CDCl_3$) δ 7.60-7.48 (m, 2H), 6.85 (d, *J*=8.0 Hz, 1H), 3.67 (t, *J*= 8.0 Hz, 2H), 1.71 (m, 2H), 0.98 (t, *J*=8.0 Hz, 3H).

Synthesis of propyl-5-fluoro-1H-indole-2-one (CPO). CPDO (1.0 g, 4.47 mmol) was dissolved in 30 mL hydrazine hydrate (50%) and the reaction mixture was heated at 130 °C for 24 hours. Then, the solution was concentrated under reduced pressure, then extracted the residue with DCM (20mL×3), the organic phase was dried over anhydrous Na_2SO_4. After filtration, the product was purified by column chromatography on silica gel. Remove pigment by vacuum sublimation at 90 °C to give product (0.59 g, 63%) as a light-yellow solid. ^1H NMR (400 MHz, $CDCl_3$) δ 7.22 (m, 2H), 6.73 (d, *J*=8.0 Hz, 1H), 3.64 (t, *J*= 8.0 Hz, 2H), 3.50 (s, 2H), 1.67 (m, 2H), 0.95 (t, *J*=8.0 Hz, 3H); ^{13}C NMR (101 MHz, $CDCl_3$): δ 174.54, 143.36, 127.81, 127.51, 126.34, 124.99, 109.25, 41.78, 35.74, 20.77, 11.46. HPLC-MS (m/z): calcd for $C_{11}H_{12}FNO$, 209.06.

Found: 209.94. Anal. Calculated for $C_{11}H_{12}FNO$: C, 63.01; H, 5.77; N, 6.68. Found: C, 62.97; H, 5.90; N, 6.53.

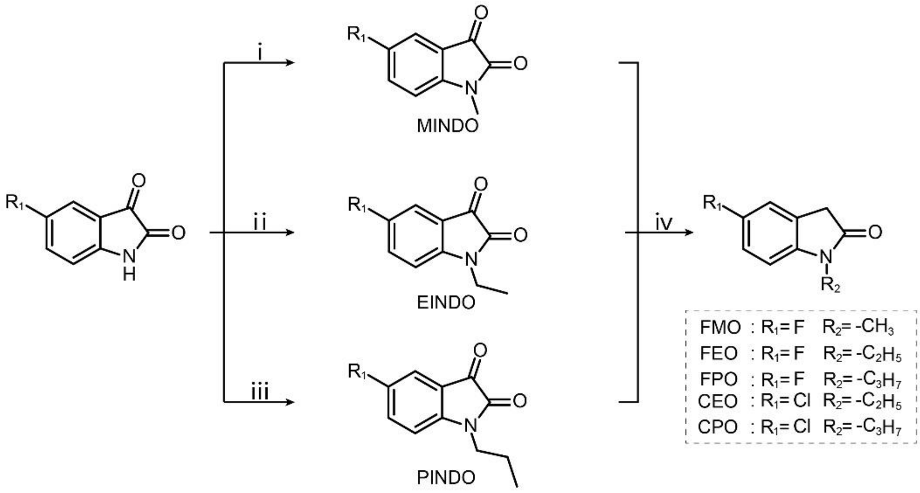

Supplementary Figure 1. Synthetic routes of dynamic organic phosphors. (i) 3 equiv. iodomethane, acetonitrile, 45 °C, 48 h; (ii) 3 equiv. iodoethane, acetonitrile, 45 °C, 48 h; (iii) 3 equiv. bromopropane, acetonitrile, 45 °C, 48 h; (iv) hydrazine hydrate (50%), 130 °C, 24 h.

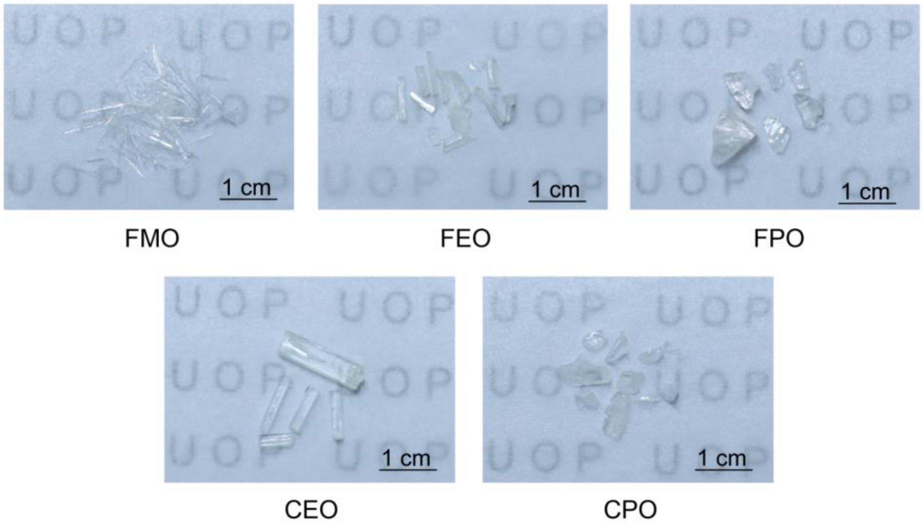

Supplementary Figure 2. The photographs of FMO, FEO, FPO, CEO, and CPO phosphors in crystal state under ambient light.

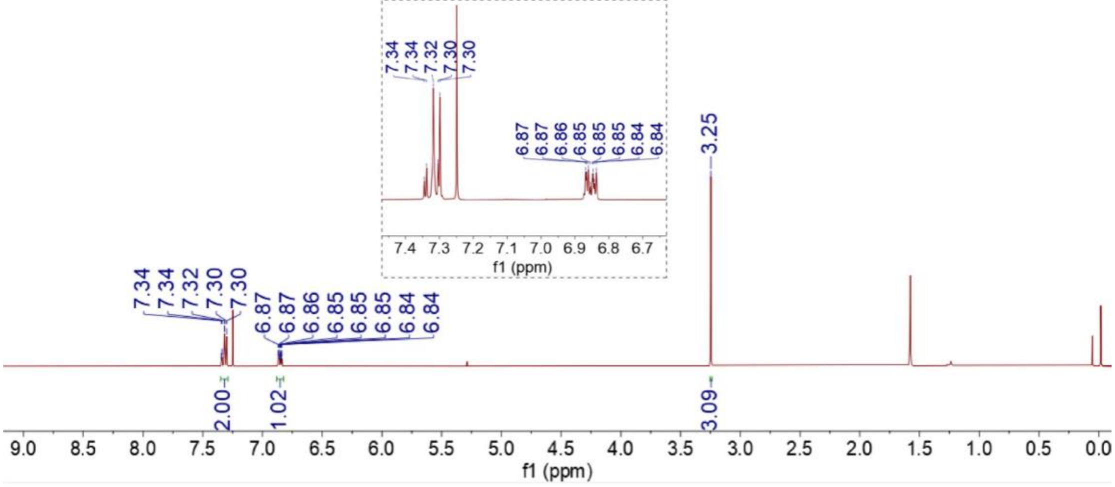

Supplementary Figure 3. The ^{1}H NMR spectrum of FMDO molecule in $CDCl_3$.

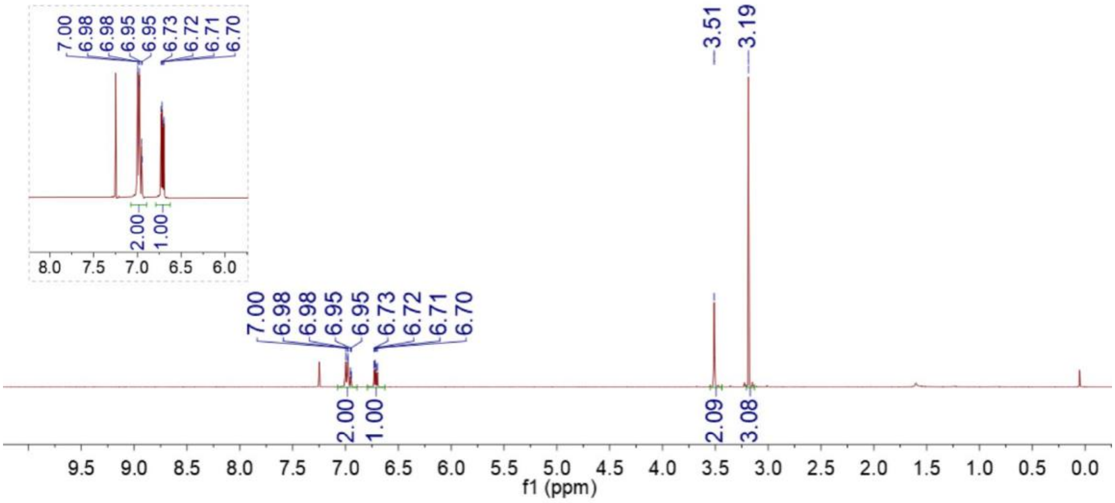

Supplementary Figure 4. The ^{1}H NMR spectrum of FMO molecule in $CDCl_3$.

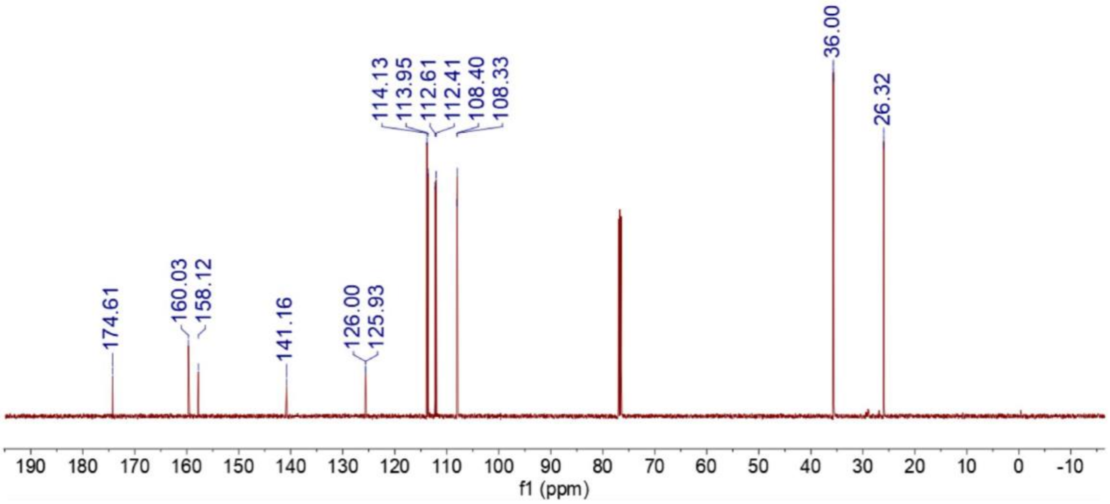

Supplementary Figure 5. The ^{13}C NMR spectrum of FMO molecule in $CDCl_3$.

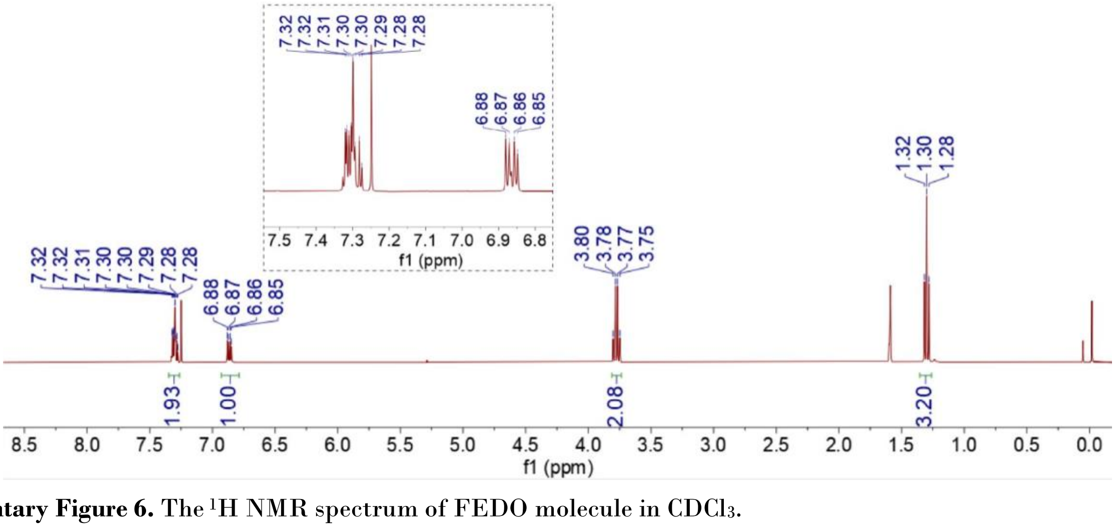

Supplementary Figure 6. The ^{1}H NMR spectrum of FEDO molecule in $CDCl_3$.

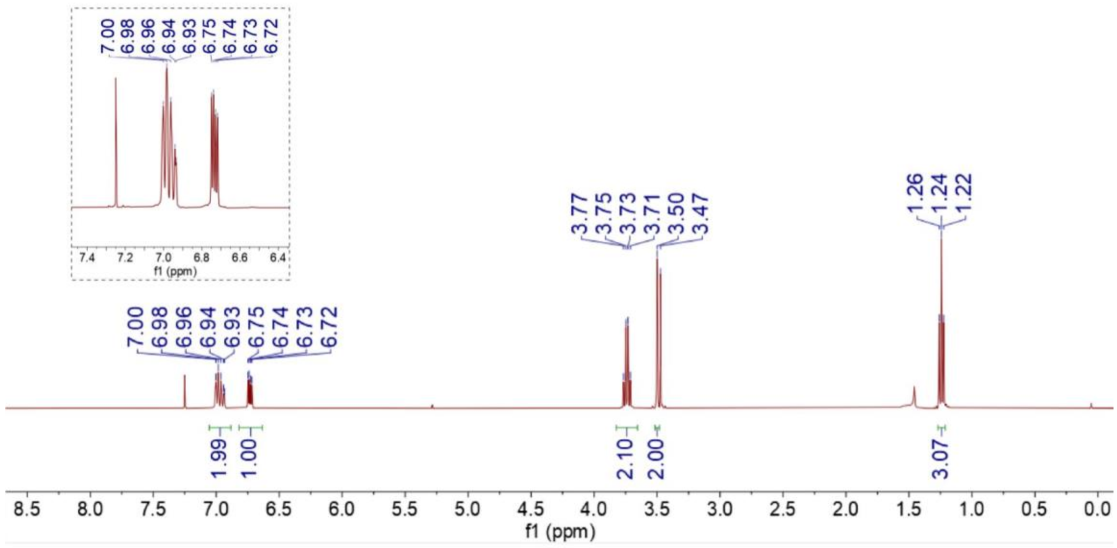

Supplementary Figure 7. The ^{1}H NMR spectrum of FEO molecule in $CDCl_3$.

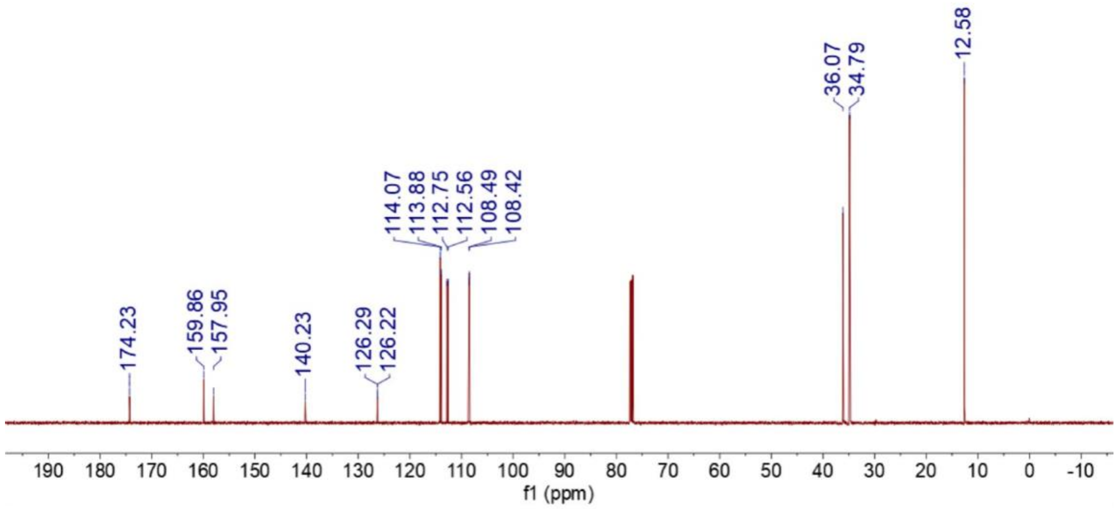

Supplementary Figure 8. The ^{13}C NMR spectrum of FEO molecule in $CDCl_3$.

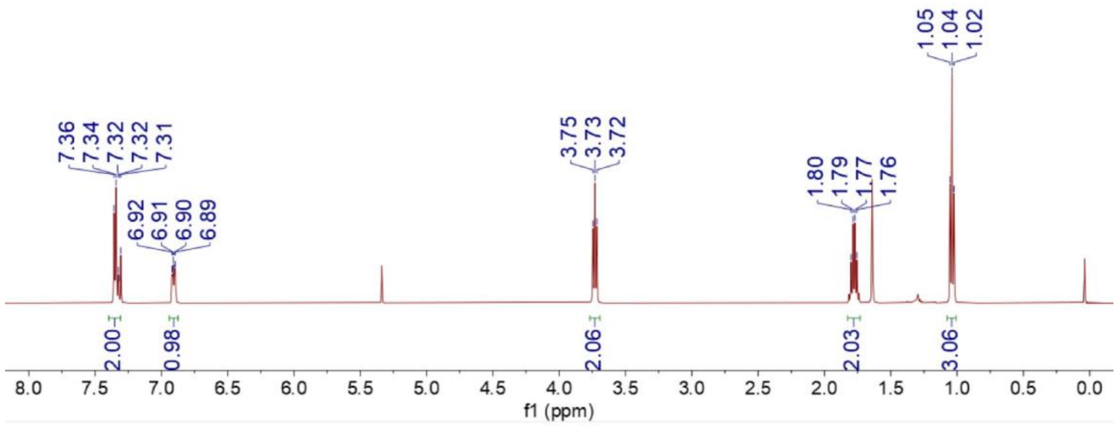

Supplementary Figure 9. The ^{1}H NMR spectrum of FPDO molecule in $CDCl_3$.

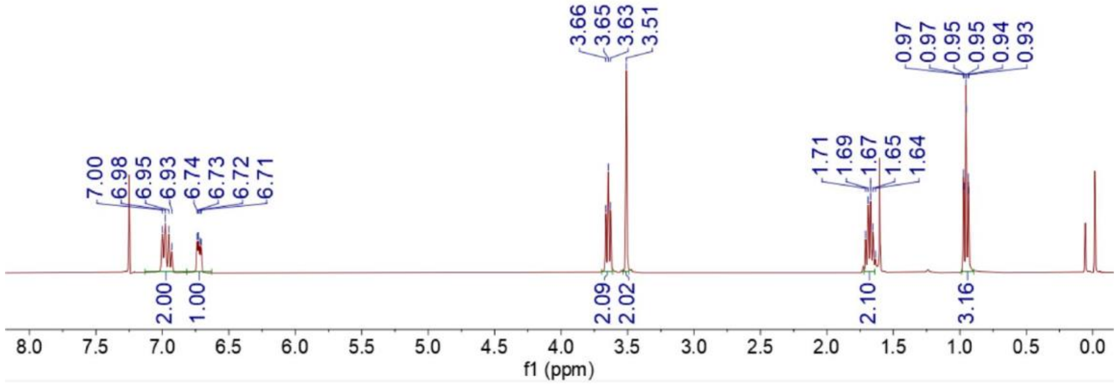

Supplementary Figure 10. The ^{1}H NMR spectrum of FPO molecule in $CDCl_3$.

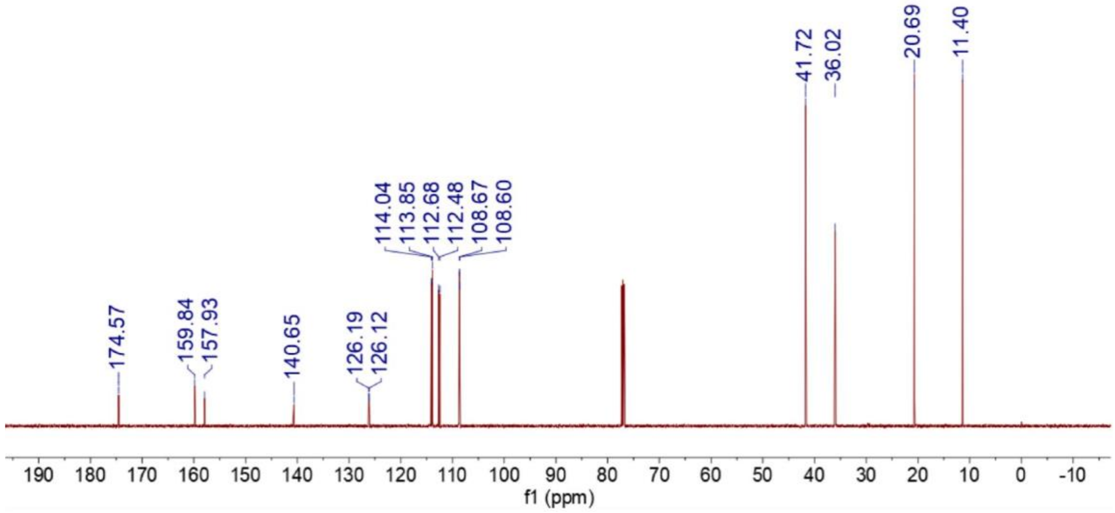

Supplementary Figure 11. The ^{13}C NMR spectrum of FPO molecule in $CDCl_3$.

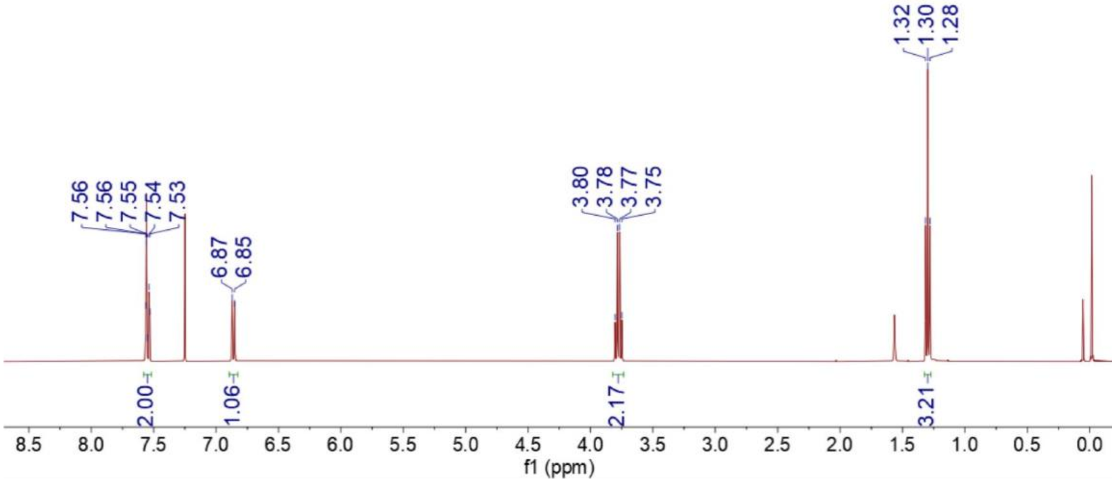

Supplementary Figure 12. The ^{1}H NMR spectrum of CEDO molecule in $CDCl_3$.

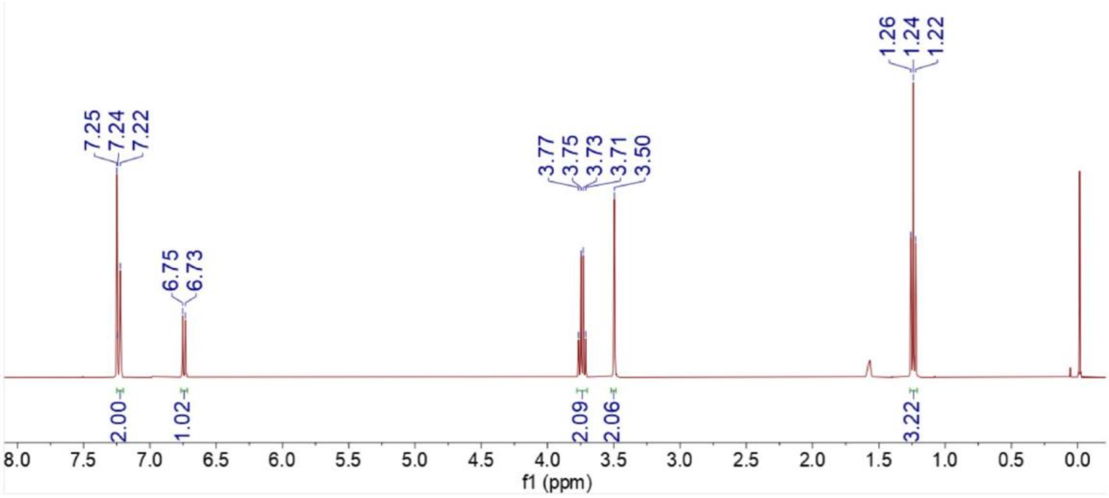

Supplementary Figure 13. The ^{1}H NMR spectrum of CEO molecule in $CDCl_3$.

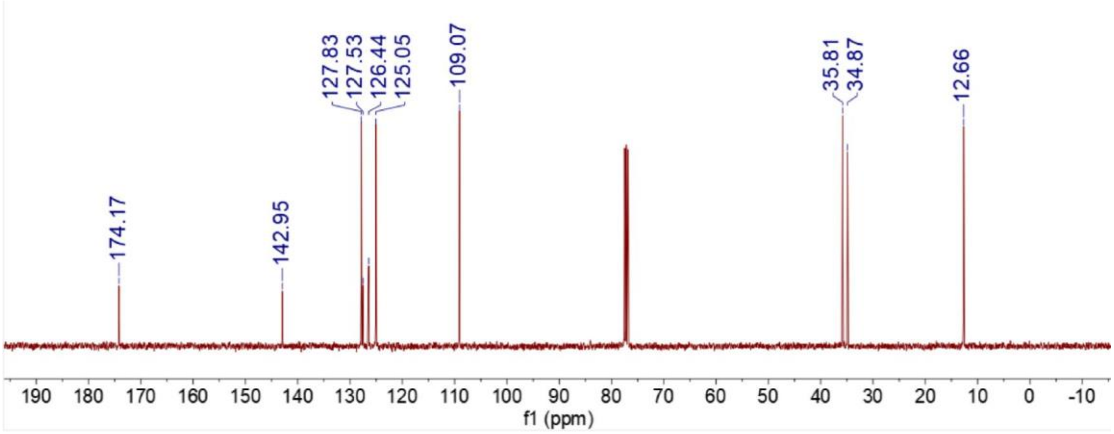

Supplementary Figure 14. The ^{13}C NMR spectrum of CEO molecule in $CDCl_3$.

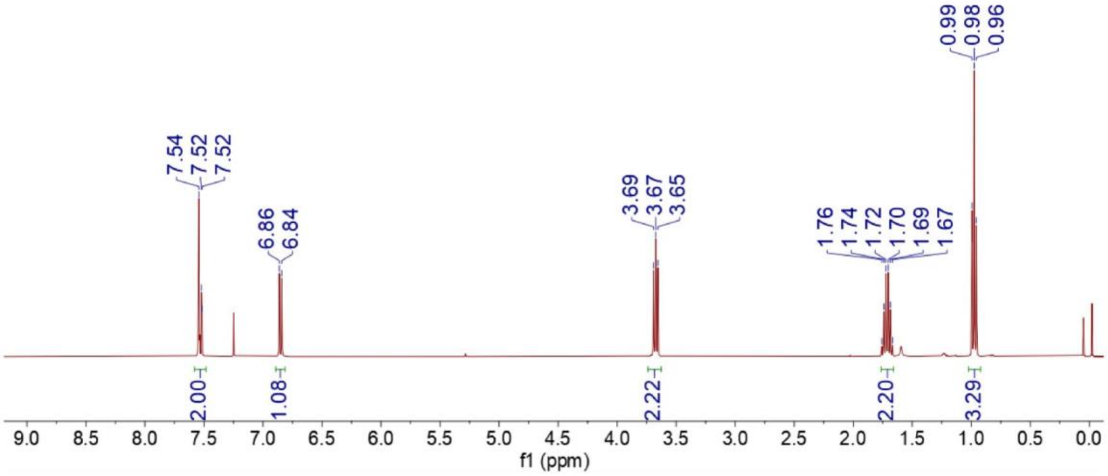

Supplementary Figure 15. The ^{1}H NMR spectrum of CPDO molecule in $CDCl_3$.

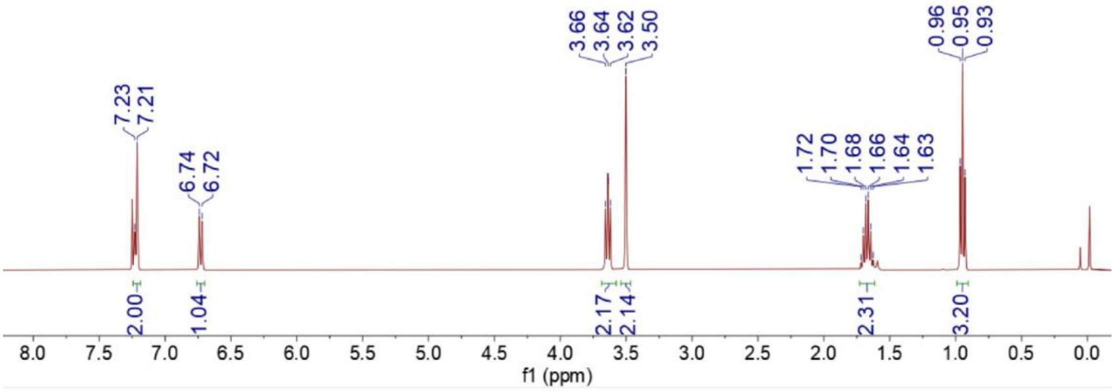

Supplementary Figure 16. The ^{1}H NMR spectrum of CPO molecule in $CDCl_3$.

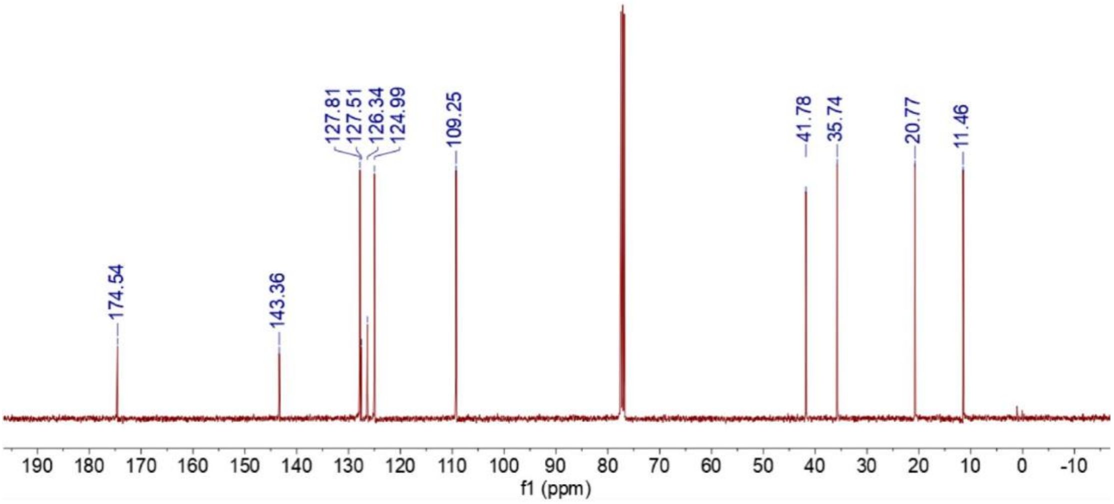

Supplementary Figure 17. The ^{13}C NMR spectrum of CPO molecule in $CDCl_3$.

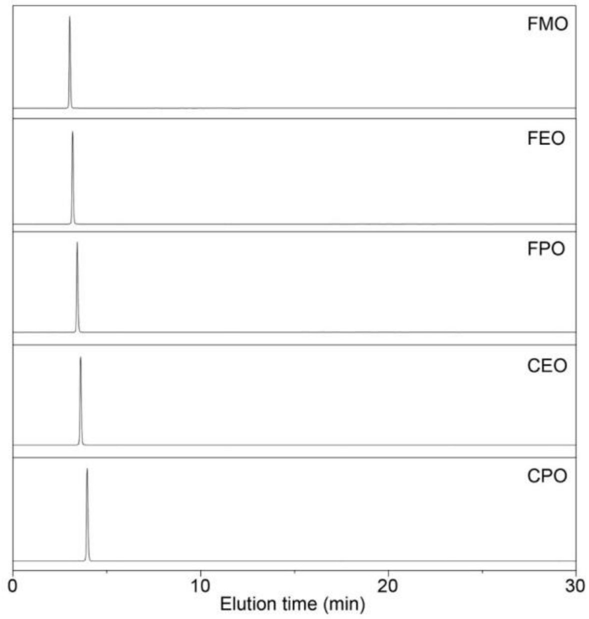

Supplementary Figure 18. High performance liquid chromatographs (HPLC) of FMO, FEO, FPO, CEO and CPO molecules in 10/90 (v/v) of water/methanol elution for 30 min.

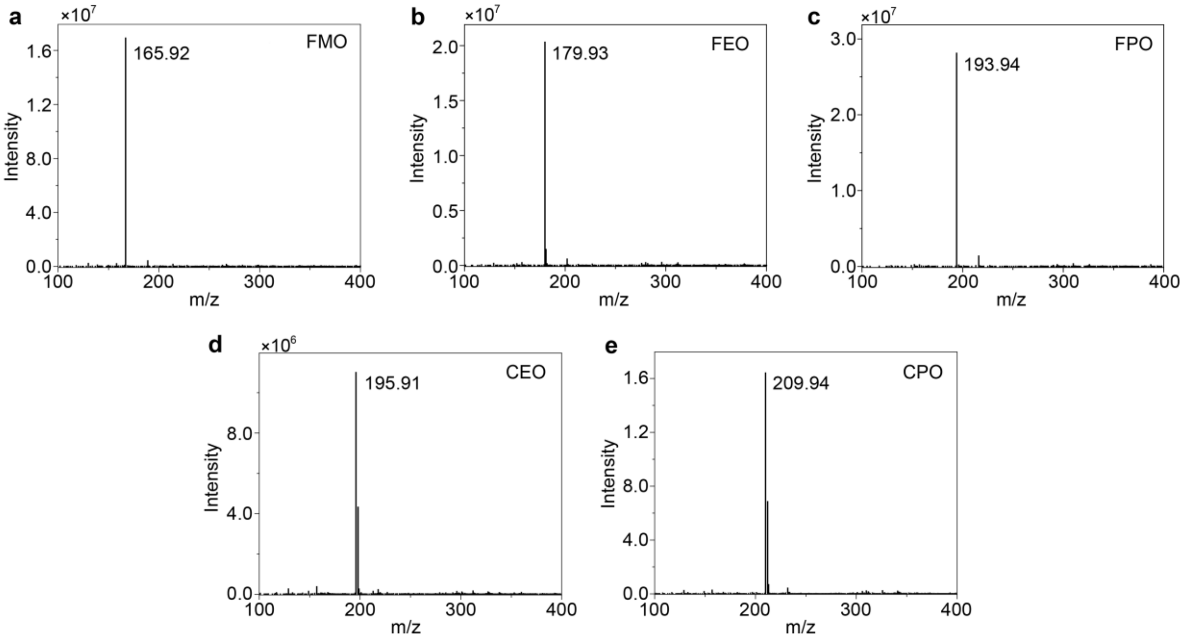

Supplementary Figure 19. HPLC-MS spectra of FMO (a), FEO (b), FPO (c), CEO (d) and CPO (e) molecules in 10/90 (v/v) of water/methanol elution.

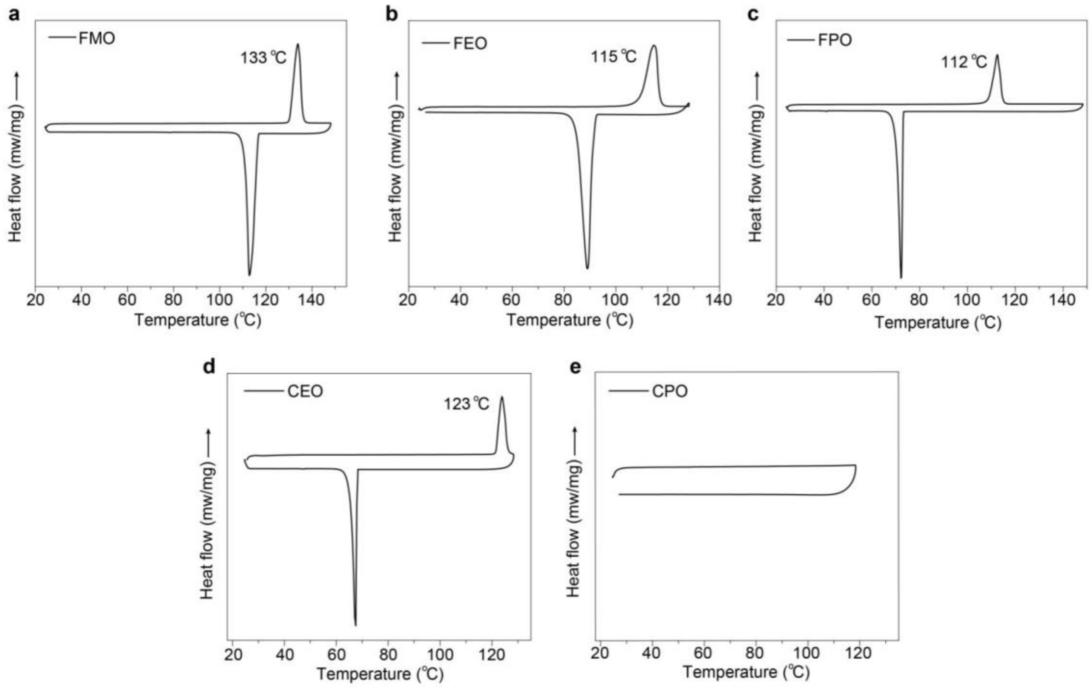

Supplementary Figure 20. Differential scanning calorimetry trace of FMO (a), FEO (b), FPO (c), CEO (d) and CPO (e) phosphors recorded under nitrogen at a heating rate of 10 ℃ /min.

II. Photophysical properties of the reported and model phosphors

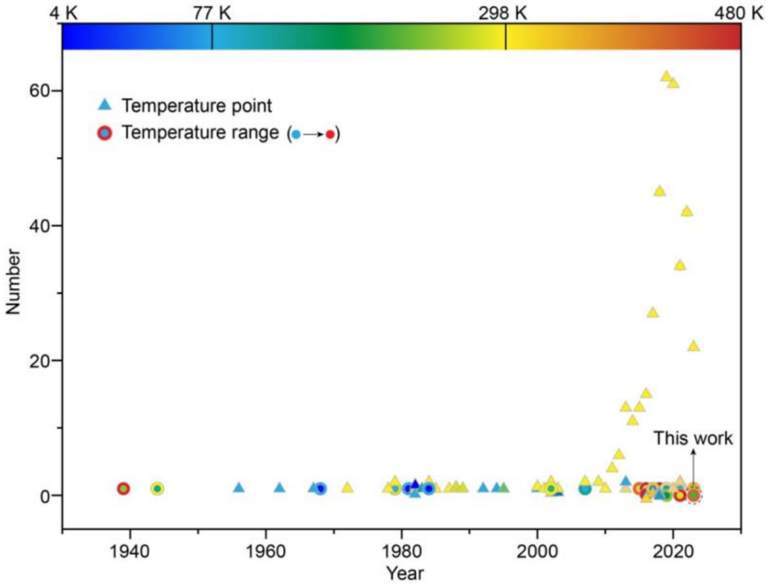

Supplementary Figure 21. Survey of temperature-dependent phosphorescence in purely organic materials in reported works and this article from 1939 to 2023[1-351]**.**

Taking the reported 2,4,6-trimethoxy-1,3,5-triazine (TMOT)[55] molecule as a control molecule, we found that it exhibited even π-π stacking along **b** axis and same intermolecular interactions between molecular layers (Supplementary Figure 22a and 22b). There is no driving force to induce the deformation of excited state molecules as temperature increasing, owing to the similar molecular environment between molecular layers. Therefore, like traditional organic phosphorescent materials, the TMOT molecule only presented luminescence quenching at high temperature without abnormal thermally-stimulated dynamic phosphorescence behavior (Supplementary Figure 22c).

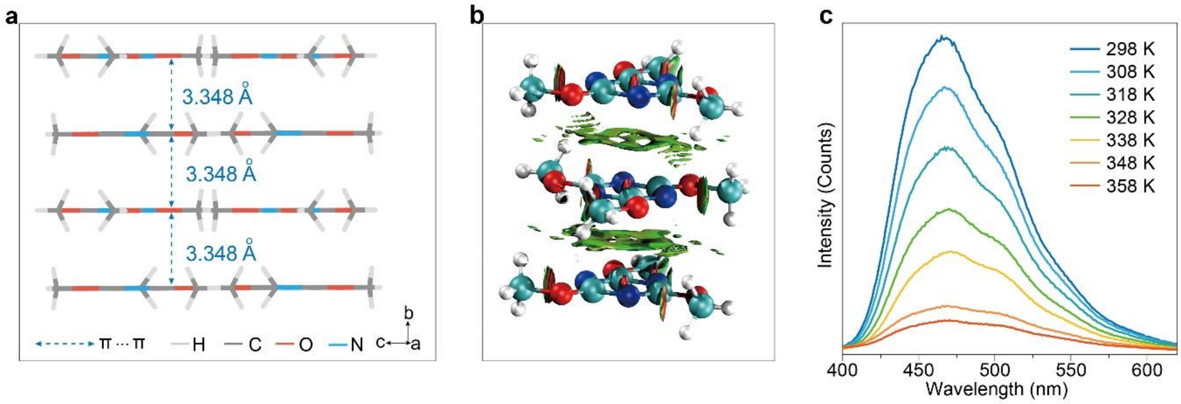

Supplementary Figure 22. Crystal structure and temperature dependent phosphorescence properties of TMOT phosphor in crystal. a, Molecular stacking of the TMOT single crystal viewed along **a** axis. **b**, Calculated intermolecular interactions (green iso-surface) in a trimer model. **c**, Phosphorescence spectra of TMOT crystal at different temperatures.

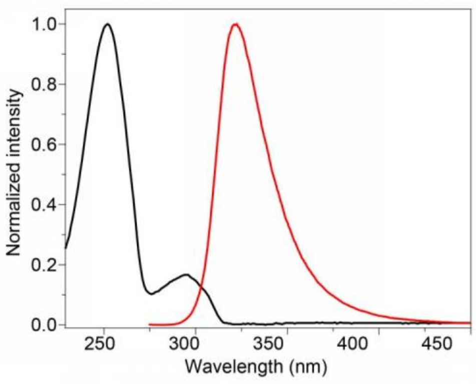

Supplementary Figure 23. Normalized UV absorption (black line) and steady-state photoluminescence (red line) spectra of FPO molecule in dichloromethane (1×10^{-5} M) under ambient conditions.

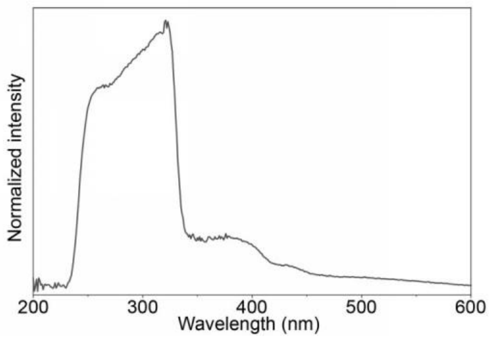

Supplementary Figure 24. Normalized UV absorption of the FPO phosphor in crystal state under ambient conditions.

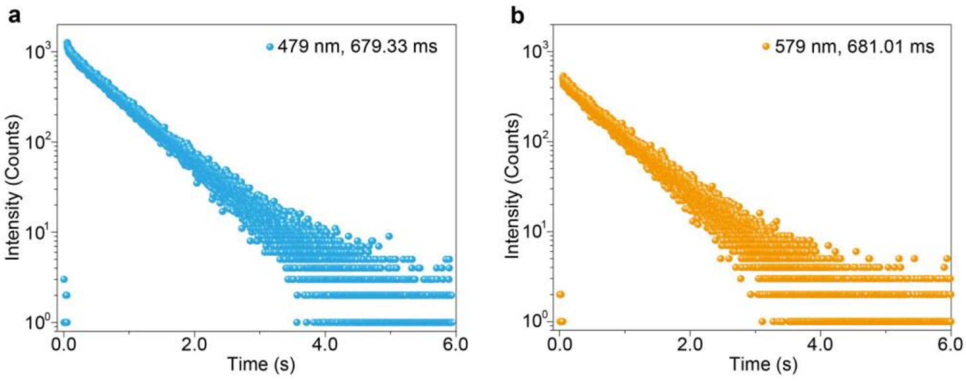

Supplementary Figure 25. Time resolved decay curves of the FPO phosphor monitoring at 479 (a) and 579 nm (b) under ambient conditions.

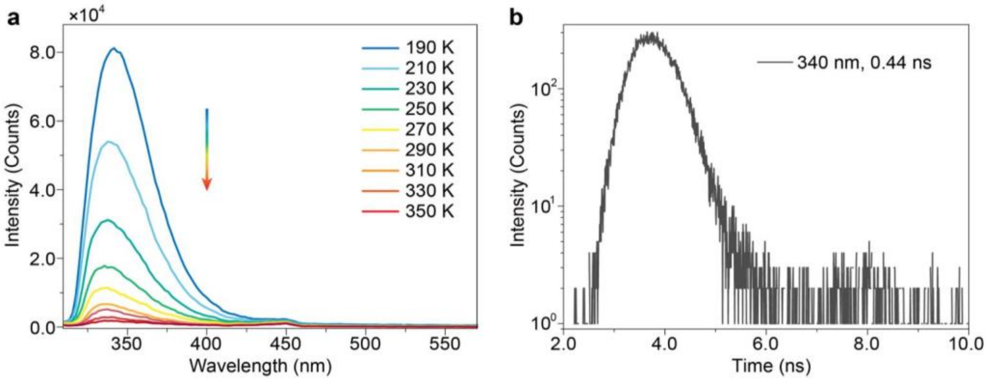

Supplementary Figure 26. a, Temperature dependent photoluminescence spectra of the FPO phosphor excited by 290 nm. b, Fluorescence lifetime decay curve monitoring at 340 nm of the FPO phosphor.

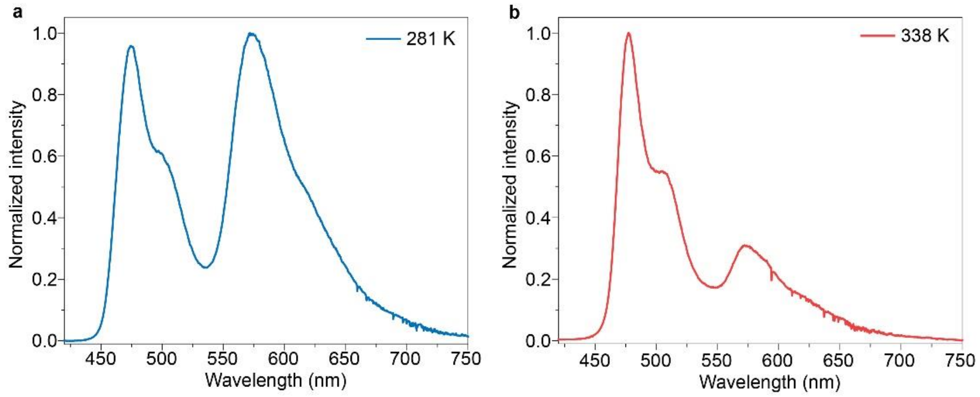

Supplementary Figure 27. Phosphorescence spectra of FPO crystal at 281 K (a) and 338 K (b) in a vacuum environment.

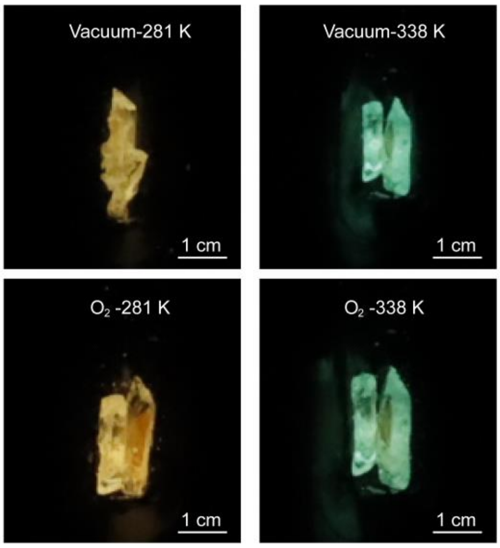

Supplementary Figure 28. Afterglow photographs of FPO crystal in vacuum and O_2 atmosphere from 281 to 338 K.

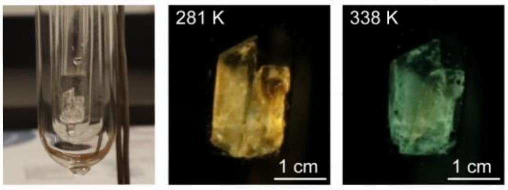

Supplementary Figure 29. Afterglow photographs of FPO crystal at 281 and 338 K in water environment.

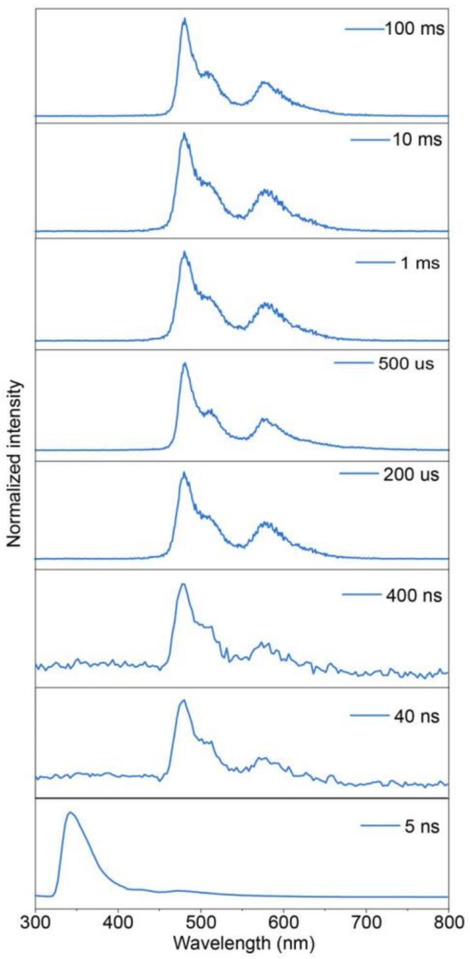

Supplementary Figure 30. **Time-resolved spectra of the FPO phosphor.** Time-resolved spectra of the FPO phosphor. Note that the time-resolved spectra with the delayed times from 5 ns to 400 ns were excited using a nanosecond hydrogen flash-lamp, while the delayed spectra from 200 μs to 100 ms were collected by a microsecond flash-lamp (μF900).

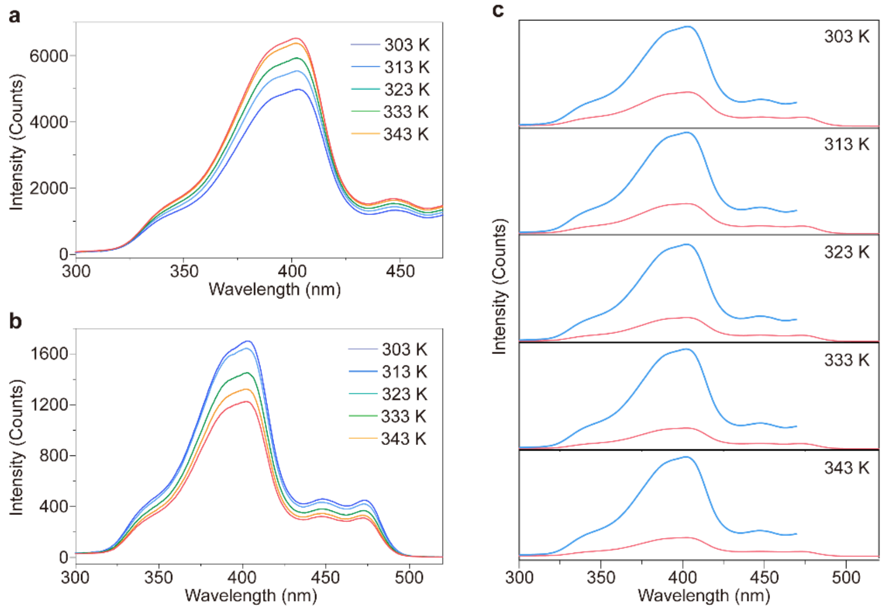

Supplementary Figure 31. Excitation spectra of phosphorescence emissions at 479 (a) and 579 (b) nm from 303 to 343 K. (c) Superposition of both excitation spectra at the same temperatures.

As shown in Supplementary Figure 31a, the intensity of the excitation spectra for phosphorescence emissions at 479 increases with temperature change from 303 to 343 K. For excitation spectra of the emission at 579 nm, the intensity

decreases under the same conditions (Supplementary Figure 31b). Taking the absorption spectrum in solution together, we concluded that the intermolecular aggregates make a great contribution to the phosphorescence emission bands with peaks at 479 and 579 nm. From Supplementary Figure 31c, it is easily found that there indeed existed different proportions for the presence of dimers and trimers as the temperature varied.

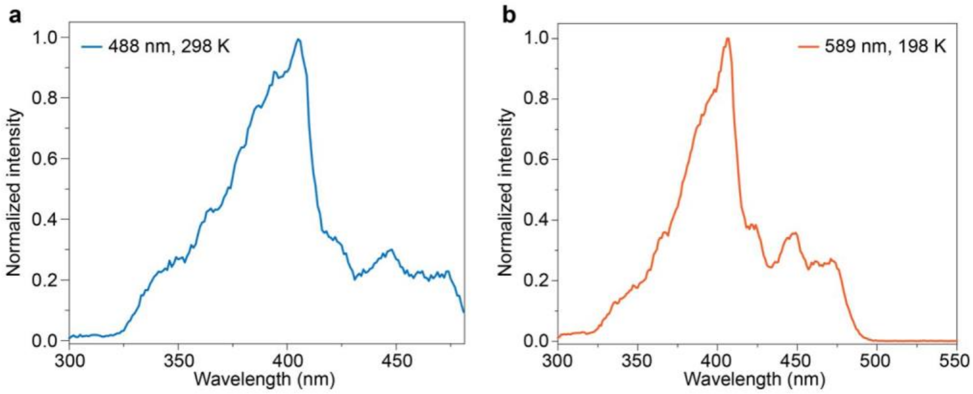

Supplementary Figure 32. Excitation spectra of phosphorescence emissions at 488 nm at 298 K (a) and at 589 nm at 198 K (b) for the FPO phosphor.

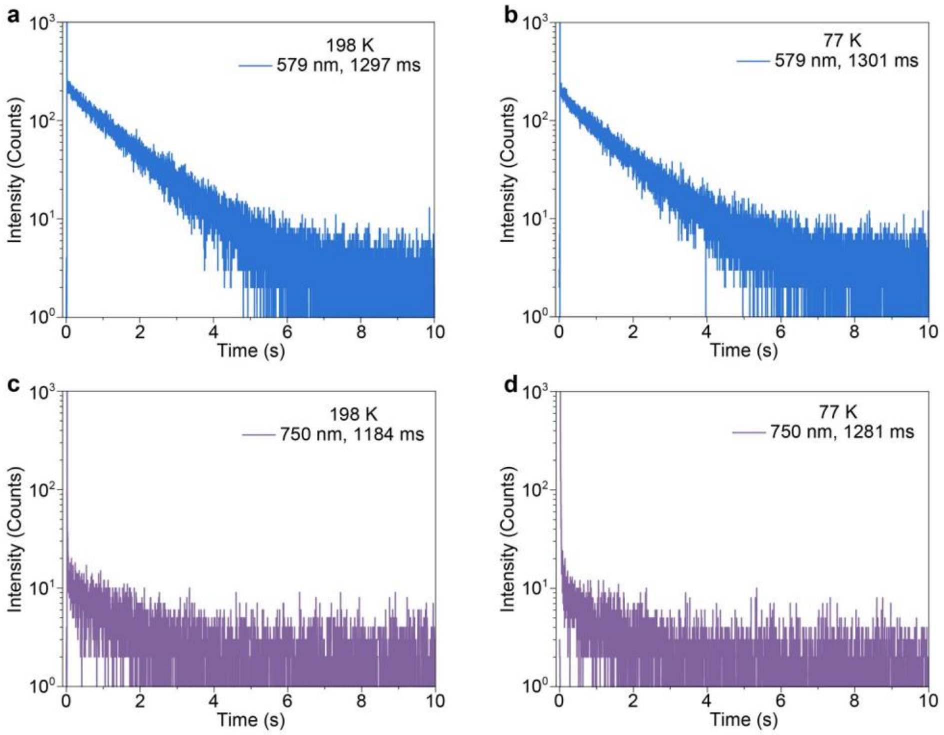

Supplementary Figure 33. Time resolved decay curves of the FPO phosphor monitoring at 579 (a, b) and 750 nm (c, d) at 77 K and 198 K, respectively.

As shown in Supplementary Figure 33, even if the emission at 750 nm is much weaker than that at 579 nm, the lifetimes of emission at 579 and 750 nm are almost consistent at 77 or 198 K, indicating that the phosphorescence characteristics of the emission at 579 and 750 nm are identical.

III. Single crystal data.

Supplementary Table 1. Structure data of the FPO single crystal at 90, 198, 273, 300, 320, 340 and 360 K.

**Formula**	$C_{11}H_{12}FNO$						
**Temperature**	90 K	198 K	273 K	300 K	320 K	340 K	360 K
**Space Group**	P 2_1/n	P 2_1/n	P 2_1/n	P 2_1/n	P 2_1/n	P 2_1/n	P 2_1/n
**Cell Lengths (Å)**	a=7.513(3)<br>b=10.918(4)<br>c=11.837(5)	a=7.590(2)<br>b=10.947(3)<br>c=11.940(4)	a=7.663(14)<br>b=10.962(2)<br>c=12.001(3)	a=7.682(11)<br>b=10.968(17)<br>c=12.025(2)	a=7.7055(6)<br>b=10.970(8)<br>c=12.041(10)	a=7.731(5)<br>b=10.979(8)<br>c=12.072(10)	a=7.757(8)<br>b=10.981(12)<br>c=12.096(15)
**Cell Angles (°)**	α=90<br>β=107.1(10)<br>γ=90	α=90<br>β=107.4(3)<br>γ=90	α=90<br>β=107.4(7)<br>γ=90	α=90<br>β=107.5(5)<br>γ=90	α=90<br>β=107.5(3)<br>γ=90	α=90<br>β=107.5(3)<br>γ=90	α=90<br>β=107.5(4)<br>γ=90
**Cell Volume ($Å^3$)**	927.883	946.962	961.846	966.507	970.967	977.225	982.626
**Z, Z'**	Z:4; Z':0	Z:4; Z':0	Z:4; Z':0	Z:4; Z':0	Z:1; Z':0	Z:4; Z':0	Z:4; Z':0
**Density (g/cm^3)**	1.383	1.355	1.334	1.328	1.322	1.313	1.306
**CCDC number**	2070267	2283852	2070541	2070549	2070551	2070552	2070555

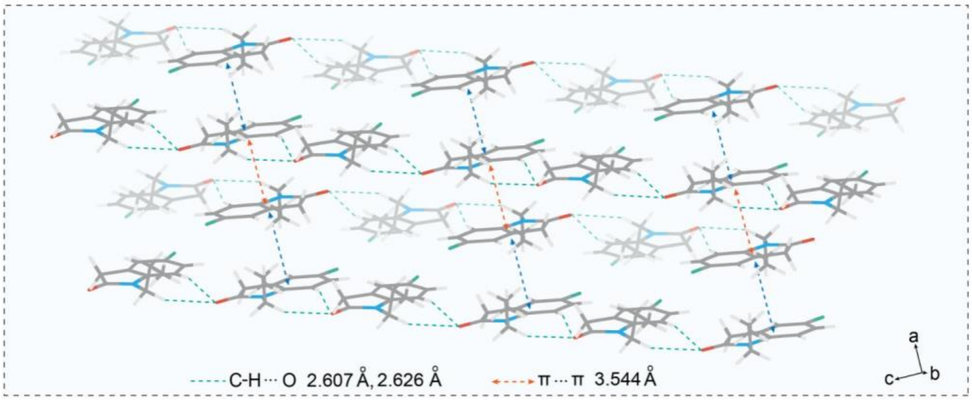

Supplementary Figure 34. Intermolecular stacking of FPO in crystal at 300 K.

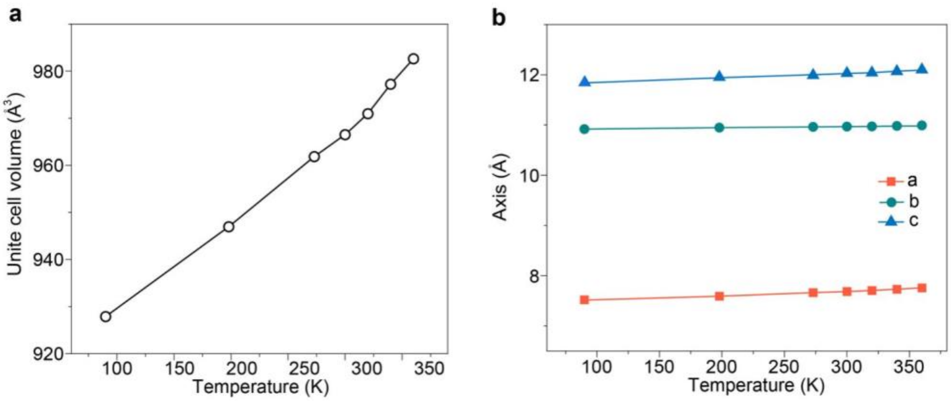

Supplementary Figure 35. Effect of temperature on structural parameters of the FPO crystal. a, Variation of cell volume. **b**, Edge variation along the crystallographic **a**, **b**, and **c** axis.

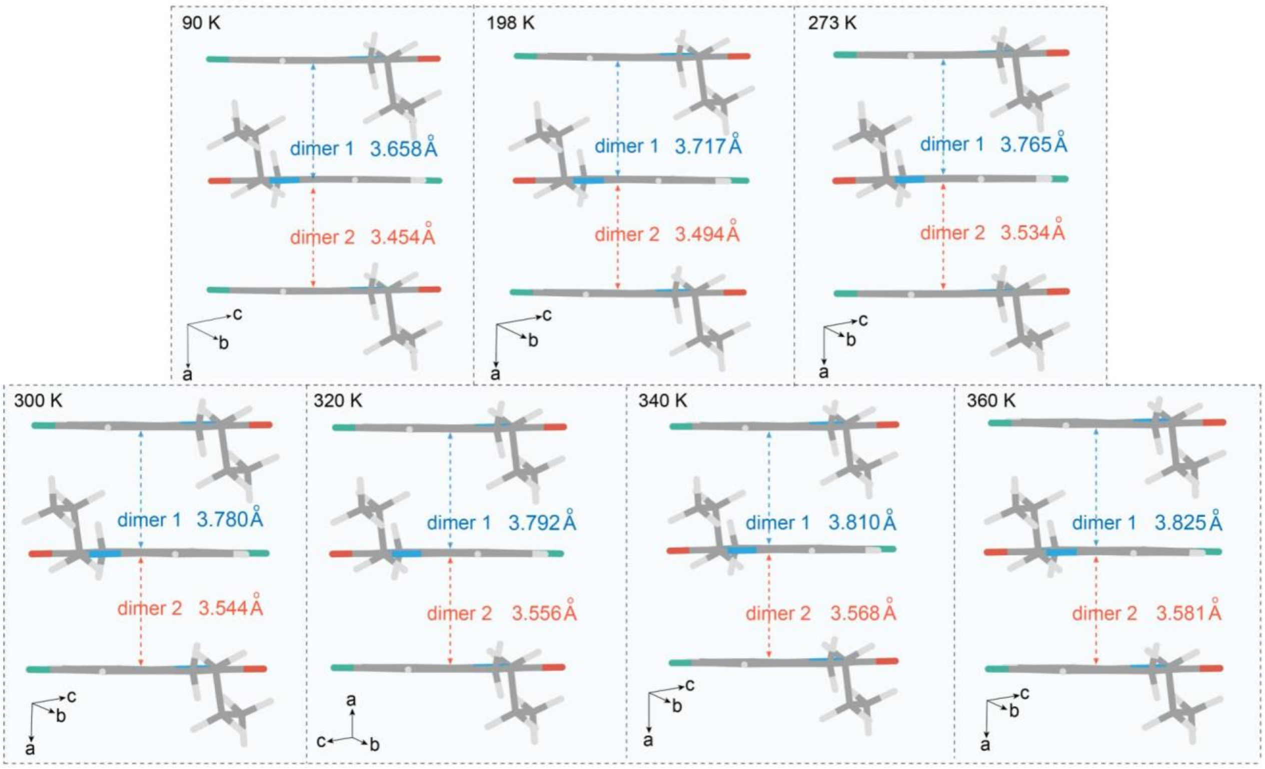

Supplementary Figure 36. Intermolecular π-π stacking in FPO crystal at different temperatures from 90 to 360 K.

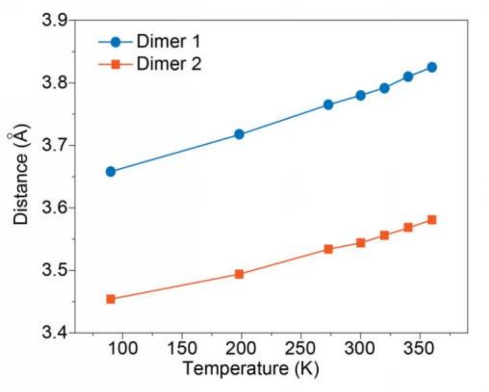

Supplementary Figure 37. Intermolecular π-π distance variation versus temperature in FPO crystal.

IV. Density function theory (DFT) calculation.

For model systems, the FPO, FEO, FMO, CPO, CEO, and TMOT molecules were extracted from the crystal structures. The electrostatic potential (ESP) on molecular surface was expressed by Multiwfn and VMD program[352]. Significant surface local minima and maxima of ESP are represented as cyan and yellow spheres, respectively. The unit is in kcal/mol. In the case of the trimer and tetramer for TMOT and FPO molecules, the interactions of contiguous molecules were observed by same program above[353]. All equilibrium molecules of FPO, FMO and CPO in ground states (S_0) were optimized based on QM/MM model by using the B97X-D functional together with the def2-SVP basis set, while the lowest triplet states (T_1) were obtained using unrestricted density functional theory (UDFT) at the same level. The calculation procedures of FPO, FMO and CPO were conducted by the quantum mechanics/molecular mechanics model (QM/MM) theory with two-layer ONIOM method in a 3×5×3, 3×4×4, and 4×3×4 supercells, respectively (Supplementary Figure 38, Supplementary Figure 59 and 60). The central two/three molecules were chosen as the QM parts, and the remaining molecules were defined as rigid MM parts modeled by the GAFF. Based on the T_1 geometries, we then evaluated the excited-state electronic structures, including excitation energies, weight factors and nature transition orbitals (NTOs) of low-lying

excited states with a Time-Dependent Density Functional Theory (TD-DFT) approach at the B97X-D/def2-SVP level. The above results are calculated by Gaussian 09 package[354].

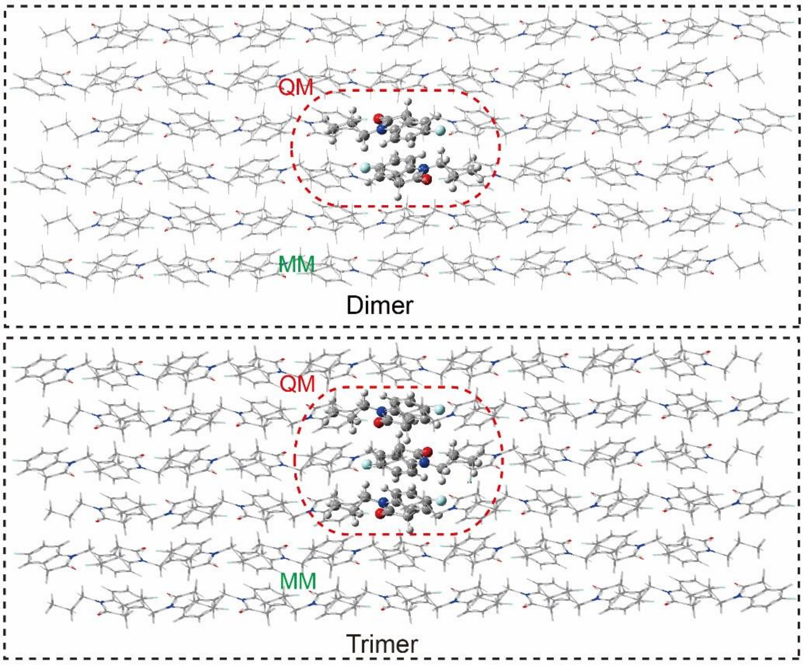

Supplementary Figure 38. Setup of QM/MM model for the FPO phosphor.

The lowest triplet excited state (T_1) of FPO phosphor in different aggregated states were calculated, respectively. As shown in Supplementary Figure 39, there are two types of S_1 and T_1 states (dimer and tetramer & trimer and pentamer), which exhibit similar S_1 and T_1 energy levels and molecular orbitals distributions. Moreover, there are two main different molecular conformations in these aggregates, which is also identical to that in dimer and trimer states. In addition, different types of dimer and trimer were also considered, which was shown in Supplementary Figure 40. Because the intermolecular interaction in the horizontal direction of molecules is much smaller than that in the π-π stacking direction, dimer 1 and trimer 1 are first excluded. Compared to dimer 3, dimer 2 exhibits closer intermolecular distance and stronger interplay. Therefore, we selected this type of dimer as a model to investigate. As shown in Supplementary Figure 41, the molecular orbit shape and T_1 energy (2.92 eV) of trimer 3 are similar to that of dimer 2 (E_{T1}=2.89 eV) in Figure 3d. Notably, trimer 3 also exhibits similar changes in molecular conformation to that of dimer 2, which further validates the mechanism of conformation-dependent triplet states. The same is true for dimer 3 and trimer 2. Altogether, above results demonstrated the conformation-dependent triplet state is a plausible mechanism for abnormal thermally-stimulated dynamic phosphorescence.

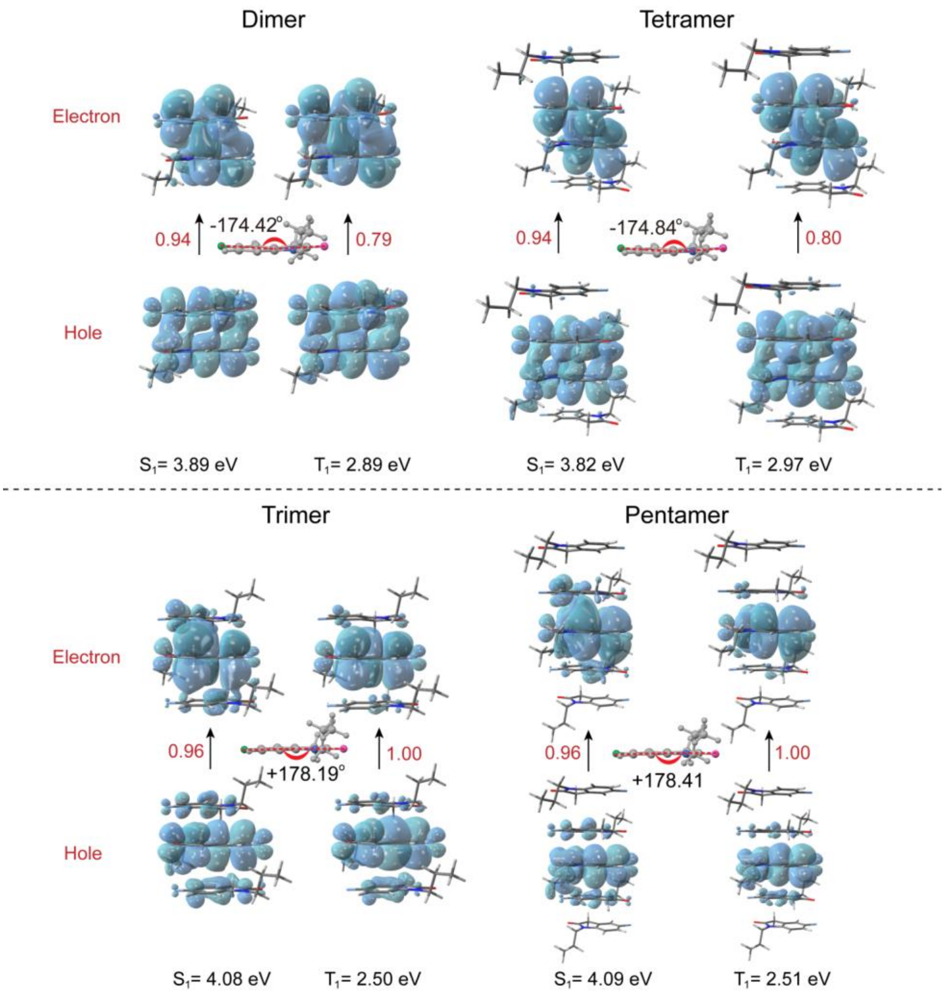

Supplementary Figure 39. Natural transition orbitals (NTOs) and weight factors contributing to the lowest-energy singlet and triplet transitions of FPO monomer, dimer, and trimer, tetramer and pentamer models in crystal. The inset structures show the molecular conformation in excited state.

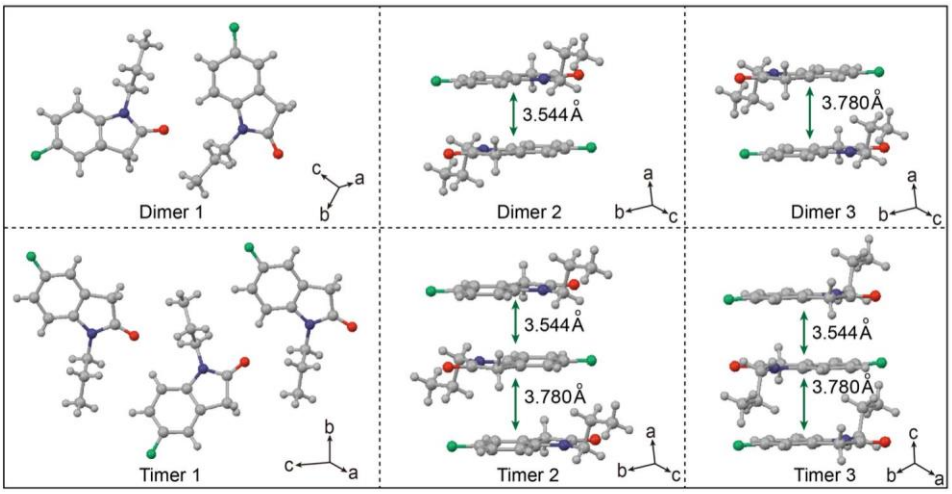

Supplementary Figure 40. Different types of dimer and trimer in FPO crystal.

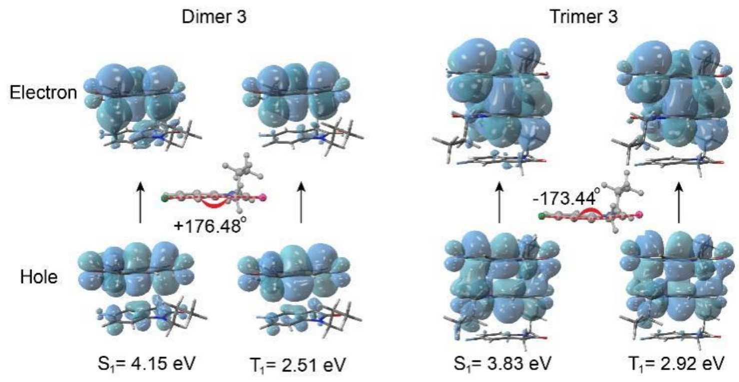

Supplementary Figure 41. NTOs contributing to the lowest-energy triplet transitions of FPO trimer 3 and dimer 3 models in crystal. The inset structures show the molecular conformation in excited state of dimer 3 (left) and trimer 3 (right) models.

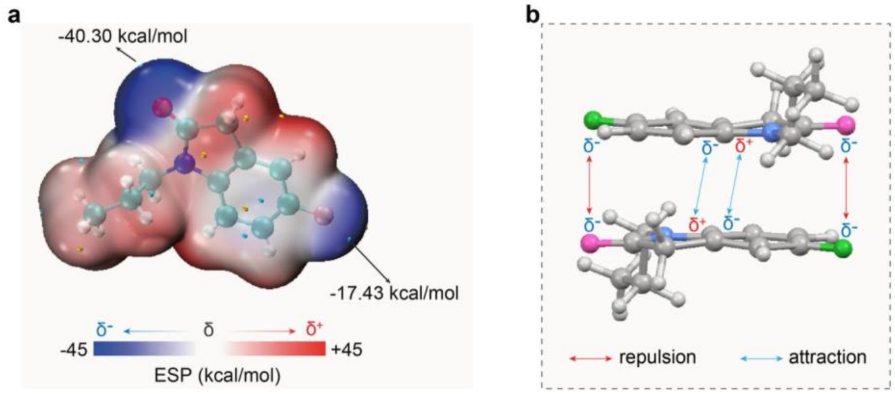

Supplementary Figure 42. Influence of electrostatic interaction on molecular deformation. a, Electrostatic potential (ESP) distribution for the FPO molecule in single molecular state. **b**, Proposed electrostatic interactions in a dimer model based on ESP distribution in single molecular state.

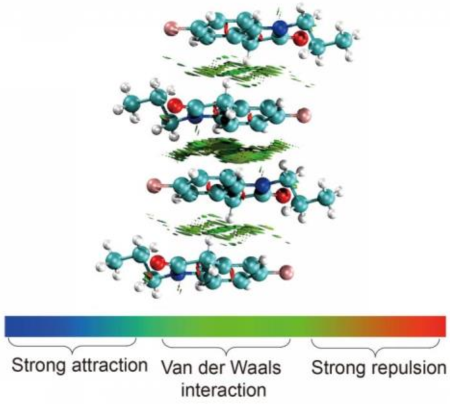

Supplementary Figure 43. Calculated intermolecular interactions (green iso-surface) in tetramer model of FPO crystal.

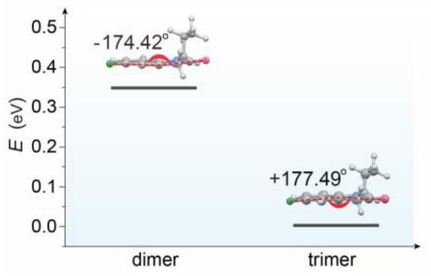

Supplementary Figure 44. Conformational energies estimated for the optimized structures of FPO molecule in dimer (left) and trimer (right) state.

V. Expansion of abnormal thermally-stimulated dynamic organic phosphors.

FMO, FEO, CEO and CPO phosphors showed similar solid state absorption spectra under ambient conditions, which show main absorption bands at around 300 nm. The solid-state phosphorescence efficiency of FMO, FEO, CEO and CPO phosphors are 3.3%, 6.5%, 2.9% and 6.7%, respectively.

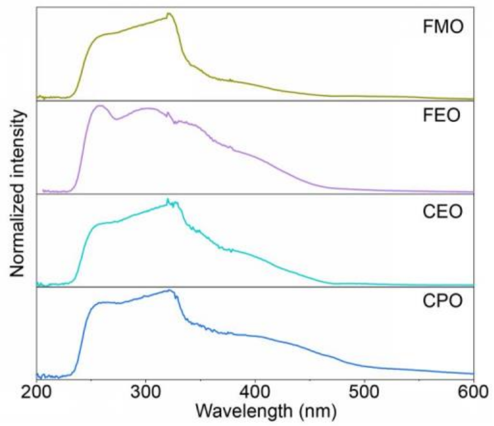

Supplementary Figure 45. Normalized UV absorption of FMO, FEO, CEO and CPO phosphors in crystal state under ambient conditions.

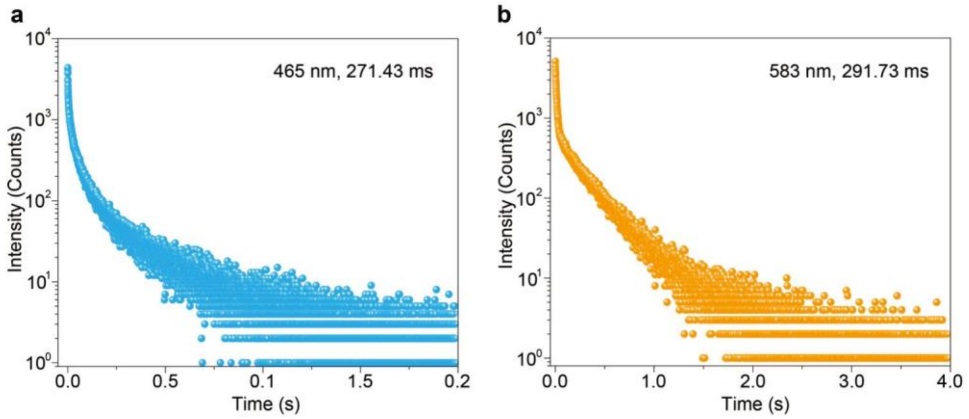

Supplementary Figure 46. Time resolved decay curves of the FMO phosphor monitoring at 465 (a) and 583 nm (b) under ambient conditions.

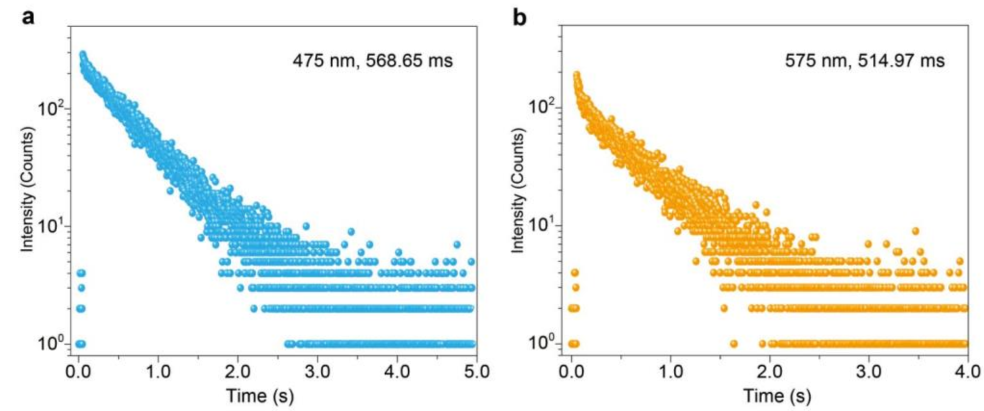

Supplementary Figure 47. Time resolved decay curves of the FEO phosphor monitoring at 475 (a) and 575 nm (b) under ambient conditions.

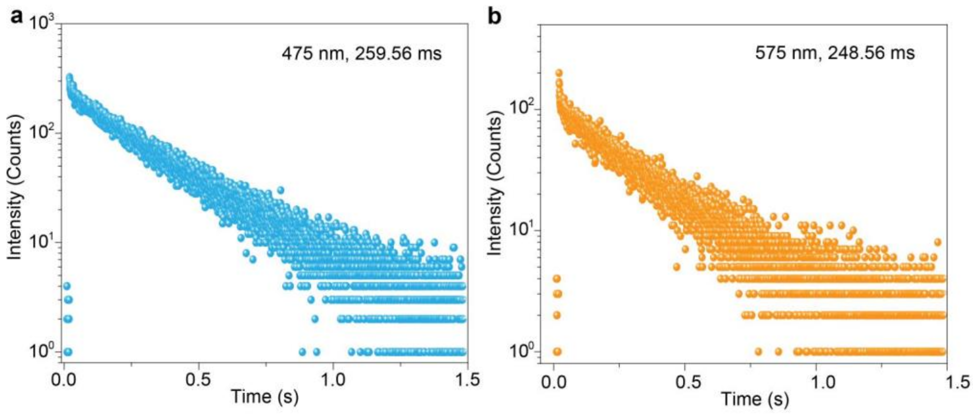

Supplementary Figure 48. Time resolved decay curves of the CEO phosphor monitoring at 475 (a) and 575 nm (b) under ambient conditions.

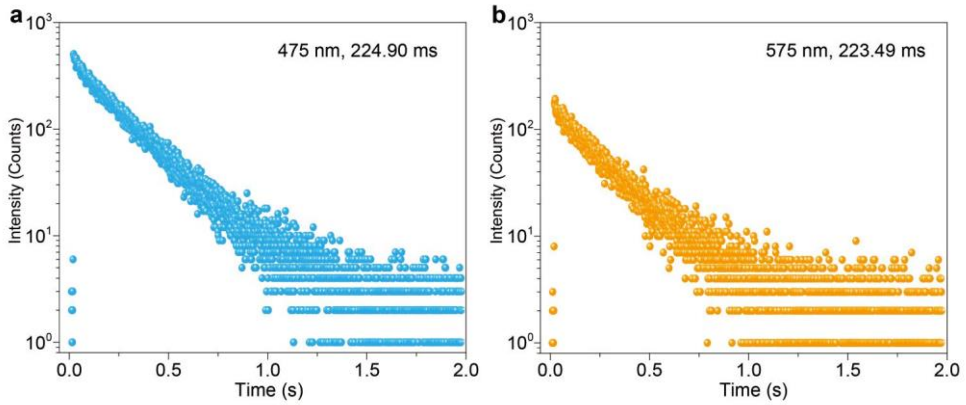

Supplementary Figure 49. Time resolved decay curves of the CPO phosphor monitoring at 475 (a) and 575 nm (b) under ambient conditions.

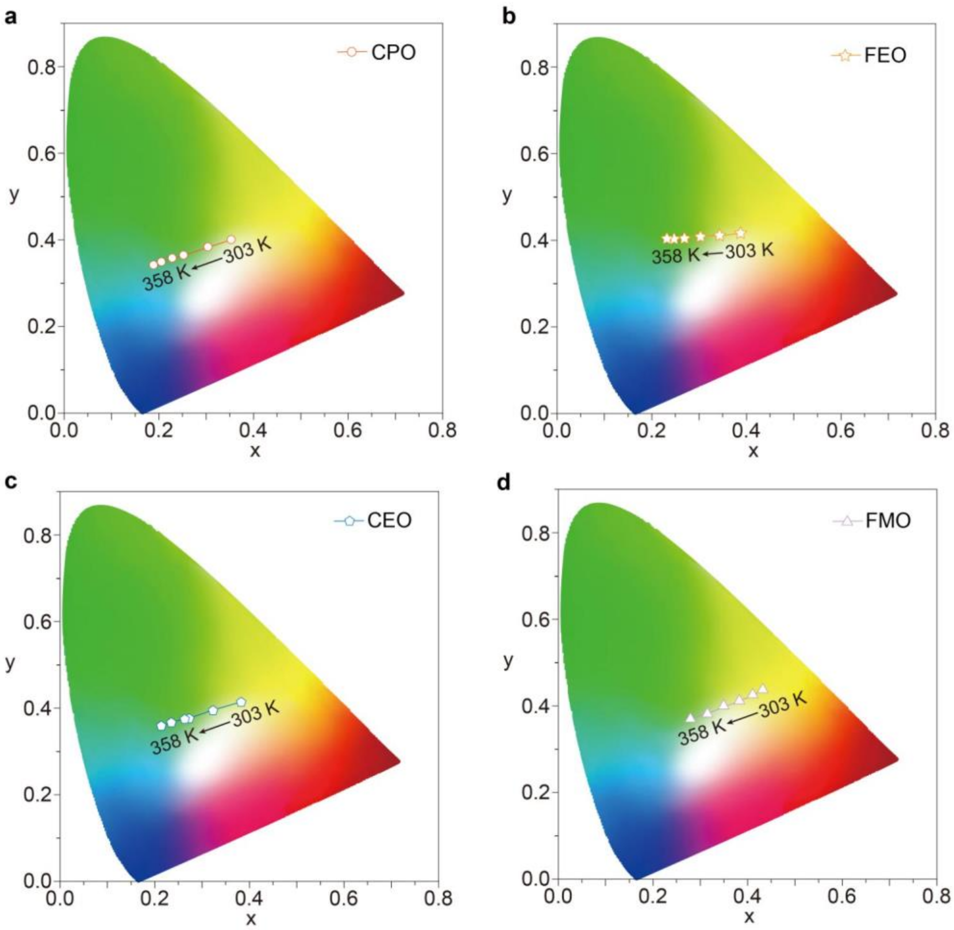

Supplementary Figure 50. A CIE chromaticity coordinate diagram of the phosphorescence for CPO (a), FEO (b), CEO (c) and FMO (d) phosphors as temperature change from 303 to 358 K.

Supplementary Table 2. Structure data of the FMO single crystal at 90, 198, 273, 303 and 333 K.

**Formula**			C_9H_8FNO		
**Temperature**	90 K	198 K	273 K	303 K	333 K
**Space Group**	P 2_1/n	P 2_1/n	P 2_1/n	P 2_1/n	P 2_1/n
**Cell Lengths (Å)**	a= 6.8376(2)<br>b= 8.1570(2)<br>c=13.5543(3)	a= 6.8940(3)<br>b= 8.1788(3)<br>c= 13.6505(6)	a=6.9518(17)<br>b=8.193(2)<br>c=13.703(3)	a=6.981(3)<br>b=8.220(4)<br>c=13.751(7)	a=7.0090(15)<br>b=8.2168(18)<br>c=13.769(3)
**Cell Angles (o)**	α=90<br>β=102.904(2)<br>γ=90	α=90<br>β= 103.088(4)<br>γ=90	α=90<br>β=103.024(7)<br>γ=90	α=90<br>β=102.928(16)<br>γ=90	α=90<br>β=103.093(7)<br>γ=90
**Cell Volume ($Å^3$)**	736.89	749.685	760.392	769.083	772.364
**Z, Z'**	Z:4; Z':0	Z:4; Z':0	Z:1; Z':0	Z: 8; Z': 0	Z: 4; Z': 0
**Density (g/cm^3)**	1.489	1.463	1.443	1.426	1.4203
**CCDC number**	2283869	2283870	2071008	2071009	2071059

Supplementary Table 3. Structure data of the FEO and CEO single crystals.

Name	FEO	CEO
**Formula**	$C_{10}H_{10}FNO$	$C_{10}H_{10}ClNO$
**Space Group**	P 2_1/c	P 2_1/c
**Cell Lengths (Å)**	a=7.686(5)<br>b=10.002(6)<br>c=12.491(8)	a=7.711(2)<br>b=10.551(3)<br>c=12.429(3)
**Cell Angles (o)**	α=90<br>β=105.946(14)<br>γ=90	α=90<br>β=105.507(9)<br>γ=90
**Cell Volume (Å^3)**	923.301	974.398
**Z, Z'**	Z: 4; Z': 0	Z: 4; Z': 0
**Density (g/cm^3)**	1.289	1.334
**CCDC number**	2071411	2116228

Supplementary Table 4 Structure data of the CPO single crystal at 273, 293, 313, 333 and 353 K.

**Formula**			$C_{11}H_{12}ClNO$		
**Temperature**	273 K	293 K	313 K	333 K	353 K
**Space Group**	P 2_1/c	P 2_1/c	P 2_1/c	P 2_1/c	P 2_1/c
**Cell Lengths (Å)**	a=7.6768(3)<br>b=11.3464(4)<br>c=12.2878(4)	a=7.6953(3)<br>b=11.3523(4)<br>c=12.3087(4)	a=7.7148(3)<br>b=11.3567(4)<br>c=12.3313(4)	a=7.7378(3)<br>b=11.3606(4)<br>c=12.3567(5)	a=7.7631(4)<br>b=11.3632(5)<br>c=12.3848(5)
**Cell Angles (o)**	α=90<br>β=106.6120<br>(10)<br>γ=90	α=90<br>β=106.7090<br>(10)<br>γ=90	α=90<br>β=106.8040<br>(10)<br>γ=90	α=90<br>β=106.9000<br>(10)<br>γ=90	α=90<br>β=106.9920<br>(10)<br>γ=90
**Cell Volume (Å^3)**	1025.64	1029.88	1034.27	1039.32	1044.82
**Z, Z'**	Z:4; Z':0	Z:4; Z':0	Z: 4; Z': 0	Z: 4; Z': 0	Z: 4; Z': 0
**Density (g/cm^3)**	1.358	1.352	1.346	1.340	1.333
**CCDC number**	2116229	2116230	2116231	2116232	2116233

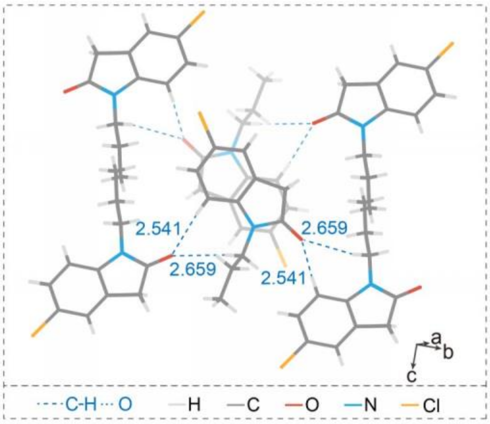

Supplementary Figure 51. Intermolecular interactions of CPO crystal at 293 K.

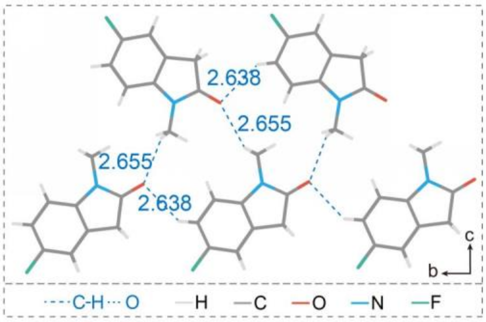

Supplementary Figure 52. Intermolecular interactions of FMO crystal at 303 K.

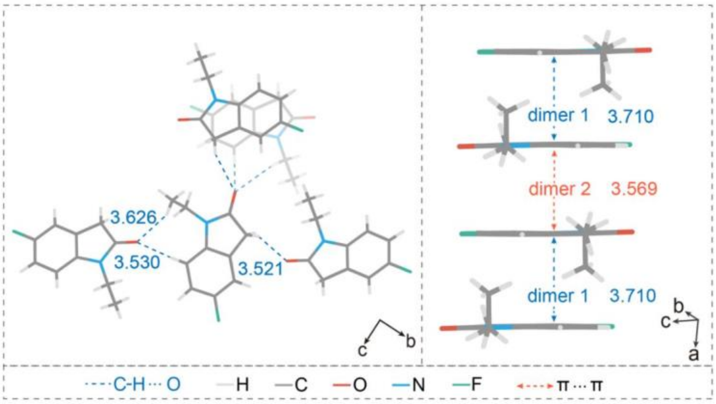

Supplementary Figure 53. Intermolecular interactions (left) and molecular arrangement (right) of FEO crystal at 297 K.

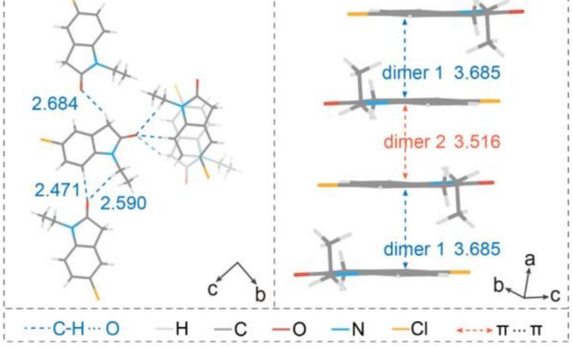

Supplementary Figure 54. Intermolecular interactions (left) and molecular arrangement (right) of CEO crystal at 242 K.

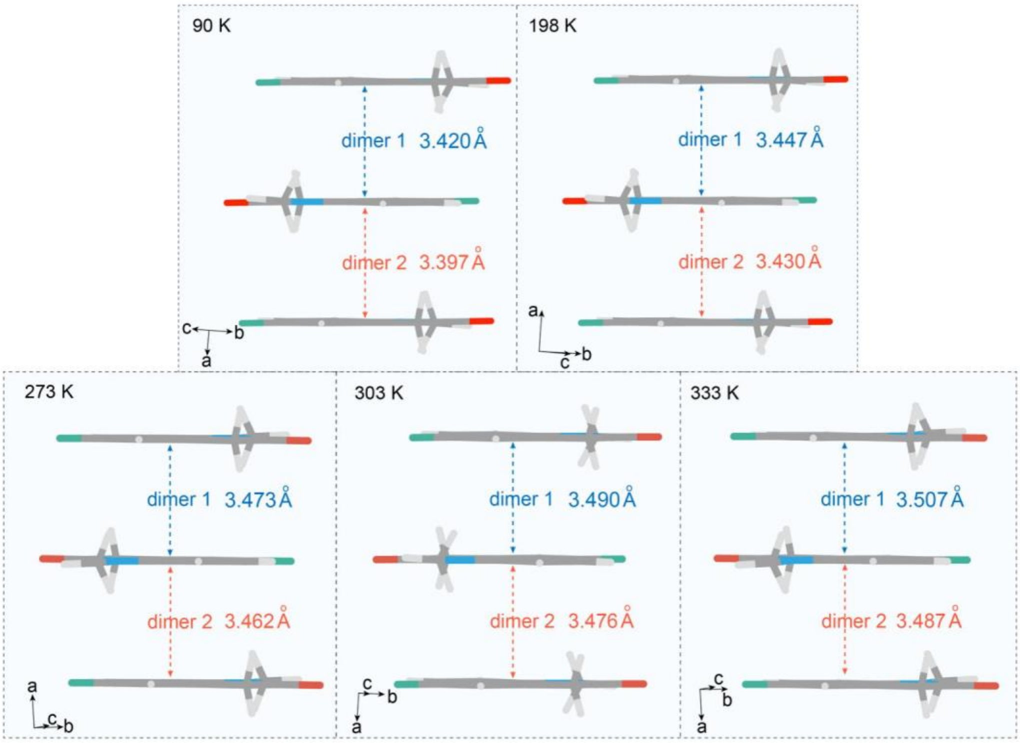

Supplementary Figure 55. Intermolecular π-π stacking distances in FMO crystal at different temperatures (90, 198, 273, 303 and 333 K).

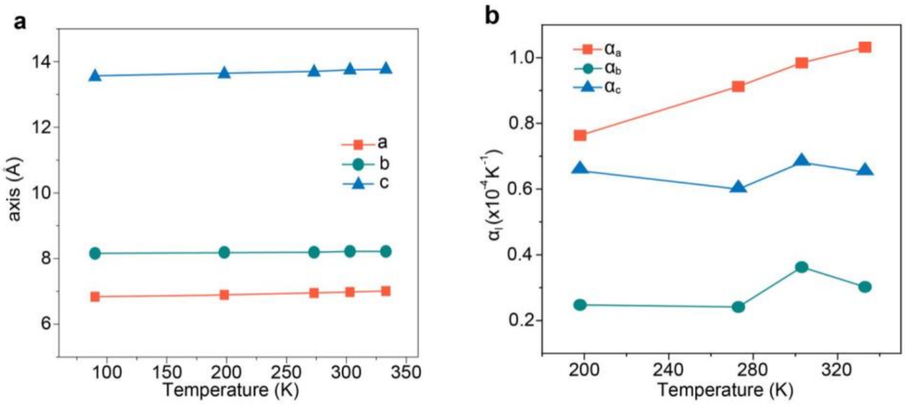

Supplementary Figure 56. Effect of temperature on structural parameters of the FMO crystal. a, Edge variation along the crystallographic **a**, **b** and **c** axis. **b,** Thermal expansion coefficient along the principal axes (**a**, **b** and **c**).

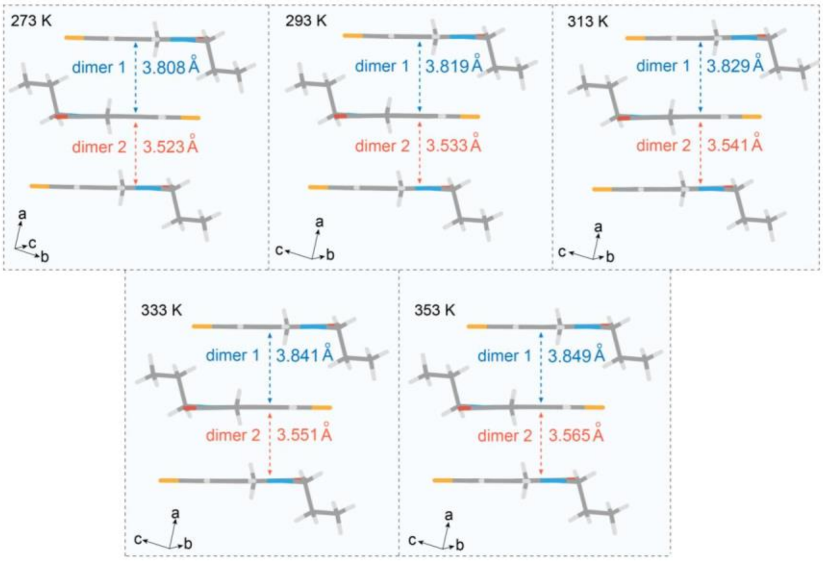

Supplementary Figure 57. Distances between neighboring molecules by π-π stacking in CPO crystal at different temperatures (273, 293, 313, 333 and 353 K).

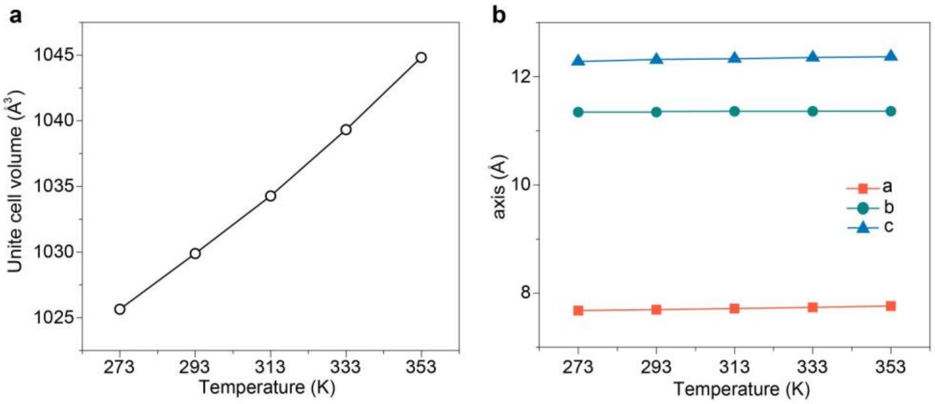

Supplementary Figure 58. Effect of temperature on structural parameters of the CPO crystal. a, Variation of cell volume. **b,** Edge variation along the crystallographic **a**, **b** and **c** axis.

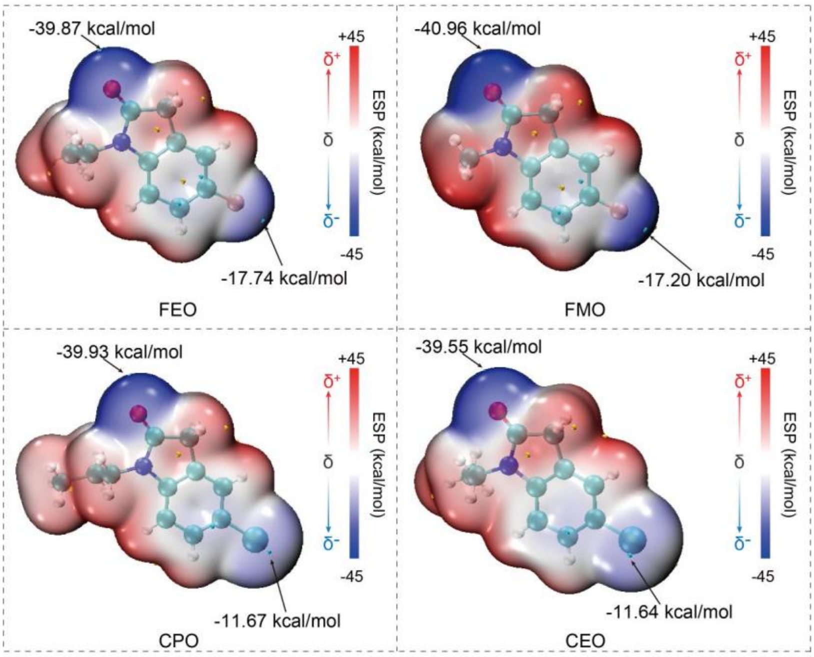

Supplementary Figure 59. ESP distribution of FEO, FMO, CPO and CEO in single molecular state.

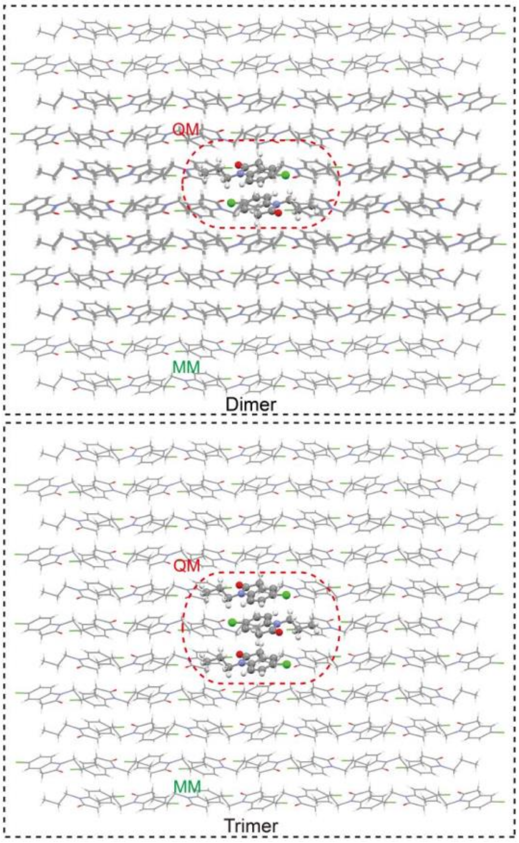

Supplementary Figure 60. Setup of QM/MM model for CPO phosphor.

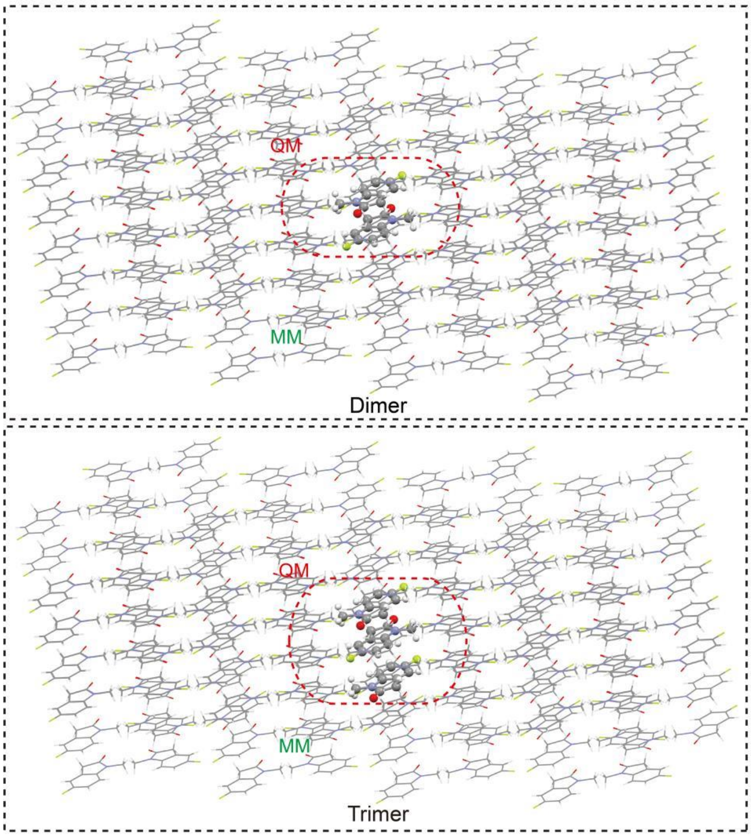

Supplementary Figure 61. Setup of QM/MM model for FMO phosphor.

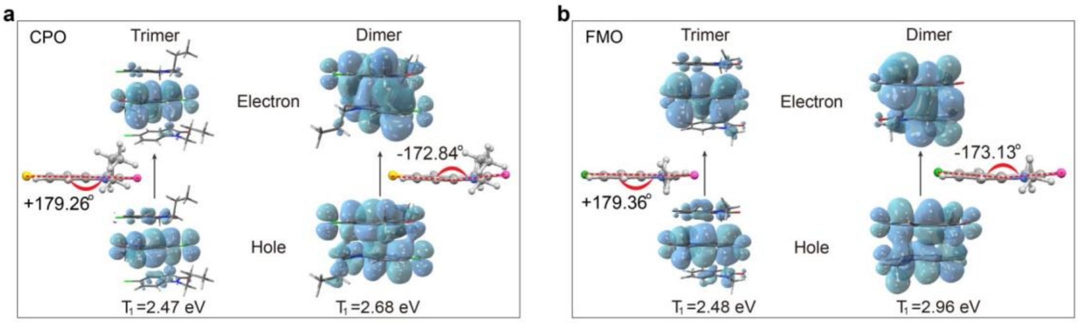

Supplementary Figure 62. NTOs contributing to the lowest-energy triplet transitions of trimer and dimer models for CPO (a) and FMO (b) in crystal. The inset structures show the molecular conformation in excited state.

VI. Applications of thermally-stimulated dynamic organic phosphors

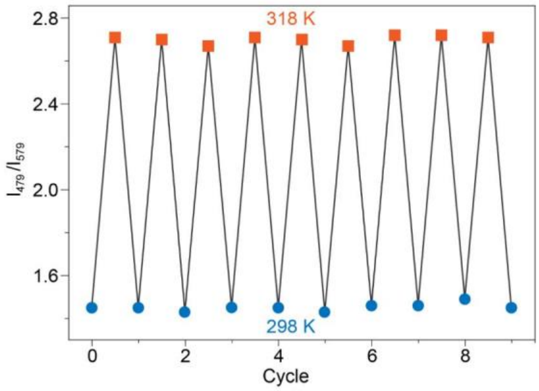

Supplementary Figure 63. Reversible dynamic phosphorescence by regulating temperature between 298 and 318 K.

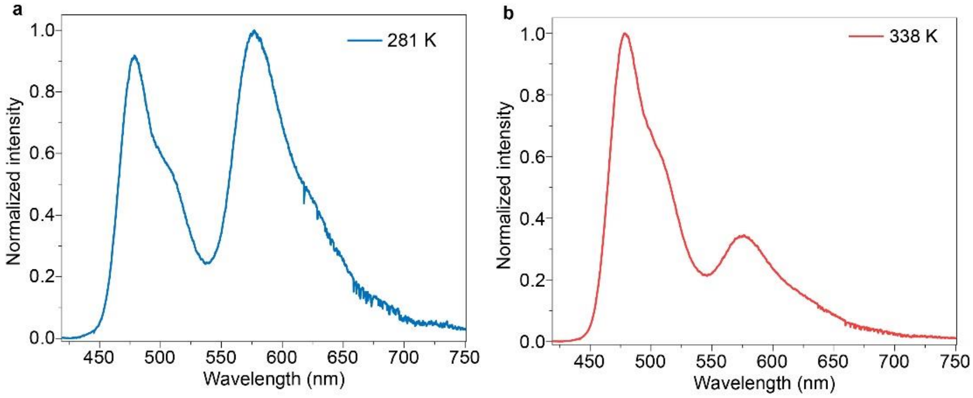

Supplementary Figure 64. Phosphorescence spectra of the grinding FPO powder at 281 K (a) and 338 K (b).

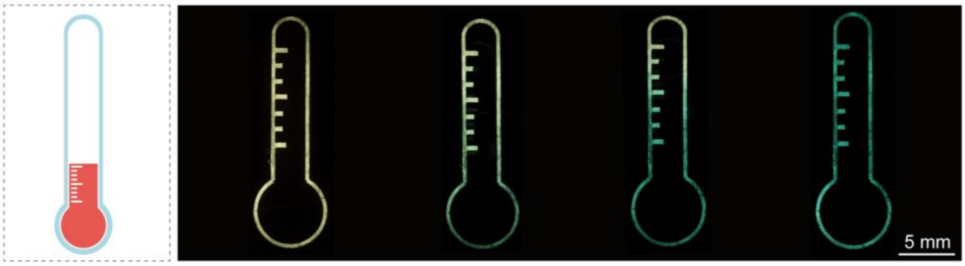

Supplementary Figure 65. Demonstration of the FPO phosphor for temperature sensing application. Note that the temperature rises from left to right.

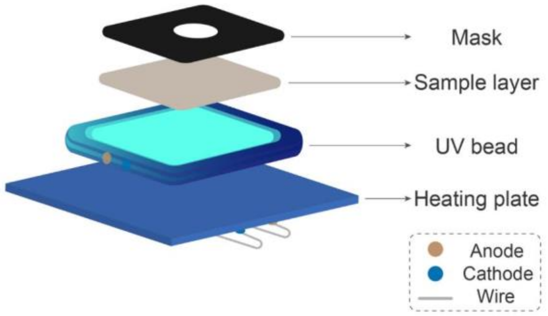

Supplementary Figure 66. A schematic of a prototype device for afterglow display.

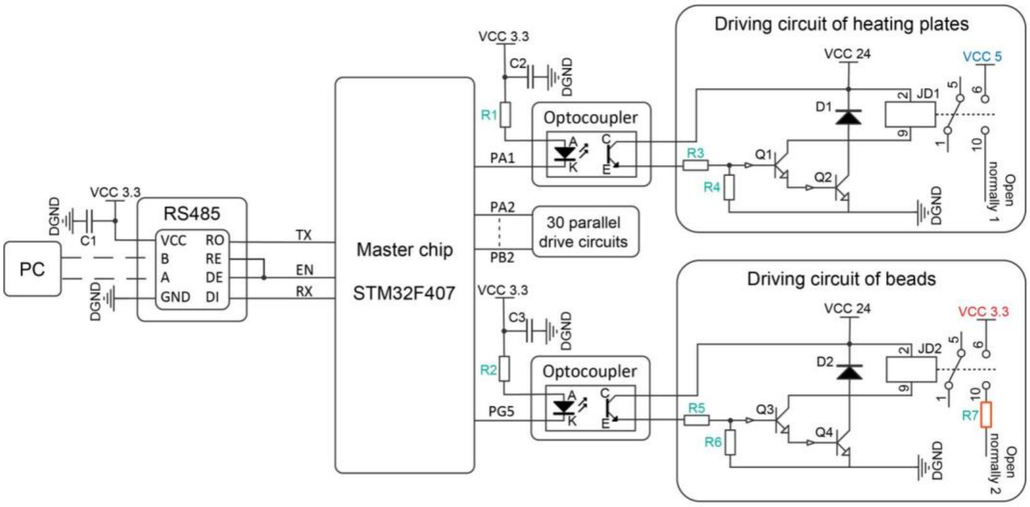

Supplementary Figure 67. Circuit schematic for colorful afterglow display. Note that RS485 and master chip together form a microcontroller. VCC 3.3, VCC 24 and VCC 5 represent the input voltage after conversion by the voltage converter. Optocouplers and driving circuits of heating plates and beads together constitute relay control boards.

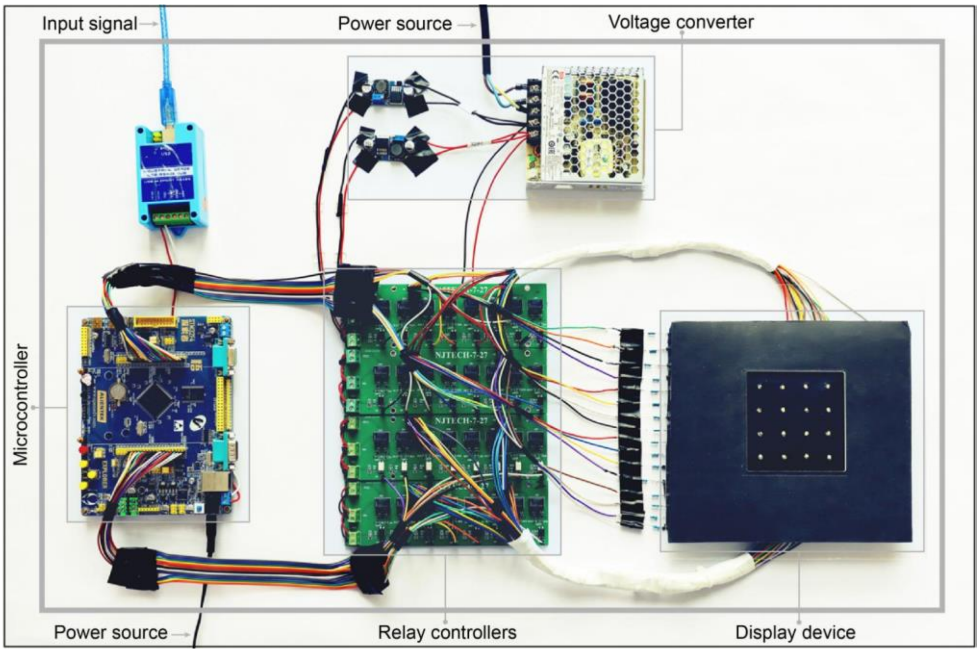

Supplementary Figure 68. Photograph of colorful afterglow display device.

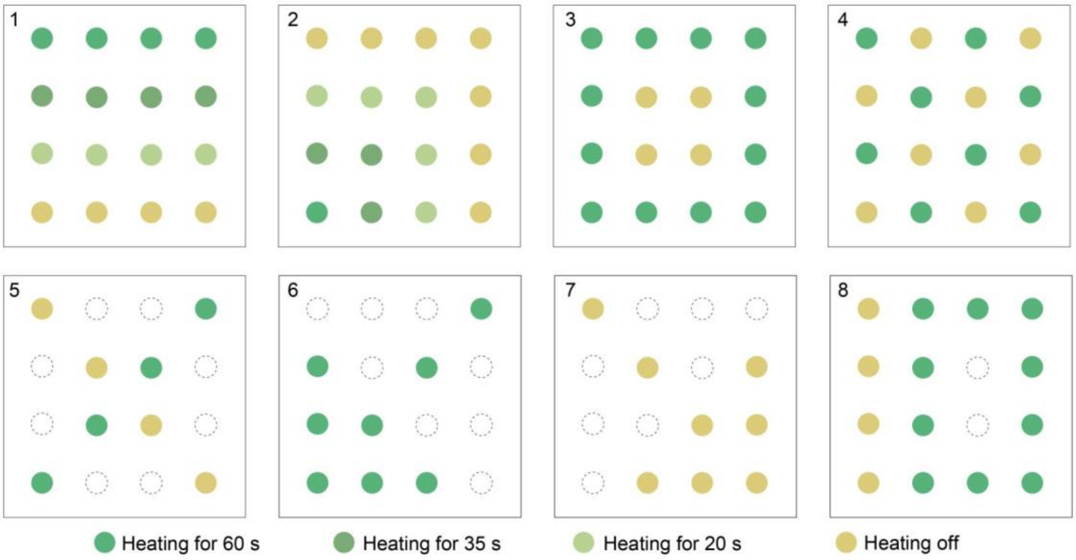

Supplementary Figure 69. Schematic of afterglow patterns with the colorful afterglow display.

Supplementary Table 5. Program codes for colorful afterglow display.

```
#include "logic.h"

#include "malloc.h"

#include "delay.h"

#include "led.h"

void modbus_send_open(m_frame_typedef * fx, u8 function)

{

u8 i;

fx->address = 0X02;

fx->function = function;

fx->datalen = 2;

fx->data=mymalloc(SRAMIN,fx->datalen);

for(i=0;i<1;i++)

{

fx->data[i] =0;

fx->data[i+1] = 0;

}
```

```
for(i=0;i<1;i++)

{

mb_packsend_frame(fx);

delay_ms(30);

}

myfree(SRAMIN,fx->data);}

void modbus_send_close(m_frame_typedef * fx, u8 function)

{

u8 i;

fx->address = 0X02;

fx->function = function;

fx->datalen = 2;

fx->data=mymalloc(SRAMIN,fx->datalen);

for(i=0;i<1;i++)

{

fx->data[i] =0;

fx->data[i+1] = 1;}

for(i=0;i<1;i++)

{

mb_packsend_frame(fx);

delay_ms(30);}

myfree(SRAMIN,fx->data);

}

/* receive instructions;

Detection pool 1 performs related control */

void modbus_action(m_frame_typedef * fx)
```

```
{
m_frame_typedef rxframe;
if(fx->address == 0X01 )
{
/* The total pattern instruction code */
modbus_send_open(&rxframe, 0x0D);
/* the first pattern control path */
/*    0-25 s    */
GPIO_ResetBits(GPIOF,GPIO_Pin_0|GPIO_Pin_1|GPIO_Pin_2|GPIO_Pin_3);
GPIO_SetBits(GPIOF,GPIO_Pin_4|GPIO_Pin_5|GPIO_Pin_6|GPIO_Pin_7);
GPIO_SetBits(GPIOE,GPIO_Pin_0|GPIO_Pin_1|GPIO_Pin_5|GPIO_Pin_6);
GPIO_SetBits(GPIOD,GPIO_Pin_11);
GPIO_SetBits(GPIOC,GPIO_Pin_13|GPIO_Pin_1|GPIO_Pin_2);
GPIO_SetBits(GPIOD,GPIO_Pin_2|GPIO_Pin_3|GPIO_Pin_4|GPIO_Pin_5|GPIO_Pin_6|GPIO_Pin_7);
GPIO_SetBits(GPIOG,GPIO_Pin_2|GPIO_Pin_3|GPIO_Pin_4|GPIO_Pin_5|GPIO_Pin_6|GPIO_Pin_7);
GPIO_SetBits(GPIOB,GPIO_Pin_4|GPIO_Pin_5|GPIO_Pin_6|GPIO_Pin_7);
delay_ms(25000);
/*    25-40 s    */
GPIO_ResetBits(GPIOF,GPIO_Pin_4|GPIO_Pin_5|GPIO_Pin_6|GPIO_Pin_7);
delay_ms(15000);
/*    40-50 s    */
GPIO_ResetBits(GPIOE,GPIO_Pin_0|GPIO_Pin_1);
GPIO_ResetBits(GPIOC,GPIO_Pin_1|GPIO_Pin_2);
delay_ms(10000);
/*    51-60 s    */
GPIO_ResetBits(GPIOD,GPIO_Pin_2|GPIO_Pin_3|GPIO_Pin_4|GPIO_Pin_5|GPIO_Pin_6|GPIO_Pin_7);
```

```
GPIO_ResetBits(GPIOG,GPIO_Pin_2|GPIO_Pin_3|GPIO_Pin_4|GPIO_Pin_5|GPIO_Pin_6|GPIO_Pin_7);

GPIO_ResetBits(GPIOB,GPIO_Pin_4|GPIO_Pin_5|GPIO_Pin_6|GPIO_Pin_7);

delay_ms(10000);

/*    61-80 s    */

GPIO_SetBits(GPIOF,GPIO_Pin_0|GPIO_Pin_1|GPIO_Pin_2|GPIO_Pin_3|GPIO_Pin_4|GPIO_Pin_5|GPIO_
Pin_6|GPIO_Pin_7);

GPIO_SetBits(GPIOE,GPIO_Pin_0|GPIO_Pin_1);

GPIO_SetBits(GPIOC,GPIO_Pin_1|GPIO_Pin_2);

GPIO_SetBits(GPIOD,GPIO_Pin_2|GPIO_Pin_3|GPIO_Pin_4|GPIO_Pin_5|GPIO_Pin_6|GPIO_Pin_7);

GPIO_SetBits(GPIOG,GPIO_Pin_2|GPIO_Pin_3|GPIO_Pin_4|GPIO_Pin_5|GPIO_Pin_6|GPIO_Pin_7);

GPIO_SetBits(GPIOB,GPIO_Pin_4|GPIO_Pin_5|GPIO_Pin_6|GPIO_Pin_7);

delay_ms(20000);

delay_ms(60000);

delay_ms(60000);

/****    the second pattern control path    ****/

/*    0-25 s    */

GPIO_SetBits(GPIOF,GPIO_Pin_0|GPIO_Pin_1|GPIO_Pin_2|GPIO_Pin_3|GPIO_Pin_4|GPIO_Pin_5|GPIO_
Pin_6|GPIO_Pin_7);

GPIO_SetBits(GPIOC,GPIO_Pin_13|GPIO_Pin_2|GPIO_Pin_1|GPIO_Pin_3);

GPIO_SetBits(GPIOE,GPIO_Pin_0|GPIO_Pin_1|GPIO_Pin_5|GPIO_Pin_6);

GPIO_ResetBits(GPIOD,GPIO_Pin_11);

GPIO_SetBits(GPIOD,GPIO_Pin_2|GPIO_Pin_3|GPIO_Pin_4|GPIO_Pin_5|GPIO_Pin_6|GPIO_Pin_7);

GPIO_SetBits(GPIOG,GPIO_Pin_2|GPIO_Pin_3|GPIO_Pin_4|GPIO_Pin_5|GPIO_Pin_6|GPIO_Pin_7);

GPIO_SetBits(GPIOB,GPIO_Pin_4|GPIO_Pin_5|GPIO_Pin_6|GPIO_Pin_7);

delay_ms(25000);

/*    25-40 s    */

GPIO_ResetBits(GPIOE,GPIO_Pin_0|GPIO_Pin_1|GPIO_Pin_5);
```

```
delay_ms(15000);

/*    40-50 s    */

GPIO_ResetBits(GPIOF,GPIO_Pin_4|GPIO_Pin_5|GPIO_Pin_6);

GPIO_ResetBits(GPIOC,GPIO_Pin_1);

GPIO_ResetBits(GPIOE,GPIO_Pin_6);

delay_ms(10000);

/*    51-60 s    */

GPIO_ResetBits(GPIOC,GPIO_Pin_3);

GPIO_ResetBits(GPIOD,GPIO_Pin_2|GPIO_Pin_3|GPIO_Pin_4|GPIO_Pin_5|GPIO_Pin_6|GPIO_Pin_7);

GPIO_ResetBits(GPIOG,GPIO_Pin_2|GPIO_Pin_3|GPIO_Pin_4|GPIO_Pin_5|GPIO_Pin_6|GPIO_Pin_7);

GPIO_ResetBits(GPIOB,GPIO_Pin_4|GPIO_Pin_5|GPIO_Pin_6|GPIO_Pin_7);

delay_ms(10000);

/*    61-80 s    */

GPIO_SetBits(GPIOF,GPIO_Pin_4|GPIO_Pin_5|GPIO_Pin_6);

GPIO_SetBits(GPIOE,GPIO_Pin_0|GPIO_Pin_1|GPIO_Pin_5|GPIO_Pin_6);

GPIO_SetBits(GPIOC,GPIO_Pin_3|GPIO_Pin_1);

GPIO_SetBits(GPIOD,GPIO_Pin_11);

GPIO_SetBits(GPIOD,GPIO_Pin_2|GPIO_Pin_3|GPIO_Pin_4|GPIO_Pin_5|GPIO_Pin_6|GPIO_Pin_7);

GPIO_SetBits(GPIOG,GPIO_Pin_2|GPIO_Pin_3|GPIO_Pin_4|GPIO_Pin_5|GPIO_Pin_6|GPIO_Pin_7);

GPIO_SetBits(GPIOB,GPIO_Pin_4|GPIO_Pin_5|GPIO_Pin_6|GPIO_Pin_7);

delay_ms(20000);

delay_ms(60000);

delay_ms(60000);

/****    the third pattern control path    ****/

/*    0-50 s    */

GPIO_ResetBits(GPIOF,GPIO_Pin_0|GPIO_Pin_1|GPIO_Pin_2|GPIO_Pin_3|GPIO_Pin_4|GPIO_Pin_7);
```

```
GPIO_ResetBits(GPIOE,GPIO_Pin_0|GPIO_Pin_5|GPIO_Pin_6);

GPIO_ResetBits(GPIOC,GPIO_Pin_2|GPIO_Pin_13);

GPIO_ResetBits(GPIOD,GPIO_Pin_11);

GPIO_SetBits(GPIOF,GPIO_Pin_5|GPIO_Pin_6);

GPIO_SetBits(GPIOC,GPIO_Pin_1|GPIO_Pin_3);

GPIO_SetBits(GPIOE,GPIO_Pin_1);

GPIO_SetBits(GPIOD,GPIO_Pin_2|GPIO_Pin_3|GPIO_Pin_4|GPIO_Pin_5|GPIO_Pin_6|GPIO_Pin_7);

GPIO_SetBits(GPIOG,GPIO_Pin_2|GPIO_Pin_3|GPIO_Pin_4|GPIO_Pin_5|GPIO_Pin_6|GPIO_Pin_7);

GPIO_SetBits(GPIOB,GPIO_Pin_4|GPIO_Pin_5|GPIO_Pin_6|GPIO_Pin_7);

delay_ms(50000);

/*    51-60 s    */

GPIO_ResetBits(GPIOC,GPIO_Pin_3);

GPIO_ResetBits(GPIOD,GPIO_Pin_2|GPIO_Pin_3|GPIO_Pin_4|GPIO_Pin_5|GPIO_Pin_6|GPIO_Pin_7);

GPIO_ResetBits(GPIOG,GPIO_Pin_2|GPIO_Pin_3|GPIO_Pin_4|GPIO_Pin_5|GPIO_Pin_6|GPIO_Pin_7);

GPIO_ResetBits(GPIOB,GPIO_Pin_4|GPIO_Pin_5|GPIO_Pin_6|GPIO_Pin_7);

delay_ms(10000);

/*    61-80 s    */

GPIO_SetBits(GPIOF,GPIO_Pin_0|GPIO_Pin_1|GPIO_Pin_2|GPIO_Pin_3|GPIO_Pin_4|GPIO_Pin_7);

GPIO_SetBits(GPIOE,GPIO_Pin_0|GPIO_Pin_5|GPIO_Pin_6);

GPIO_SetBits(GPIOD,GPIO_Pin_11);

GPIO_SetBits(GPIOC,GPIO_Pin_2|GPIO_Pin_3|GPIO_Pin_13);

GPIO_SetBits(GPIOD,GPIO_Pin_2|GPIO_Pin_3|GPIO_Pin_4|GPIO_Pin_5|GPIO_Pin_6|GPIO_Pin_7);

GPIO_SetBits(GPIOG,GPIO_Pin_2|GPIO_Pin_3|GPIO_Pin_4|GPIO_Pin_5|GPIO_Pin_6|GPIO_Pin_7);

GPIO_SetBits(GPIOB,GPIO_Pin_4|GPIO_Pin_5|GPIO_Pin_6|GPIO_Pin_7);

delay_ms(20000);

delay_ms(60000);
```

```
delay_ms(60000);

/****   the fourth pattern control path   ****/

/*   0-50 s   */

GPIO_ResetBits(GPIOF,GPIO_Pin_0|GPIO_Pin_2|GPIO_Pin_5|GPIO_Pin_7);

GPIO_ResetBits(GPIOE,GPIO_Pin_0|GPIO_Pin_5);

GPIO_ResetBits(GPIOC,GPIO_Pin_1|GPIO_Pin_13);

GPIO_SetBits(GPIOF,GPIO_Pin_1|GPIO_Pin_3|GPIO_Pin_4|GPIO_Pin_6);

GPIO_SetBits(GPIOC,GPIO_Pin_2|GPIO_Pin_3);

GPIO_SetBits(GPIOE,GPIO_Pin_1|GPIO_Pin_6);

GPIO_SetBits(GPIOD,GPIO_Pin_11);

GPIO_SetBits(GPIOD,GPIO_Pin_2|GPIO_Pin_3|GPIO_Pin_4|GPIO_Pin_5|GPIO_Pin_6|GPIO_Pin_7);

GPIO_SetBits(GPIOG,GPIO_Pin_2|GPIO_Pin_3|GPIO_Pin_4|GPIO_Pin_5|GPIO_Pin_6|GPIO_Pin_7);

GPIO_SetBits(GPIOB,GPIO_Pin_4|GPIO_Pin_5|GPIO_Pin_6|GPIO_Pin_7);

delay_ms(50000);

/*   51-60 s   */

GPIO_ResetBits(GPIOC,GPIO_Pin_3);

GPIO_ResetBits(GPIOD,GPIO_Pin_2|GPIO_Pin_3|GPIO_Pin_4|GPIO_Pin_5|GPIO_Pin_6|GPIO_Pin_7);

GPIO_ResetBits(GPIOG,GPIO_Pin_2|GPIO_Pin_3|GPIO_Pin_4|GPIO_Pin_5|GPIO_Pin_6|GPIO_Pin_7);

GPIO_ResetBits(GPIOB,GPIO_Pin_4|GPIO_Pin_5|GPIO_Pin_6|GPIO_Pin_7);

delay_ms(10000);

/*   61-80 s   */

GPIO_SetBits(GPIOF,GPIO_Pin_0|GPIO_Pin_2|GPIO_Pin_5|GPIO_Pin_7);

GPIO_SetBits(GPIOE,GPIO_Pin_0|GPIO_Pin_5);

GPIO_SetBits(GPIOC,GPIO_Pin_1|GPIO_Pin_3|GPIO_Pin_13);

GPIO_SetBits(GPIOD,GPIO_Pin_2|GPIO_Pin_3|GPIO_Pin_4|GPIO_Pin_5|GPIO_Pin_6|GPIO_Pin_7);

GPIO_SetBits(GPIOG,GPIO_Pin_2|GPIO_Pin_3|GPIO_Pin_4|GPIO_Pin_5|GPIO_Pin_6|GPIO_Pin_7);
```

```
GPIO_SetBits(GPIOB,GPIO_Pin_4|GPIO_Pin_5|GPIO_Pin_6|GPIO_Pin_7);

delay_ms(20000);

delay_ms(60000);

delay_ms(60000);

/****    the fifth pattern control path    ****/

/*    0-50 s    */

GPIO_SetBits(GPIOF,GPIO_Pin_0|GPIO_Pin_1|GPIO_Pin_2|GPIO_Pin_4|GPIO_Pin_5|GPIO_Pin_7);

GPIO_SetBits(GPIOE,GPIO_Pin_0|GPIO_Pin_5|GPIO_Pin_6);

GPIO_SetBits(GPIOC,GPIO_Pin_1|GPIO_Pin_2|GPIO_Pin_13);

GPIO_ResetBits(GPIOF,GPIO_Pin_3|GPIO_Pin_6);

GPIO_ResetBits(GPIOE,GPIO_Pin_1);

GPIO_ResetBits(GPIOD,GPIO_Pin_11);

GPIO_SetBits(GPIOD,GPIO_Pin_2|GPIO_Pin_3|GPIO_Pin_4|GPIO_Pin_5|GPIO_Pin_6|GPIO_Pin_7); //lamps off

GPIO_SetBits(GPIOG,GPIO_Pin_2|GPIO_Pin_3|GPIO_Pin_4|GPIO_Pin_5|GPIO_Pin_6|GPIO_Pin_7);

GPIO_SetBits(GPIOB,GPIO_Pin_4|GPIO_Pin_5|GPIO_Pin_6|GPIO_Pin_7);

delay_ms(50000);

/*    51-60 s    */

GPIO_ResetBits(GPIOD,GPIO_Pin_2|GPIO_Pin_5|GPIO_Pin_7); //lamps on

GPIO_ResetBits(GPIOG,GPIO_Pin_2|GPIO_Pin_5|GPIO_Pin_6);

GPIO_ResetBits(GPIOB,GPIO_Pin_4|GPIO_Pin_7);

delay_ms(10000);

/*    61-80 s    */

GPIO_SetBits(GPIOF,GPIO_Pin_3|GPIO_Pin_6);

GPIO_SetBits(GPIOE,GPIO_Pin_1);

GPIO_SetBits(GPIOD,GPIO_Pin_11);

GPIO_SetBits(GPIOD,GPIO_Pin_2|GPIO_Pin_5|GPIO_Pin_7); //lamp off
```

```
GPIO_SetBits(GPIOG,GPIO_Pin_2|GPIO_Pin_5|GPIO_Pin_6);

GPIO_SetBits(GPIOB,GPIO_Pin_4|GPIO_Pin_7);

delay_ms(20000);

delay_ms(60000);

delay_ms(60000);

/****    the sixth pattern control path    ****/

/*    0-50 s    */

GPIO_SetBits(GPIOF,GPIO_Pin_0|GPIO_Pin_1|GPIO_Pin_2|GPIO_Pin_5|GPIO_Pin_7);

GPIO_SetBits(GPIOC,GPIO_Pin_13|GPIO_Pin_1|GPIO_Pin_2);

GPIO_ResetBits(GPIOF,GPIO_Pin_3|GPIO_Pin_4|GPIO_Pin_6);

GPIO_ResetBits(GPIOE,GPIO_Pin_0|GPIO_Pin_1|GPIO_Pin_5|GPIO_Pin_6);

GPIO_ResetBits(GPIOD,GPIO_Pin_11);

GPIO_SetBits(GPIOD,GPIO_Pin_2|GPIO_Pin_3|GPIO_Pin_4|GPIO_Pin_5|GPIO_Pin_6|GPIO_Pin_7); // lamps
off

GPIO_SetBits(GPIOG,GPIO_Pin_2|GPIO_Pin_3|GPIO_Pin_4|GPIO_Pin_5|GPIO_Pin_6|GPIO_Pin_7);

GPIO_SetBits(GPIOB,GPIO_Pin_4|GPIO_Pin_5|GPIO_Pin_6|GPIO_Pin_7);

delay_ms(50000);

/*    51-60 s    */

GPIO_ResetBits(GPIOD,GPIO_Pin_5|GPIO_Pin_6);

GPIO_ResetBits(GPIOG,GPIO_Pin_2|GPIO_Pin_4|GPIO_Pin_5);

GPIO_ResetBits(GPIOB,GPIO_Pin_4|GPIO_Pin_5|GPIO_Pin_6);

delay_ms(10000);

/*    61-80 s    */

GPIO_SetBits(GPIOF,GPIO_Pin_3|GPIO_Pin_4|GPIO_Pin_6);

GPIO_SetBits(GPIOE,GPIO_Pin_0|GPIO_Pin_1|GPIO_Pin_5|GPIO_Pin_6);

GPIO_SetBits(GPIOD,GPIO_Pin_5|GPIO_Pin_6|GPIO_Pin_11);

GPIO_SetBits(GPIOG,GPIO_Pin_2|GPIO_Pin_4|GPIO_Pin_5);
```

```
GPIO_SetBits(GPIOB,GPIO_Pin_4|GPIO_Pin_5|GPIO_Pin_6);

delay_ms(20000);

delay_ms(60000);

delay_ms(60000);

/****    the seventh pattern control path    ****/

/*    0-50 s    */

GPIO_SetBits(GPIOF,GPIO_Pin_0|GPIO_Pin_1|GPIO_Pin_2|GPIO_Pin_3|GPIO_Pin_4|GPIO_Pin_5|GPIO_
Pin_6|GPIO_Pin_7);

GPIO_SetBits(GPIOE,GPIO_Pin_0|GPIO_Pin_1|GPIO_Pin_5|GPIO_Pin_6);

GPIO_SetBits(GPIOC,GPIO_Pin_13|GPIO_Pin_1|GPIO_Pin_2);

GPIO_SetBits(GPIOD,GPIO_Pin_2|GPIO_Pin_3|GPIO_Pin_4|GPIO_Pin_5|GPIO_Pin_6|GPIO_Pin_7|GPIO_
Pin_11);

GPIO_SetBits(GPIOG,GPIO_Pin_2|GPIO_Pin_3|GPIO_Pin_4|GPIO_Pin_5|GPIO_Pin_6|GPIO_Pin_7);

GPIO_SetBits(GPIOB,GPIO_Pin_4|GPIO_Pin_5|GPIO_Pin_6|GPIO_Pin_7);

delay_ms(50000);

/*    51-60 s    */

GPIO_ResetBits(GPIOD,GPIO_Pin_2|GPIO_Pin_7);

GPIO_ResetBits(GPIOG,GPIO_Pin_3|GPIO_Pin_6|GPIO_Pin_7);

GPIO_ResetBits(GPIOB,GPIO_Pin_5|GPIO_Pin_6|GPIO_Pin_7);

delay_ms(10000);

/*    61-80 s    */

GPIO_SetBits(GPIOD,GPIO_Pin_2|GPIO_Pin_7);

GPIO_SetBits(GPIOG,GPIO_Pin_3|GPIO_Pin_6|GPIO_Pin_7);

GPIO_SetBits(GPIOB,GPIO_Pin_5|GPIO_Pin_6|GPIO_Pin_7);

delay_ms(20000);

delay_ms(60000);

delay_ms(60000);
```

```
/****    the eighth pattern control path    ****/

/*    0-50 s    */

GPIO_SetBits(GPIOF,GPIO_Pin_0|GPIO_Pin_4|GPIO_Pin_6);

GPIO_SetBits(GPIOE,GPIO_Pin_0);

GPIO_SetBits(GPIOC,GPIO_Pin_1);

GPIO_ResetBits(GPIOF,GPIO_Pin_1|GPIO_Pin_2|GPIO_Pin_3|GPIO_Pin_5|GPIO_Pin_7);

GPIO_ResetBits(GPIOE,GPIO_Pin_1|GPIO_Pin_5|GPIO_Pin_6);

GPIO_ResetBits(GPIOC,GPIO_Pin_13|GPIO_Pin_2);

GPIO_SetBits(GPIOD,GPIO_Pin_2|GPIO_Pin_3|GPIO_Pin_4|GPIO_Pin_5|GPIO_Pin_6|GPIO_Pin_7|GPIO_
Pin_11);

GPIO_SetBits(GPIOG,GPIO_Pin_2|GPIO_Pin_3|GPIO_Pin_4|GPIO_Pin_5|GPIO_Pin_6|GPIO_Pin_7);

GPIO_SetBits(GPIOB,GPIO_Pin_4|GPIO_Pin_5|GPIO_Pin_6|GPIO_Pin_7);

delay_ms(50000);

/*    51-60 s    */

GPIO_ResetBits(GPIOD,GPIO_Pin_2|GPIO_Pin_3|GPIO_Pin_4|GPIO_Pin_5|GPIO_Pin_6|GPIO_Pin_7);

GPIO_ResetBits(GPIOG,GPIO_Pin_3|GPIO_Pin_4|GPIO_Pin_5|GPIO_Pin_7);

GPIO_ResetBits(GPIOB,GPIO_Pin_4|GPIO_Pin_5|GPIO_Pin_6|GPIO_Pin_7);

delay_ms(10000);

/*    61-80 s    */

GPIO_SetBits(GPIOF,GPIO_Pin_1|GPIO_Pin_2|GPIO_Pin_3|GPIO_Pin_5|GPIO_Pin_7);

GPIO_SetBits(GPIOE,GPIO_Pin_1|GPIO_Pin_5|GPIO_Pin_6);

GPIO_SetBits(GPIOC,GPIO_Pin_13|GPIO_Pin_2);

GPIO_SetBits(GPIOD,GPIO_Pin_2|GPIO_Pin_3|GPIO_Pin_4|GPIO_Pin_5|GPIO_Pin_6|GPIO_Pin_7);

GPIO_SetBits(GPIOG,GPIO_Pin_3|GPIO_Pin_4|GPIO_Pin_5|GPIO_Pin_7);

GPIO_SetBits(GPIOB,GPIO_Pin_4|GPIO_Pin_5|GPIO_Pin_6|GPIO_Pin_7);

delay_ms(20000);

}
```

```
}

}
```

VII. Supplementary movies

The supplementary movies were recorded in the dark with a camera (Canon EOS 700D).

Supplementary Movie 1. To present temperature dependent colorful phosphorescence, FPO crystals were heated on a hot stage. As temperature increases from 293 to 343 K, the afterglow color was tuned from yellow to cyan-blue.

Supplementary Movie 2. Under the guidance of microcontroller, various patterns were displayed in succession. Notably, in addition to microcontroller control, the patterns also show dynamic changes at different times.

VIII. References

1. Al-Attar, H. & Monkman, A. Room-temperature phosphorescence from films of isolated water soluble conjugated polymers in hydrogen-bonded matrices. *Adv. Funct. Mater.* **22**, 3824-3832 (2012).
2. An, Z. et al. Stabilizing triplet excited states for ultralong organic phosphorescence. *Nat. Mater.* **14**, 685-690 (2015).
3. Bhatia, H., Bhattacharjee, I. & Ray, D. Biluminescence via fluorescence and persistent phosphorescence in amorphous organic donor(d4)-acceptor(a) conjugates and application in data security protection. *J. Phys. Chem. Lett.* **9**, 3808-3813 (2018).
4. Bhattacharjee, I., Acharya, N., Karmakar, S. & Ray, D. Room-temperature orange-red phosphorescence by way of intermolecular charge transfer in single-component phenoxazine-quinoline conjugates and chemical sensing. *J. Phys. Chem. C* **122**, 21589-21597 (2018).
5. Bhattacharjee, I., Acharya, N. & Ray, D. Thermally activated delayed fluorescence and room-temperature phosphorescence in naphthyl appended carbazole-quinoline conjugates, and their mechanical regulation. *Chem. Commun.* **55**, 1899-1902 (2019).
6. Bhattacharjee, I. & Hirata, S. Highly efficient persistent room-temperature phosphorescence from heavy atom-free molecules triggered by hidden long phosphorescent antenna. *Adv. Mater.* **32**, e2001348 (2020).
7. Bian, L. et al. Color-tunable ultralong organic phosphorescence materials for visual UV-light detection. *Sci. China. Chem.* **63**, 1443-1448 (2020).
8. Bian, L. et al. Simultaneously enhancing efficiency and lifetime of ultralong organic phosphorescence materials by molecular self-assembly. *J. Am. Chem. Soc.* **140**, 10734-10739 (2018).
9. Bolton, O., Lee, K., Kim, H., Lin, K. & Kim, J. Activating efficient phosphorescence from purely organic materials by crystal design. *Nat. Chem.* **3**, 205-210 (2011).
10. Bonacchi, S. et al. Luminescent silica nanoparticles: Extending the frontiers of brightness. *Angew. Chem. Int. Ed.* **50**, 4056-4066 (2011).
11. Calhoun, D., Englander, S., Wright, W. & Vanderkooi, J. Quenching of room temperature protein phosphorescence by added small molecules. *Biochemistry* **27**, 8466-8474 (1988).
12. Cai, S. et al. Enabling long-lived organic room temperature phosphorescence in polymers by subunit interlocking. *Nat. Commun.* **10**, 4247 (2019).
13. Cai, S. et al. Hydrogen-bonded organic aromatic frameworks for ultralong phosphorescence by intralayer π-π interactions. *Angew. Chem. Int. Ed.* **57**, 4005-4009 (2018).
14. Chai, Z. et al. Abnormal room temperature phosphorescence of purely organic boron-containing compounds: The relationship between the emissive behaviorand the molecular packing, and the potential related applications. *Chem. Sci.* **8**, 8336-8344 (2017).

15. Chen, B., Zhang, X., Wang, Y., Miao, H. & Zhang, G. Aggregation-induced emission with long-lived room-temperature phosphorescence from methylene-linked organic donor-acceptor structures. *Chem. Asian J.* **14**, 751-754 (2019).
16. Chen, G. et al. Photophysical tuning of organic ionic crystals from ultralong afterglow to highly efficient phosphorescence by variation of halides. *J. Phys. Chem. Lett.* **9**, 6305-6311 (2018).
17. Chen, H. et al. Toward achieving single-molecule white electroluminescence from dual emission of fluorescence and phosphorescence. *Chem. Mater.* **32**, 4038-4044 (2020).
18. Chen, H., Ma, X., Wu, S. & Tian, H. A rapidly self-healing supramolecular polymer hydrogel with photostimulated room-temperature phosphorescence responsiveness. *Angew. Chem. Int. Ed.* **53**, 14149-14152 (2014).
19. Chen, H., Xu, L., Ma, X. &Tian, H. Room temperature phosphorescence of 4-bromo-1,8-naphthalic anhydride derivative-based polyacrylamide copolymer with photo-stimulated responsiveness. *Poly. Chem.* **7**, 3989-3992 (2016).
20. Chen, H., Yao, X., Ma, X. & Tian, H. Amorphous, efficient, room-temperature phosphorescent metal-free polymers and their applications as encryption ink. *Adv. Opt. Mater.* **4**, 1397-1401 (2016).
21. Chen, J. et al. Achievement of persistent and efficient organic room-temperature phosphorescence with temperature-response by adjusting the proportion of excited-state configurations in coupled molecules. *J. Mater. Chem. C* 8250-8254 (2019).
22. Chen, J. et al. Achieving dual-emissive and time-dependent evolutive organic afterglow by bridging molecules with weak intermolecular hydrogen bonding. *Adv. Opt. Mater.* **7**, 1801593, (2019).
23. Chen, L. et al. High contrast temperature-responsive luminescence materials from purely organic molecule with persistent room-temperature phosphorescence. *J. Lumin.* **230**, 117731, (2021).
24. Chen, W. C. et al. A novel spiro-annulated benzimidazole host for highly efficient blue phosphorescent organic light-emitting devices. *Chem. Commun.* **54**, 4541-4544 (2018).
25. Chen, X. et al. Aggregation-induced dual emission and unusual luminescence beyond excimer emission of poly(ethylene terephthalate). *Macromolecules* **51**, 9035-9042 (2018).
26. Chen, Y. et al. Tunable phosphorescence/fluorescence dual emissions of organic isoquinoline-benzophenone doped systems by alkoxy engineering. *Chemistry* **26**, 17376-17380 (2020).
27. Cheng, Z. et al. Ultralong phosphorescence from organic ionic crystals under ambient conditions. *Angew. Chem. Int. Ed.* **57**, 678-682 (2018).
28. Clapp, D. et al. The phosphorescence of tetraphenylmethane and certain related substances. *J.Am.Chem.Soc.* **61**, 523-524 (1939).
29. Das, P., Encinas, M. & Scaiano, J. Laser flash-photolysis study of the reactions of carbonyl triplets with phenols and photochemistry of p-hydroxypropiophenone. *J. Am. Chem. Soc.* **103**, 4154-4162 (1981).
30. D'agostino, S., Spinelli, F., Taddei, P., Ventura, B. & Grepioni, F. Ultralong organic phosphorescence in the solid state: The case of triphenylene cocrystals with halo- and dihalo-penta/tetrafluorobenzene. *Cryst. Growth Des.* **19**, 336-346 (2018).
31. Deng, Y., Li, P., Li, J., Sun, D. & Li, H. Color-tunable aqueous room-temperature phosphorescence supramolecular assembly. *ACS Appl. Mater. Interfaces* **13**, 14407-14416 (2021).
32. Deng, Y. et al. Long lifetime pure organic phosphorescence based on water soluble carbon dots. *Chem. Commun.* **49**, 5751-5753 (2013).
33. Ding, B., Gao, H., Wang, C. & Ma, X. Reversible room-temperature phosphorescence in response to light stimulation based on a photochromic copolymer. *Chem. Commun.* **57**, 3154-3157 (2021).
34. Dou, X. et al. Clustering-triggered emission and persistent room temperature phosphorescence of sodium alginate. *Biomacromolecules* **19**, 2014-2022 (2018).
35. Dou, X. et al. Color-tunable, excitation-dependent, and time-dependent afterglows from pure organic amorphous polymers. *Adv. Mater.* **32**, e2004768 (2020).
36. Feng, C. et al. Excited-state modulation for controlling fluorescence and phosphorescence pathways toward white-light emission. *Adv. Opt. Mater.* **7**, 1900767, (2019).
37. Fermi, A. et al. Molecular asterisks with a persulfurated benzene core are among the strongest organic phosphorescent

emitters in the solid state. *Dyes Pigments* **110**, 113-122 (2014).
38. Forni, A., Lucenti, E., Botta, C. & Cariati, E. Metal free room temperature phosphorescence from molecular self-interactions in the solid state. *J. Mater. Chem. C.* **6**, 4603-4626 (2018).
39. Fries, F. et al. Dissecting tetra-n-phenylbenzidine: Biphenyl as the origin of room temperature phosphorescence. *J. Phys. Chem. A*. **124**, 479-485 (2020).
40. Gahlaut, R. et al. Luminescence characteristics and room temperature phosphorescence of naphthoic acids in polymers. *J. Lumin.* **138**, 122-128 (2013).
41. Gan, N. et al. Manipulating the stacking of triplet chromophores in the crystal form for ultralong organic phosphorescence. *Angew. Chem. Int. Ed.* **58**, 14140-14145 (2019).
42. Gao, H. et al. Boosting room temperature phosphorescence performance by alkyl modification for intravital orthotopic lung tumor imaging. *Small.* **17**, e2005449 (2021).
43. Gao, H., Zhao, X., Wang, H., Pang, X. & Jin, W. Phosphorescent cocrystals assembled by 1,4-diiodotetrafluorobenzene and fluorene and its heterocyclic analogues based on C-I···π halogen bonding. *Cryst. Growth Des.* **12**, 4377-4387 (2012).
44. Gao, R., Fang, X. & Yan, D. Direct white-light emitting room-temperature-phosphorescence thin films with tunable two-color polarized emission through orientational hydrogen-bonding layer-by-layer assembly. *J. Mater. Chem. C.* **6**, 4444-4449 (2018).
45. Gao, Y. et al. Matrix-free and highly efficient room-temperature phosphorescence of nitrogen-doped carbon dots. *Langmuir.* **34**, 12845-12852 (2018).
46. Gao, Y., Zhang, H., Shuang, S. & Dong, C. Visible-light-excited ultralong-lifetime room temperature phosphorescence based on nitrogen-doped carbon dots for double anticounterfeiting. *Adv. Opt. Mater.* **8**, 1901557, (2020).
47. Garain, S., Kuila, S., Sinhababu, A. & George, S. Room temperature phosphorescence from heavy atom free benzophenone boronic ester derivatives. *Bull. Mater. Sci.* **43**, 318, (2020).
48. Gb, S. & Gonnelli, M. Tryptophan phosphorescence in fluid solution. *J. Am. Chem. Soc.* **117**, 7646-7651 (1995).
49. Gong, Y. et al. Achieving persistent room temperature phosphorescence and remarkable mechanochromism from pure organic luminogens. *Adv. Mater.* **27**, 6195-6201 (2015).
50. Gong, Y., Chen, H., Ma, X. & Tian, H. A cucurbit[7]uril based molecular shuttle encoded by visible room-temperature phosphorescence. *ChemPhysChem.* **17**, 1934-1938 (2016).
51. Gong, Y. et al. Crystallization-induced phosphorescence of benzils at room temperature. *Sci. China. Chem.* **56**, 1183-1186 (2013).
52. Gong, Y. et al. Crystallization-induced dual emission from metal-and heavy atom-free aromatic acids and esters. *Chem. Sci.* **6**, 4438-4444 (2015).
53. Gu, L. et al. Prolonging the lifetime of ultralong organic phosphorescence through dihydrogen bonding. *J. Mater. Chem. C.* **6**, 226-233 (2018).
54. Gu, L. et al. Dynamic ultralong organic phosphorescence by photoactivation. *Angew. Chem. Int. Ed.* **57**, 8425-8431 (2018).
55. Gu, L. et al. Colour-tunable ultra-long organic phosphorescence of a single-component molecular crystal. *Nat. Photon.* 13, 406-411 (2019).
56. Guo, S. et al. Recent progress in pure organic room temperature phosphorescence of small molecular host-guest systems. *ACS Mater. Lett.* **3**, 379-397 (2021).
57. Hall, L. Spin-lattice relaxation and the decay of pyrazine phosphorescence at low temperatures. *J. Chem. Phys.* **48**, 1395-1396 (1968).
58. Hamzehpoor, E. & Perepichka, D. Crystal engineering of room temperature phosphorescence in organic solids. *Angew. Chem. Int. Ed.* **59**, 9977-9981 (2020).
59. Han, J. et al. Small-molecule-doped organic crystals with long-persistent luminescence. *Adv. Funct. Mater.* **29**, 1902503, (2019).
60. He, G. et al. Crystallization-induced red phosphorescence and grinding-induced blue-shifted emission of a

benzobis(1,2,5-thiadiazole)-thiophene conjugate. *ACS Omega.* **4**, 344-351 (2019).

61. He, W., Sun, X. & Cao, X. Construction and multifunctional applications of visible-light-excited multicolor long afterglow carbon dots/boron oxide composites. *ACS Sustain. Chem. Eng.* **9**, 4477-4486 (2021).
62. He, Z., Li, W., Chen, G., Zhang, Y. & Yuan, W. Polymorphism dependent triplet-involved emissions of a pure organic luminogen. *Chinese Chem. Lett.* **30**, 933-936 (2019).
63. Higginbotham, H. et al. Heavy-atom-free room-temperature phosphorescent organic light-emitting diodes enabled by excited states engineering. *ACS Appl. Mater. Interfaces.* **13**, 2899-2907 (2021).
64. Hirata, S. Intrinsic analysis of radiative and room-temperature nonradiative processes based on triplet state intramolecular vibrations of heavy atom-free conjugated molecules toward efficient persistent room-temperature phosphorescence. *J. Phys. Chem. Lett.* **9**, 4251-4259 (2018).
65. Hirata, S. Roles of localized electronic structures caused by π degeneracy due to highly symmetric heavy atom-free conjugated molecular crystals leading to efficient persistent room-temperature phosphorescence. *Adv. Sci.* **6**, 1900410 (2019).
66. Hirata, S., Totani, K., Watanabe, T., Kaji, H. & Vacha, M. Relationship between room temperature phosphorescence and deuteration position in a purely aromatic compound. *Chem. Phys. Lett.* **591**, 119-125 (2014).
67. Hirata, S. & Vacha, M. Circularly polarized persistent room-temperature phosphorescence from metal-free chiral aromatics in air. *J. Phys. Chem. Lett.* **7**, 1539-1545 (2016).
68. Hirata, S. & Vacha, M. White afterglow room-temperature emission from an isolated single aromatic unit under ambient condition. *Adv. Opt. Mater.* **5**, 1600996, (2017).
69. Hu, Y. et al. One-step synthesis of cyclic compounds towards easy room-temperature phosphorescence and deep blue thermally activated delayed fluorescence. *Chem. Commun.* **54**, 7850-7853 (2018).
70. Jia, X. et al. Photoexcitation-controlled self-recoverable molecular aggregation for flicker phosphorescence. *Proc. Natl. Acad. Sci.* **116**, 4816-4821 (2019).
71. Jiang, K., Wang, Y., Cai, C. & Lin, H. Conversion of carbon dots from fluorescence to ultralong room-temperature phosphorescence by heating for security applications. *Adv. Mater.* **30**, e1800783 (2018).
72. Joshi, R., Meitei, O. R., Jadhao, M., Kumar, H.&Ghosh, S. K. Conformation controlled turn on-turn off phosphorescence in a metal-free biluminophore: Thriving the paradox that exists for organic compounds. *Phys. Chem. Chem. Phys.* **18**, 27910-27920 (2016).
73. Kanosue, K. & Ando, S. Polyimides with heavy halogens exhibiting room-temperature phosphorescence with very large stokes shifts. *ACS Macro. Lett.* **5**, 1301-1305 (2016).
74. Kanosue, K. et al. A colorless semi-aromatic polyimide derived from a sterically hindered bromine-substituted dianhydride exhibiting dual fluorescence and phosphorescence emission. *Mate.r Chem. Front.* **3**, 39-49 (2019).
75. Katsurada, Y., Hirata, S., Totani, K., Watanabe, T. & Vacha, M. Photoreversible on-off recording of persistent room-temperature phosphorescence. *Adv. Opt. Mater.* **3**, 1726-1737 (2015).
76. Kim, C. L. et al. Dual mode radiative transition from a phenoselenazine derivative and electrical switching of the emission mechanism. *J. Phys. Chem. Lett.* **11**, 5591-5600 (2020).
77. Koch, M. et al. Metal-free triplet phosphors with high emission efficiency and high tunability. *Angew. Chem. Int. Ed.* **53**, 6378-6382 (2014).
78. Kuila, S., Garain, S., Bandi, S. & George, S. All-organic, temporally pure white afterglow in amorphous films using complementary blue and greenish-yellow ultralong room temperature phosphors. *Adv. Funct. Mater.* **30**, 2003693, (2020).
79. Kuno, S., Akeno, H., Ohtani, H. & Yuasa, H. Visible room-temperature phosphorescence of pure organic crystals via a radical-ion-pair mechanism. *Phys. Chem. Chem. Phys.* **17**, 15989-15995 (2015).
80. Kuno, S., Kanamori, T., Yijing, Z., Ohtani, H. & Yuasa, H. Long persistent phosphorescence of crystalline phenylboronic acid derivatives: Photophysics and a mechanistic study. *ChemPhotoChem.* **1**, 102-106 (2017).
81. Kwon, M., Lee, D., Seo, S., Jung, J. & Kim, J. Tailoring intermolecular interactions for efficient room-temperature phosphorescence from purely organic materials in amorphous polymer matrices. *Angew. Chem. Int. Ed.* **53**, 11177-

11181 (2014).
82. Kwon, M. et al. Suppressing molecular motions for enhanced room-temperature phosphorescence of metal-free organic materials. *Nat. Commun.* **6**, 8947 (2015).
83. Lai, Y. et al. Effective internal and external modulation of nontraditional intrinsic luminescence. *Small.* **16**, e2005035 (2020).
84. Lee, D. et al. Room temperature phosphorescence of metal-free organic materials in amorphous polymer matrices. *J. Am. Chem. Soc.* **135**, 6325-6329 (2013).
85. Lei, Y. et al. Revealing insight into long-lived room-temperature phosphorescence of host-guest systems. *J. Phys. Chem. Lett.* **10**, 6019-6025 (2019).
86. Lei, Y. et al. Efficient and organic host-guest room-temperature phosphorescence: Tunable triplet-singlet crossing and theoretical calculations for molecular packing. *Chem. Sci.* **12**, 6518-6525 (2021).
87. Li, C. et al. Reversible luminescence switching of an organic solid: Controllable on-off persistent room temperature phosphorescence and stimulated multiple fluorescence conversion. *Adv. Opt. Mater.* **3**, 1184-1190 (2015).
88. Li, D. et al. Amorphous metal-free room-temperature phosphorescent small molecules with multicolor photoluminescence via a host-guest and dual-emission strategy. *J. Am. Chem. Soc.* **140**, 1916-1923 (2018).
89. Li, J. et al. Transient and persistent room-temperature mechanoluminescence from a white-light-emitting aiegen with tricolor emission switching triggered by light. *Angew. Chem. Int. Ed.* **57**, 6449-6453 (2018).
90. Li, J., Zhang, H., Zhang, Y., Zhou, W. & Liu, Y. Room-temperature phosphorescence and reversible white light switch based on a cyclodextrin polypseudorotaxane xerogel. *Adv. Opt. Mater.* **7**, 1900589, (2019).
91. Li, L., Wu, W., Liu, Z. & Jin, W. Effect of geometry factors on the priority of σ-hole···π and π-hole···π bond in phosphorescent cocrystals formed by pyrene or phenanthrene and trihaloperfluorobenzenes. *New J. Chem.* **42**, 10633-10641 (2018).
92. Li, M. et al. Achieving high-efficiency purely organic room-temperature phosphorescence materials by boronic ester substitution of phenoxathiine. *Chem. Commun.* **55**, 7215-7218 (2019).
93. Li, M. et al. Prolonging ultralong organic phosphorescence lifetime to 2.5 s through confining rotation in molecular rotor. *Adv. Opt. Mater.* **7**, 1800820, (2019).
94. Li, T. & Ma, X. Host-guest supramolecular amphiphile enhanced photodecomposition with responsive room-temperature phosphorescence signals. *Dyes Pigments.* **148**, 306-312 (2018).
95. Li, W. et al. Selective expression of chromophores in a single molecule: Soft organic crystals exhibiting full-colour tunability and dynamic triplet-exciton behaviours. *Angew. Chem. Int. Ed.* **59**, 3739-3745 (2020).
96. Li, X., Wu, Y., Zhao, Y. & Yu, Z. Employing cholesterol copolymerization strategy for a thermally processable organic room-temperature phosphorescence material. *Adv. Opt. Mater.* **9**, 2001893, (2021).
97. Li, Z. & Zhang, X. Research on long-lived room-temperature phosphorescence of carbazole-naphthalimide polylactides. *Polymers.* **12**, 790, (2020).
98. Liao, Q. et al. 9,9-dimethylxanthene derivatives with room-temperature phosphorescence: Substituent effects and emissive properties. *Angew. Chem. Int. Ed.* **59**, 9946-9951 (2020).
99. Lin, X., Wang, J., Ding, B., Ma, X. & Tian, H. Tunable-emission amorphous room-temperature phosphorescent polymers based on thermoreversible dynamic covalent bonds. *Angew. Chem. Int. Ed.* **60**, 3459-3463 (2021).
100. Ling, K. et al. Controllable multiemission with ultralong organic phosphorescence in crystal by isomerization. *Adv. Opt. Mater.* **7**, 1901076, (2019).
101. Liu, H. et al. Controllably realizing elastic/plastic bending based on a room-temperature phosphorescent waveguiding organic crystal. *Chem. Sci.* **10**, 227-232 (2019).
102. Liu, H. et al. Efficient room-temperature phosphorescence based on a pure organic sulfur-containing heterocycle: Folding-induced spin-orbit coupling enhancement. *Mater. Chem. Front.* **2**, 1853-1858 (2018).
103. Liu, H., Liu, W., Ando, N., Yamaguchi, S. & Zhang, H. Organic phosphorescent polymorphs induced by various halogen bonds with stimuli-responsive single/dual phosphorescence switching. *J. Mater. Chem. C.* **9**, 2738-2743 (2021).
104. Liu, J. et al. Crystal-state quad-mode triplet emissions of d-a-a'-d type phosphors with AIEE and visible-light-excited

persistent phosphorescence. *Dyes Pigments.* **188**, 109178, (2021).

105. Liu, X. et al. Pure room temperature phosphorescence emission of an organic host–guest doped system with a quantum efficiency of 64%. *J. Mater. Chem. C.* **9**, 3391-3395 (2021).
106. Liu, Z. et al. Sulfur-based intramolecular hydrogen-bond: Excited-state hydrogen-bond on/off switch with dual room-temperature phosphorescence. *J. Am. Chem. Soc.* **141**, 9885-9894 (2019).
107. Locke, R. & Lim, E. Phosphorescence of naphthalene and related compounds in fluid media: excimer phosphorescence or phosphorescence from a biacetyl-like impurity? *Chem. Phys. Lett.* **138**, 489-493 (1987).
108. Louis, M. et al. Biluminescence under ambient conditions: Water-soluble organic emitter in high-oxygen-barrier polymer. *Adv. Opt. Mater.* **8**, 2000427, (2020).
109. Louis, M. et al. Blue-light-absorbing thin films showing ultralong room-temperature phosphorescence. *Adv. Mater.* **31**, e1807887 (2019).
110. Lewis, G. & Kasha, M. Phosphorescence and the triplet state. *J. Am. Chem. Soc.* **66**, 2100-2116 (1944).
111. Lucenti, E. et al. Cyclic triimidazole derivatives: Intriguing examples of multiple emissions and ultralong phosphorescence at room temperature. *Angew. Chem. Int. Ed.* **56**, 16302-16307 (2017).
112. Lucenti, E. et al. H-aggregates granting crystallization-induced emissive behavior and ultralong phosphorescence from a pure organic molecule. *J. Phys. Chem. Lett.* **8**, 1894-1898 (2017).
113. Luo, W. et al. Crystallization-induced phosphorescence, remarkable mechanochromism, and grinding enhanced emission of benzophenone-aromatic amine conjugates. *Chinese Chem. Lett.* **29**, 1533-1536 (2018).
114. Ma, C. et al. Insight into chirality on molecular stacking for tunable ultralong organic phosphorescence. *J. Mater. Chem. C.* **6**, 10179-10183 (2018).
115. Ma, L., Sun, S., Ding, B., Ma, X. & Tian, H. Highly efficient room-temperature phosphorescence based on single-benzene structure molecules and photoactivated luminescence with afterglow. *Adv. Funct. Mater.* **31**, (2021).
116. Ma, X. et al. A color-tunable single molecule white light emitter with high luminescence efficiency and ultra-long room temperature phosphorescence. *J. Mater. Chem. C.* **9**, 727-735 (2021).
117. Ma, X. et al. Reversible two-channel mechanochromic luminescence for a pyridinium-based white-light emitter with room-temperature fluorescence-phosphorescence dual emission. *Phys. Chem. Chem. Phys.* **21**, 14728-14733 (2019).
118. Ma, X., Xu, C., Wang, J. & Tian, H. Amorphous pure organic polymers for heavy-atom-free efficient room-temperature phosphorescence emission. *Angew. Chem. Int. Ed.* **57**, 10854-10858 (2018).
119. Ma, X. et al. Supramolecular pins with ultralong efficient phosphorescence. *Adv. Mater.* **33**, e2007476 (2021).
120. Ma, X. et al. A twin-axial pseudorotaxane for phosphorescence cell imaging. *Chem. Commun.* **57**, 1214-1217 (2021).
121. Mao, Z. et al. Linearly tunable emission colors obtained from a fluorescent-phosphorescent dual-emission compound by mechanical stimuli. *Angew. Chem. Int. Ed.* **54**, 6270-6273 (2015).
122. Mccall, S. & Winefordner, J. Low-temperature filter paper phosphorescence. *Anal. Chem.* **55**, 391-393 (1983).
123. Menning, S. *et al.* Twisted tethered tolanes: Unanticipated long-lived phosphorescence at 77 K. *J. Am. Chem. Soc.* **135**, 2160-2163 (2013).
124. Miao, Y. *et al.* Ultralong and color-tunable room-temperature phosphorescence based on commercial melamine for anticounterfeiting and information recognition. *Anal. Chem.* **93**, 4075-4083 (2021).
125. Mukherjee, S. & Thilagar, P. Recent advances in purely organic phosphorescent materials. *Chem. Commun.* **51**, 10988-11003 (2015).
126. Nakayama, T., Sakurai, K., Ushida, K., Kawatsura, K. & Hamanoue, K. Dual phosphorescence of benzophenone at 77 K in the mixed solvent of 2,2,2-trifluoroethanol and water. Chem. Phys. Lett. **164**, 557-561 (1989).
127. Nara, M., Orita, R., Ishige, R. & Ando, S. White-light emission and tunable luminescence colors of polyimide copolymers based on fret and room-temperature phosphorescence. *ACS Omega.* **5**, 14831-14841 (2020).
128. Nicol, A. et al. Ultrafast delivery of aggregation-induced emission nanoparticles and pure organic phosphorescent nanocrystals by saponin encapsulation. *J. Am. Chem. Soc.* **139**, 14792-14799 (2017).
129. Nidhankar, A. et al. Self-assembled helical arrays for the stabilization of the triplet state. *Angew. Chem. Int. Ed.* **59**, 13079-13085 (2020).

130. Nitti, A. et al. Crystallization-induced room-temperature phosphorescence in fumaramides. *CrystEngComm.* **22**, 7782-7785 (2020).
131. Olness, D. & Sponer, H. Phosphorescence lifetime studies in some organic crystals at low temperatures. *J. Chem. Phys.* **38**, 1779-1782 (1963).
132. Ogoshi, T. et al. Ultralong room-temperature phosphorescence from amorphous polymer poly(styrene sulfonic acid) in air in the dry solid state. *Adv. Funct. Mater.* **28**, 1707369, (2018).
133. Ono, T., Taema, A., Goto, A. & Hisaeda, Y. Switching of monomer fluorescence, charge-transfer fluorescence, and room-temperature phosphorescence induced by aromatic guest inclusion in a supramolecular host. *Chemistry.* **24**, 17487-17496 (2018).
134. Papp, S. & Vanderkooi, J. Tryptophan phosphorescence at room temperature as a tool to study protein structure and dynamics. *Photochem. Photobiol.* **49**, 775-784 (2010).
135. Pan, S. et al. Ultralong room-temperature phosphorescence from supramolecular behavior via intermolecular electronic coupling in pure organic crystals. *J. Phys. Chem. Lett.* **9**, 3939-3945 (2018).
136. Pander, P., Swist, A., Soloducho, J. & Dias, F. Room temperature phosphorescence lifetime and spectrum tuning of substituted thianthrenes. *Dyes Pigments.* **142**, 315-322 (2017).
137. Pang, X., Wang, H., Wang, W. & Jin, W. Phosphorescent π-hole···π bonding cocrystals of pyrene with halo-perfluorobenzenes (F, Cl, Br, I). *Cryst. Growth Des.* **15**, 4938-4945 (2015).
138. Pashazadeh, R. et al. An iminodibenzyl-quinoxaline-iminodibenzyl scaffold as a mechanochromic and dual emitter: Donor and bridge effects on optical properties. *Chem. Commun.* **54**, 13857-13860 (2018).
139. Paul, L., Chakrabarti, S. & Ruud, K. Origin of dual-peak phosphorescence and ultralong lifetime of 4,6-diethoxy-2-carbazolyl-1,3,5-triazine. *J. Phys. Chem. Lett.* **8**, 1253-1258 (2017).
140. Ramasamy, S. & Hurtubise, R. Matrix and solvent effects on the room-temperature phosphorescence of nitrogen heterocycles. *Anal. Chem.* 1982, 54(14):2477-2481.
141. Riebe, S. et al. Aromatic thioethers as novel luminophores with aggregation-induced fluorescence and phosphorescence. *Chemistry.* **23**, 13660-13668 (2017).
142. Ruan, Z. et al. Luminous butterflies: Rational molecular design to optimize crystal packing for dramatically enhanced room-temperature phosphorescence. *Adv. Opt. Mater.* **9**, 2001549, (2021).
143. Salla, C. et al. Persistent solid-state phosphorescence and delayed fluorescence at room temperature by a twisted hydrocarbon. *Angew. Chem. Int. Ed.* **58**, 6982-6986 (2019).
144. Samonina-Kosicka, J., Derosa, C., Morris, W., Fan, Z. & Fraser, C. Dual-emissive difluoroboron naphthyl-phenyl beta-diketonate polylactide materials: Effects of heavy atom placement and polymer molecular weight. *Macromolecules.* **47**, 3736-3746 (2014).
145. Schulman, E. & Walling, C. Phosphorescence of adsorbed ionic organic molecules at room temperature. *Science.* **178**, 53-54 (1972).
146. Serevičius, T. et al. Room temperature phosphorescence vs. Thermally activated delayed fluorescence in carbazole-pyrimidine cored compounds. *J. Mater. Chem. C.* **6**, 11128-11136 (2018).
147. She, P. et al. Controlling organic room temperature phosphorescence through external heavy‐atom effect for white light emission and luminescence printing. *Adv. Opt. Mater.* **8**, 1901437, (2019).
148. Shen, C., Zhang, Y., Yong, G. &Zhao, Y. Spontaneous resolution in a new chiral purely organic crystal containing homochiral helical chains: Synthesis, crystal structure, and phosphorescence. *J. Mol. Struct.* **1084**, 340-344 (2015).
149. Shen, F. et al. Purely organic light-harvesting phosphorescence energy transfer by beta-cyclodextrin pseudorotaxane for mitochondria targeted imaging. *Chem. Sci.* **12**, 1851-1857 (2020).
150. Shi, H. et al. Enhancing organic phosphorescence by manipulating heavy-atom interaction. *Cryst. Growth Des.* **16**, 808-813 (2016).
151. Shi, H. et al. Highly efficient ultralong organic phosphorescence through intramolecular-space heavy-atom effect. *J. Phys. Chem. Lett.* **10**, 595-600 (2019).
152. Shi, H. et al. A highly efficient red metal-free organic phosphor for time-resolved luminescence imaging and

photodynamic therapy. *ACS Appl. Mater. Interfaces.* **11**, 18103-18110 (2019).
153. Shimizu, M., Kimura, A. & Sakaguchi, H. Room-temperature phosphorescence of crystalline 1,4-bis(aroyl)-2,5-dibromobenzenes. *Eur. J. Org. Chem.* **2016**, 467-473 (2016).
154. Shoji, Y. et al. Unveiling a new aspect of simple arylboronic esters: Long-lived room-temperature phosphorescence from heavy-atom-free molecules. *J. Am. Chem. Soc.* **139**, 2728-2733 (2017).
155. Singh, M. et al. Recent advances of cocrystals with room temperature phosphorescence. *Adv. Opt. Mater.* **9**, 2002197, (2021).
156. Song, Y., Wang, J., Chen, L. & Yang, P. Ammonium pentaborate crystals with adjustable and bright phosphorescence and long lifetime. *J. Lumin.* **225**, 117325, (2020).
157. Suter, G. & Wild, U. The dual phosphorescence of benzophenazine in ethanol. *Chem. Phys.* **73**, 421-429 (1982).
158. Su, Y. et al. Excitation-dependent long-life luminescent polymeric systems under ambient conditions. *Angew. Chem. Int. Ed.* **59**, 9967-9971 (2020).
159. Sun, C. et al. Twisted molecular structure on tuning ultralong organic phosphorescence. *J. Phys. Chem. Lett.* **9**, 335-339 (2018).
160. Sun, H. et al. Clustering-triggered ultralong room-temperature phosphorescence of organic crystals through halogen-mediated molecular assembly. *J. Phys. Chem. Lett.* **11**, 4962-4969 (2020).
161. Sun, Q. et al. Remarkable photo-induced crystal transition accompanying with room temperature phosphorescence change. *Dyes Pigments.* **170**, 107600, (2019).
162. Sun, Y. et al. Ultralong lifetime and efficient room temperature phosphorescent carbon dots through multi-confinement structure design. *Nat. Commun.* **11**, 5591 (2020).
163. Tan, J., Yi, Z., Ye, Y., Ren, X. & Li, Q. Achieving red room temperature afterglow carbon dots in composite matrices through chromophore conjugation degree controlling. *J. Lumin.* **223**, 117267, (2020).
164. Tang, L. et al. X-ray excited ultralong room-temperature phosphorescence for organic afterglow scintillators. *Chem. Commun.* **56**, 13559-13562 (2020).
165. Tao, S. et al. Design of metal-free polymer carbon dots: A new class of room-temperature phosphorescent materials. *Angew. Chem. Int. Ed.* **57**, 2393-2398 (2018).
166. Thomas, H. et al. Aromatic phosphonates: A novel group of emitters showing blue ultralong room temperature phosphorescence. *Adv. Mater.* **32**, e2000880 (2020).
167. Tian, D., Zhu, Z., Xu, L., Cong, H. & Zhu, J. Intramolecular electronic coupling for persistent room-temperature luminescence for smartphone based time-gated fingerprint detection. *Mater. Horiz.* **6**, 1215-1221 (2019).
168. Tian, S. et al. Utilizing d-pπ bonds for ultralong organic phosphorescence. *Angew. Chem. Int. Ed.* **58**, 6645-6649 (2019).
169. Tian, Y. et al. The initial attempt to reveal the emission processes of both mechanoluminescence and room temperature phosphorescence with the aid of circular dichroism in solid state. *Sci. China. Chem.* **64**, 445-451 (2021).
170. Tian, Z. et al. Multilevel data encryption using thermal-treatment controlled room temperature phosphorescence of carbon dot/polyvinylalcohol composites. *Adv. Sci.* **5**, 1800795 (2018).
171. Villa, M. et al. Bright phosphorescence of all-organic chromophores confined within water-soluble silica nanoparticles. *J. Phys. Chem. C.* **123**, 29884-29890 (2019).
172. Wakchaure, V. et al. Mechano-responsive room temperature luminescence variations of boron conjugated pyrene in air. *Chem. Commun.* **54**, 6028-6031 (2018).
173. Wang, D. et al. Excitation-dependent triplet-singlet intensity from organic host-guest materials: Tunable color, white-light emission, and room-temperature phosphorescence. *J. Phys. Chem. Lett.* **12**, 1814-1821 (2021).
174. Wang, H. et al. Amorphous ionic polymers with color-tunable ultralong organic phosphorescence. *Angew. Chem. Int. Ed.* **58**, 18776-18782 (2019).
175. Wang, H., Wang, H., Yang, X., Wang, Q. & Yang, Y. Ion-unquenchable and thermally "on-off" reversible room temperature phosphorescence of 3-bromoquinoline induced by supramolecular gels. *Langmuir.* **31**, 486-491 (2015).
176. Wang, J., Huang, Z., Ma, X. & Tian, H. Visible-light-excited room-temperature phosphorescence in water by cucurbit[8]uril-mediated supramolecular assembly. *Angew. Chem. Int. Ed.* **59**, 9928-9933 (2020).

177. Wang, J. et al. Bromine-substituted fluorene: Molecular structure, Br-Br interactions, room-temperature phosphorescence, and tricolor triboluminescence. *Angew. Chem. Int. Ed.* **57**, 16821-16826 (2018).
178. Wang, Q. et al. Reevaluating protein photoluminescence: Remarkable visible luminescence upon concentration and insight into the emission mechanism. *Angew. Chem. Int. Ed.* **58**, 12667-12673 (2019).
179. Wang, S. et al. Covalent organic frameworks: A platform for the experimental establishment of the influence of intermolecular distance on phosphorescence. *J. Mater. Chem. C.* **6**, 5369-5374 (2018).
180. Wang, T. et al. Thermochromic aggregation-induced dual phosphorescence via temperature-dependent sp^3-linked donor-acceptor electronic coupling. *Nat. Commun.* **12**, 1364 (2021).
181. Wang, T. et al. Dual-emissive waterborne polyurethanes prepared from naphthalimide derivative. *Polymers.* **9**, 411, (2017).
182. Wang, X. et al. Multicolor ultralong organic phosphorescence through alkyl engineering for 4D coding applications. *Chem. Mater.* **31**, 5584-5591 (2019).
183. Wang, X. et al. Pure organic room temperature phosphorescence from unique micelle-assisted assembly of nanocrystals in water. *Adv. Funct. Mater.* **30**, 1907282, (2020).
184. Wang, X. et al. Pure organic room temperature phosphorescence from excited dimers in self-assembled nanoparticles under visible and near-infrared irradiation in water. *J. Am. Chem. Soc.* **141**, 5045-5050 (2019).
185. Wang, X. et al. Organic phosphors with bright triplet excitons for efficient x-ray-excited luminescence. *Nat. Photon.* **15**, 187-192 (2021).
186. Wang, Y. et al. Conformational torsion, intramolecular hydrogen bonding and solvent effects in intersystem crossing of singlet-triplet excited states for heavy-atom-free organic long persistent luminescence. *J. Mol. Liq.* **326**, 115291, (2021).
187. Wang, Y. et al. Nonconventional luminophores with unprecedented efficiencies and color-tunable afterglows. *Mater. Horiz.* **7**, 2105-2112 (2020).
188. Wang, Z. et al. Ultralong-lived room temperature phosphorescence from n and p codoped self-protective carbonized polymer dots for confidential information encryption and decryption. *J. Mater. Chem. C* **9**, 4847-4853 (2021).
189. Wang, Z. et al. Color-tunable polymeric long-persistent luminescence based on polyphosphazenes. *Adv. Mater.* **32**, e1907355 (2020).
190. Ward, J. et al. The interplay of thermally activated delayed fluorescence (TADF) and room temperature organic phosphorescence in sterically-constrained donor-acceptor charge-transfer molecules. *Chem. Commun.* **52**, 2612-2615 (2016).
191. Ward, J. et al. Bond rotations and heteroatom effects in donor-acceptor-donor molecules: Implications for thermally activated delayed fluorescence and room temperature phosphorescence. *J. Org. Chem.* **83**, 14431-14442 (2018).
192. Wei, J. et al. Induction of strong long-lived room-temperature phosphorescence of n-phenyl-2-naphthylamine molecules by confinement in a crystalline dibromobiphenyl matrix. *Angew. Chem. Int. Ed.* **55**, 15589-15593 (2016).
193. Wei, P. et al. New wine in old bottles: Prolonging room-temperature phosphorescence of crown ethers by supramolecular interactions. *Angew. Chem. Int. Ed.* **59**, 9293-9298 (2020).
194. Wen, Y. et al. Modulating room temperature phosphorescence by oxidation of thianthrene to achieve pure organic single-molecule white-light emission. *CCS Chem.* **3**, 1940-1948 (2021).
195. Wen, Y. et al. Achieving highly efficient pure organic single-molecule white-light emitter: The coenhanced fluorescence and phosphorescence dual emission by tailoring alkoxy substituents. *Adv. Opt. Mater.* **8**, 1901995, (2020).
196. Wen, Y. et al. One-dimensional π-π stacking induces highly efficient pure organic room-temperature phosphorescence and ternary-emission single-molecule white light. *J. Mater. Chem. C.* **7**, 12502-12508 (2019).
197. Wu, B. et al. Ultralong and high-efficiency room temperature phosphorescence of organic-phosphors-doped polymer films enhanced by 3D network. *Adv. Opt. Mater.* **8**, 2001192, (2020).
198. Wu, H. et al. Multidimensional structure conformation of persulfurated benzene for highly efficient phosphorescence. *ACS Appl. Mater. Interfaces.* **13**, 1314-1322 (2021).
199. Wu, H. et al. Crystal multi-conformational control through deformable carbon-sulfur bond for singlet-triplet emissive

tuning. *Angew. Chem. Int. Ed.* **58**, 4328-4333 (2019).

200. Wu, H. et al. Achieving amorphous ultralong room temperature phosphorescence by coassembling planar small organic molecules with polyvinyl alcohol. *Adv. Funct. Mater.* **29**, 1807243, (2019).
201. Wu, H. et al. Molecular phosphorescence in polymer matrix with reversible sensitivity. *ACS Appl. Mater. Interfaces.* **12**, 20765-20774 (2020).
202. Wu, H. et al. Molecular stacking dependent phosphorescence-fluorescence dual emission in a single luminophore for self-recoverable mechanoconversion of multicolor luminescence. *Chem. Commun.* **53**, 2661-2664 (2017).
203. Wu, H. et al. Tuning for visible fluorescence and near-infrared phosphorescence on a unimolecular mechanically sensitive platform via adjustable CH-π interaction. *ACS Appl. Mater. Interfaces.* **9**, 3865-3872 (2017).
204. Wu, Q. et al. Self-healing amorphous polymers with room-temperature phosphorescence enabled by boron-based dative bonds. *ACS Appl. Polym. Mater.* **2**, 699-705 (2019).
205. Wu, X. et al. Exploiting racemism enhanced organic room-temperature phosphorescence to demonstrate wallach's rule in the lighting chiral chromophores. *Nat. Commun.* **11**, 2145 (2020).
206. Wu, Z. et al. Persistent room temperature phosphorescence from triarylboranes: A combined experimental and theoretical study. *Angew. Chem. Int. Ed.* **59**, 17137-17144 (2020).
207. Xia, C. et al. Carbonized polymer dots with tunable room-temperature phosphorescence lifetime and wavelength. *ACS Appl. Mater. Interfaces.* **12**, 38593-38601 (2020).
208. Xiao, F. et al. Achieving crystal-induced room temperature phosphorescence and reversible photochromic properties by strong intermolecular interactions. *J. Mater. Chem. C.* **8**, 17410-17416 (2020).
209. Xiao, L. et al. Room-temperature phosphorescence in pure organic materials: Halogen bonding switching effects. *Chemistry.* **24**, 1801-1805 (2018).
210. Xing, Y., Wang, Y., Zhou, L. & Zhu, L. Highly tunable aggregate-induced phosphorescence properties in persulfurated arenes. *Dyes Pigments.* **186**, (2021).
211. Xiong, Q. et al. Pure organic room-temperature phosphorescent N-allylquinolinium salts as anti-counterfeiting materials. *Chinese Chem. Lett.* **30**, 1387-1389 (2019).
212. Xiong, Y. et al. Designing efficient and ultralong pure organic room-temperature phosphorescent materials by structural isomerism. *Angew. Chem. Int. Ed.* **57**, 7997-8001 (2018).
213. Xu, B. et al. White-light emission from a single heavy atom-free molecule with room temperature phosphorescence, mechanochromism and thermochromism. *Chem. Sci.* **8**, 1909-1914 (2017).
214. Xu, J. et al. Reversible switching between phosphorescence and fluorescence in a unimolecular system controlled by external stimuli. *Chemistry.* **24**, 12773-12778 (2018).
215. Xu, J., Takai, A., Kobayashi, Y. & Takeuchi, M. Phosphorescence from a pure organic fluorene derivative in solution at room temperature. *Chem. Commun.* **49**, 8447-8449 (2013).
216. Xu, L. et al. Chalcogen atom modulated persistent room-temperature phosphorescence through intramolecular electronic coupling. *Chem. Commun.* **54**, 9226-9229 (2018).
217. Xu, L. et al. Ultralong organic phosphorescent nanocrystals with long-lived triplet excited states for afterglow imaging and photodynamic therapy. *ACS Appl. Mater. Interfaces.* **12**, 18385-18394 (2020).
218. Xu, L., Zou, L., Chen, H. & Ma, X. Room-temperature phosphorescence of cucurbit[7]uril recognized naphthalimide derivative. *Dyes Pigments.* **142**, 300-305 (2017).
219. Xu, M. et al. Designing hybrid chiral photonic films with circularly polarized room-temperature phosphorescence. *ACS Nano.* **14**, 11130-11139 (2020).
220. Xu, Z. et al. Controlling ultralong room temperature phosphorescence in organic compounds with sulfur oxidation state. *Chem. Sci.* **12**, 188-195 (2020).
221. Xue, P. et al. Luminescence switching of a persistent room-temperature phosphorescent pure organic molecule in response to external stimuli. *Chem. Commun.* **51**, 10381-10384 (2015).
222. Xue, P., Wang, P., Chen, P., Ding, J. & Lu, R. Enhanced room-temperature phosphorescence of triphenylphosphine derivatives without metal and heavy atoms in their crystal phase. *RSC Adv.* **6**, 51683-51686 (2016).

223. Yang, J., Fang, M. & Li, Z. Stimulus-responsive room temperature phosphorescence in purely organic luminogens. *InfoMat.* **2**, 791-806 (2020).
224. Yang, J. et al. The odd-even effect of alkyl chain in organic room temperature phosphorescence luminogens and the corresponding in vivo imaging. *Mater. Chem. Front.* **3**, 1391-1397 (2019).
225. Yang, X. & Yan, D. Strongly enhanced long-lived persistent room temperature phosphorescence based on the formation of metal-organic hybrids. *Adv. Opt. Mater.* **4**, 897-905 (2016).
226. Yang, Z. et al. Enabling dynamic ultralong organic phosphorescence in molecular crystals through the synergy between intramolecular and intermolecular interactions. *J. Mater. Chem. C.* **8**, 7384-7392 (2020).
227. Yang, Z. et al. Boosting the quantum efficiency of ultralong organic phosphorescence up to 52% via intramolecular halogen bonding. *Angew. Chem. Int. Ed.* **59**, 17451-17455 (2020).
228. Yao, W. et al. Supramolecular organic frameworks with ultralong phosphorescence via breaking π-conjugated structures. *Giant.* **1**, 100007,
229. Yao, X. et al. Room-temperature phosphorescence enabled through nacre-mimetic nanocomposite design. *Adv. Mater.* **33**, e2005973 (2021).
230. Ye, W. et al. Confining isolated chromophores for highly efficient blue phosphorescence. *Nat. Mater.* **20**, 1539-1544 (2021).
231. Yin, Z. et al. Molecular engineering through control of structural deformation for highly efficient ultralong organic phosphorescence. *Angew. Chem. Int. Ed.* **60**, 2058-2063 (2021).
232. Yong, G., Zhang, Y. & She, W. Anion-π interactions in new electron-deficient π systems: The relevance to solid phosphorescent colors. *CrystEngComm.* **14**, 3923-3929, (2012).
233. Yong, G., Zhang, Y., She, W. & Li, Y. Stacking-induced white-light and blue-light phosphorescence from purely organic radical materials. *J. Mater. Chem.* **21**, 18520-18522, (2011).
234. Yong, G., She, W. & Zhang, Y. Room-temperature phosphorescence in solution and in solid state from purely organic dyes. *Dyes Pigments.* **95**, 161-167 (2012).
235. Yong, G., Zhang, X. & She, W. Phosphorescence enhancement of organic dyes by forming β-cyclodextrin inclusion complexes: Color tunable emissive materials. *Dyes Pigments.* **97**, 65-70 (2013).
236. Yoshida, K. et al. A room-temperature phosphorescent polymer film containing a molecular web based on one-dimensional chiral stacking of a simple luminophore. *Chem. Commun.* **53**, 5044-5047 (2017).
237. Yu, X. et al. Room-temperature phosphorescent gamma-cyclodextrin-cucurbit[6]uril-cowheeled [4]rotaxanes for specific sensing of tryptophan. *Chem. Commun.* **55**, 3156-3159 (2019).
238. Yu, Y. et al. Achieving enhanced ml or rtp performance: Alkyl substituent effect on the fine-tuning of molecular packing. *Mater. Chem. Front.* **5**, 817-824 (2021).
239. Yu, Y. et al. Room-temperature-phosphorescence-based dissolved oxygen detection by core-shell polymer nanoparticles containing metal-free organic phosphors. *Angew. Chem. Int. Ed.* **56**, 16207-16211 (2017).
240. Yuan, J. et al. Direct population of triplet excited states through singlet-triplet transition for visible-light excitable organic afterglow. *Chem. Sci.* **10**, 5031-5038 (2019).
241. Yuan, J. et al. Invoking ultralong room temperature phosphorescence of purely organic compounds through h-aggregation engineering. *Mater. Horiz.* **6**, 1259-1264 (2019).
242. Yuan, J. et al. Activating intersystem crossing and aggregation coupling by CN-substitution for efficient organic ultralong room temperature phosphorescence. *J. Phys. Chem. C.* **124**, 10129-10134 (2020).
243. Yuan, Z. et al. Methanol dynamically activated room-temperature phosphorescence from a twisted 4-bromobiphenyl system. *CCS Chem.* **2**, 158-167 (2020).
244. Zang, L., Shao, W., Kwon, M. S., Zhang, Z. & Kim, J. Photoresponsive luminescence switching of metal-free organic phosphors doped polymer matrices. *Adv. Opt. Mater.* **8**, 2000654, (2020).
245. Zhang, G., Palmer, G., Dewhirst, M. & Fraser, C. A dual-emissive-materials design concept enables tumour hypoxia imaging. *Nat. Mater.* **8**, 747-751 (2009).
246. Zhang, B., Yong, G., Zhao, Y. & Zhang, X. Excitation-light-induced phosphorescent color changes of β-cyclodextrin

inclusion complexes. *Opt. Mater.* **36**, 191-197 (2013).
247. Zhang, K. et a*l*. Cyclic boron esterification: Screening organic room temperature phosphorescent and mechanoluminescent materials. *J. Mater. Chem. C.* **6**, 8733-8737 (2018).
248. Zhang, T. et al. Hydrogen bonding boosted the persistent room temperature phosphorescence of pure organic compounds for multiple applications. *J. Mater. Chem. C.* **7**, 9095-9101 (2019).
249. Zhang, T., Ma, X. & Tian, H. A facile way to obtain near-infrared room-temperature phosphorescent soft materials based on bodipy dyes. *Chem. Sci.* **11**, 482-487 (2020).
250. Zhang, T. et al. Molecular engineering for metal-free amorphous materials with room-temperature phosphorescence. *Angew. Chem. Int. Ed.* **59**, 11206-11216 (2020).
251. Zhang, T., Wu, Y. & Ma, X. Tunable multicolor room-temperature phosphorescence including white-light emission from amorphous copolymers. *Chem. Eng. J.* **412**, (2021).
252. Zhang, Y. et al. Isophthalate-based room temperature phosphorescence: From small molecule to side-chain jacketed liquid crystalline polymer. *Macromolecules.* **52**, 2495-2503 (2019).
253. Zhang, Z. et al. N-alkylcarbazoles: Homolog manipulating long-lived room-temperature phosphorescence. *J. Mater. Chem. C.* **6**, 8984-8989 (2018).
254. Zhang, Z. & Liu, Y. Ultralong room-temperature phosphorescence of a solid-state supramolecule between phenylmethylpyridinium and cucurbit[6]uril. *Chem. Sci.* **10**, 7773-7778 (2019).
255. Zhang, Z. et al. A synergistic enhancement strategy for realizing ultralong and efficient room-temperature phosphorescence. *Angew. Chem. Int. Ed.* **59**, 18748-18754 (2020).
256. Zhao, C. et al. Heavy-atom-free amorphous materials with facile preparation and efficient room-temperature phosphorescence emission. *Chem. Commun.* **55**, 5355-5358 (2019).
257. Zhen, X. et al. Ultralong phosphorescence of water-soluble organic nanoparticles for in vivo afterglow imaging. *Adv. Mater.* **29**, 1606665, (2017).
258. Zheng, H., Cao, P., Wang, Y., Lu, X. & Wu, P. Ultralong room-temperature phosphorescence from boric acid. *Angew. Chem. Int. Ed.* **60**, 9500-9506 (2021).
259. Zheng, K. et al. Multicolor ultralong room-temperature phosphorescence from pure organic emitters by structural isomerism. *Chem. Eng. J.* **408**, 127309, (2021).
260. Zheng, S., Zhu, T., Wang, Y., Yang, T. & Yuan, W. Accessing tunable afterglows from highly twisted nonaromatic organic aiegens via effective through-space conjugation. *Angew. Chem. Int. Ed.* **59**, 10018-10022 (2020).
261. Zhou, B., Zhao, Q., Tang, L. & Yan, D. Tunable room temperature phosphorescence and energy transfer in ratiometric co-crystals. *Chem. Commun.* **56**, 7698-7701 (2020).
262. Zhou, C. et al. Waterborne polyurethanes with tunable fluorescence and room-temperature phosphorescence. *ACS Appl. Mater. Interfaces.* **7**, 17209-17216 (2015).
263. Zhou, C. et al. Ternary emission of fluorescence and dual phosphorescence at room temperature: A single-molecule white light emitter based on pure organic aza-aromatic material. *Adv. Funct. Mater.* **28**, 1802407, (2018).
264. Zhou, J. et al. Organic room-temperature phosphorescence from halogen-bonded organic frameworks: Hidden electronic effects in rigidified chromophores. *Chem. Sci.* **12**, 767-773 (2020).
265. Zhou, Q. et al. Emission mechanism understanding and tunable persistent room temperature phosphorescence of amorphous nonaromatic polymers. *Mater. Chem. Front.* **3**, 257-264 (2019).
266. Zhou, W. et al. Ultralong purely organic aqueous phosphorescence supramolecular polymer for targeted tumor cell imaging. *Nat. Commun.* **11**, 4655 (2020).
267. Zhou, Y. et al. Long-lived room-temperature phosphorescence for visual and quantitative detection of oxygen. *Angew. Chem. Int. Ed.* **58**, 12102-12106 (2019).
268. Bower, E. L.-Y. and Winefordner, J. D. The effect of sample environment on the room-temperature phosphorescence of several polynuclear aromatic hydrocarbons. *Anal. Chim. Acta.* **102**, 1-13 (1978).
269. Ford, C. D. and Hurtubise, R. J. Design of a phosphoroscope and the examination of room temperature phosphorescence of nitrogen heterocycles. *Anal. Chem.* **51**, 659-663 (1979).

270. Kai, Y. and Imakubo, K. Temperature dependence of the phosphorescence lifetimes of heterogeneous tryptophan residues in globular proteins between 293 and 77 K. *PhotoChem. Photobiol.* **29**, 261-265 (1979).
271. Meyers, M. L. and Seybold, P. G. Effects of external heavy atoms and other factors on the room-temperature phosphorescence and fluorescence of tryptophan and tyrosine. *Anal. Chem.* **51**, 1609-1612 (1979).
272. Parker, R., Freelander, R. S., Schulman, E. and Dunlap, R. B. Room temperature phosphorescence of selected pteridines. *Anal. Chem.* **51**, 1921-1926 (1979).
273. Segura-Carretero, A., Cruces-Blanco, C., Cañabate-Dıaz, B., Fernández-Sánchez, J. and Fernández-Gutiérrez, A. Heavy-atom induced room-temperature phosphorescence: a straightforward methodology for the determination of organic compounds in solution. *Anal. Chim. Acta.* **417**, 19-30 (2000).
274. De La Peña, A. M., Mahedero, M. and Bautista-Sánchez, A. Host-guest room temperature phosphorescence of 1-naphthalenacetic acid included in β-cyclodextrin in presence of 1, 3-dibromopropane. *Anal. Lett.* **34**, 2391-2401 (2001).
275. Fischer, C. J., Gafni, A., Steel, D. G. and Schauerte, J. A. The triplet-state lifetime of indole in aqueous and viscous environments: significance to the interpretation of room temperature phosphorescence in proteins. *J. Am. Chem. Soc.* **124**, 10359-10366 (2002).
276. Mendonsa, S. D. and Hurtubise, R. J. A comparative study of the solid-matrix phosphorescence of heterocyclic aromatic amines in glucose glasses as a function of temperature. *J. Lumin.* **97**, 19-33 (2002).
277. Nazarov, V., Avakyan, V., Alfimov, M. and Vershinnikova, T. Long-lived room temperature phosphorescence of a naphthalene-β-cyclodextrin-adamantane complex in the presence of oxygen. *Russ. Chem. Bull.* **52**, 916-922 (2003).
278. Zhang, G. et al. Multi-emissive difluoroboron dibenzoylmethane polylactide exhibiting intense fluorescence and oxygen-sensitive room-temperature phosphorescence. *J. Am. Chem. Soc.* **129**, 8942-8943 (2007).
279. Pfister, A., Zhang, G., Zareno, J., Horwitz, A. F. and Fraser, C. L. Boron polylactide nanoparticles exhibiting fluorescence and phosphorescence in aqueous medium. *ACS Nano.* **2**, 1252-1258 (2008).
280. Ma, X., Cao, J., Wang, Q. and Tian, H. Photocontrolled reversible room temperature phosphorescence (RTP) encoding β-cyclodextrin pseudorotaxane. *Chem. Commun.* **47**, 3559-3561 (2011).
281. Al-Attar, H. A. and Monkman, A. P. Room-temperature phosphorescence from films of isolated water-soluble conjugated polymers in hydrogen-bonded matrices. *Adv. Funct. Mater.* **22**, 3824-3832 (2012).
282. Gong, Y. et al. Room temperature phosphorescence from natural products: Crystallization matters. *Sci. China Chem.* **56**, 1178-1182 (2013).
283. Maity, S. K., Bera, S., Paikar, A., Pramanik, A. and Haldar, D. Halogen bond induced phosphorescence of capped γ-amino acid in the solid state. *Chem. Commun.* **49**, 9051-9053 (2013).
284. Reineke, S. et al. Highly efficient, dual state emission from an organic semiconductor. *Appl. Phys. Lett.* 103, 163-161 (2013).
285. Bolton, O., Lee, D., Jung, J. and Kim, J. Tuning the photophysical properties of metal-free room temperature organic phosphors via compositional variations in bromobenzaldehyde/dibromobenzene mixed crystals. *Chem. Mater.* **26**, 6644-6649 (2014).
286. Ventura, B. et al. Luminescence properties of 1, 8-naphthalimide derivatives in solution, in their crystals, and in co-crystals: toward room-temperature phosphorescence from organic materials. *J. Phys. Chem. C.* 118, 18646-18658 (2014).
287. Zhang, X. et al. General design strategy for aromatic ketone-based single-component dual-emissive materials. *ACS Appl. Mater. Inter.* **6**, 2279-2284 (2014).
288. Chen, J. et al. Synergistic generation and accumulation of triplet excitons for efficient ultralong organic phosphorescence. *Angew. Chem. Int. Ed.* **61**, e202200343 (2022).
289. Dai, W. et al. Halogen bonding: a new platform for achieving multi-stimuli-responsive persistent phosphorescence. *Angew. Chem. Int. Ed.* **61**, e202200236 (2022).
290. Dai, X. Y., Huo, M., Dong, X., Hu, Y. Y. and Liu, Y. Noncovalent polymerization-activated ultrastrong near-infrared room-temperature phosphorescence energy transfer assembly in aqueous solution. *Adv. Mater.* **34**, e2203534 (2022).
291. Ding, Z. Z. et al. In situ confining citric acid-derived carbon dots for full-color room-temperature phosphorescence.

Small. **19**, 2205916 (2022).

292. Fu, X. et al. A readily obtained alternative to 1H-benzo[f]indole toward room-temperature ultralong organic phosphorescence. *Chem. Mater.* **35**, 347-357 (2022).

293. Garain, S., Sarkar, S., Chandra Garain, B., Pati, S. K. and George, S. J. Chiral arylene diimide phosphors: circularly polarized ambient phosphorescence from bischromophoric pyromellitic diimides. *Angew. Chem. Int. Ed.* **61**, e202115773 (2022).

294. Jena, S., Munthasir, A. T. M. and Thilagar, P. Ultralong room temperature phosphorescence and ultraviolet fluorescence from simple triarylphosphine oxides. *J. Mater. Chem. C.* **10**, 9124-9131 (2022).

295. Li, C., Zhu, J. and Wang, Q. Amorphous pure organic phosphorescent host-guest complexes with ultralong phosphorescence lifetime and high-temperature tolerance. *Dyes Pigments.* **204**, 110368 (2022).

296. Li, G. et al. Organic supramolecular zippers with ultralong organic phosphorescence by a dexter energy transfer mechanism. *Angew. Chem. Int. Ed.* **61**, e202113425 (2022).

297. Li, M. et al. Molecular engineering of sulfur-bridged polycyclic emitters towards tunable TADF and RTP electroluminescence. *Angew. Chem. Int. Ed.* **61**, e202209343 (2022).

298. Li, W. et al. A dish-like molecular architecture for dynamic ultralong room-temperature phosphorescence through reversible guest accommodation. *Nat. Commun.* **13**, 7423 (2022).

299. Liu, H. et al. Dual-emission of fluorescence and room-temperature phosphorescence for ratiometric and colorimetric oxygen sensing and detection based on dispersion of pure organic thianthrene dimer in polymer host. *Adv. Opt. Mater.* **10**, 2102814 (2022).

300. Liu, X. W. et al. Photo-thermo-induced room-temperature phosphorescence through solid-state molecular motion. *Nat. Commun.* **13**, 3887 (2022).

301. Lou, L. et al. H-bonding room temperature phosphorescence materials via facile preparation for water-stimulated photoluminescent ink. *Molecules.* **27**, 6482 (2022).

302. Ma, L. et al. A universal strategy for tunable persistent luminescent materials via radiative energy transfer. *Angew. Chem. Int. Ed.* **61**, e202115748 (2022).

303. Meng, X. et al. Ultralong room-temperature phosphorescence from polycyclic aromatic hydrocarbons by accelerating intersystem crossing within a rigid polymer network. *J. Mater. Chem. C.* **10**, 17620-17627 (2022).

304. Shao, W., Hao, J., Jiang, H., Zimmerman, P. M. and Kim, J. Metal-free organic triplet emitters with on-off switchable excited state intramolecular proton transfer. *Adv. Funct. Mater.* 32, 2201256 (2022).

305. Shen, F. F. et al. Macrocyclic confined purely organic room-temperature phosphorescence three-photon targeted imaging. *Adv. Opt. Mater.* **10**, 2200245 (2022).

306. Shi, Y. et al. Dynamic B/N lewis pairs: insights into the structural variations and photochromism via light-induced fluorescence to phosphorescence switching. *Angew. Chem. Int. Ed.* **61**, e202213615 (2022).

307. Sun, Y. et al. Purely organic blue room-temperature phosphorescence activated by acrylamide In situ photopolymerization. *Adv. Opt. Mater.* **10**, 2201330 (2022).

308. Wagner, J. et al. Modular nitrogen-doped concave polycyclic aromatic hydrocarbons for high-performance organic light-emitting diodes with tunable emission mechanisms. *Angew. Chem. Int. Ed.* **61**, e202202232 (2022).

309. Wang, C. et al. Poly(arylene piperidine) quaternary ammonium salts promoting stable long-lived room-temperature phosphorescence in aqueous environment. *Adv. Mater.* 34, e2204415 (2022).

310. Wang, C. et al. Photo-induced dynamic room temperature phosphorescence based on triphenyl phosphonium containing polymers. *Adv. Funct. Mater.* **32**, 2111941 (2022).

311. Wang, D. et al. Achieving color-tunable and time-dependent organic long persistent luminescence via phosphorescence energy transfer for advanced anti-counterfeiting. *Adv. Funct. Mater.* **33**, 2208895 (2022).

312. Wang, X. et al. Reversible photoswitching between fluorescence and room temperature phosphorescence by manipulating excited state dynamics in molecular aggregates. *Angew. Chem. Int. Ed.* **61**, e202114264 (2022).

313. Wang, Z. et al. Four-in-one stimulus-responsive long-lived luminescent systems based on pyrene-doped amorphous polymers. *Angew. Chem. Int. Ed.* **61**, e202203254 (2022).

314. Wang, Z. et al. Regulation of irradiation-dependent long-lived room temperature phosphorescence by controlling molecular structures of chromophores and matrix. *Adv. Opt. Mater.* 10, 2200481 (2022).
315. Wu, Z. et al. Aggregation-induced dual phosphorescence from (o-bromophenyl)-bis(2,6-dimethylphenyl)borane at room temperature. *Chemistry.* **28**, e202200525 (2022).
316. Xie, N. et al. A benzene ring-linked dimethylamino and borate ester-based molecule and organic crystal: efficient dual room-temperature phosphorescence with responsive property. *Adv. Opt. Mater.* 10, 2200767 (2022).
317. Xu, X. et al. Guest-activated quaternary ammonium salt hosts emit room temperature phosphorescence. *Chem. Commun.* **58**, 11143-11146 (2022).
318. Xu, X. and Yan, B. Base-tuning HOF-based host-guest ultralong organic phosphorescence systems with phosphorescent thermochromism using for information security and thermometer. *Adv. Opt. Mater.* 10, 2200451 (2022).
319. Xue, Z. Y. et al. Color-tunable binary copolymers manipulated by intramolecular aggregation and hydrogen bonding. *ACS Appl. Mater. Interfaces.* **14**, 53359-53369 (2022).
320. Yan, Z.-A. and Ma, X. External heavy-atom activated phosphorescence of organic luminophores in a rigid fluid matrix. *ACS Mater. Lett.* **4**, 2555-2561 (2022).
321. Yuan, S. et al. Effectively unlocking the potential molecular room temperature phosphorescence of pure carbazole derivatives. *Adv. Opt. Mater.* **10**, 2200090 (2022).
322. Zhang, J. et al. Highly efficient and robust full-color organic afterglow through 2D superlattices embedment. *Adv. Mater.* **34**, e2206712 (2022).
323. Zhang, X. et al. Irreversible humidity-responsive phosphorescence materials from cellulose for advanced anti-counterfeiting and environmental monitoring. *ACS Appl. Mater. Interfaces.* **14**, 16582-16591 (2022).
324. Zhang, X. et al. Highly efficient and persistent room temperature phosphorescence from cluster exciton enables ultrasensitive off-on VOC sensing. *Matter.* **5**, 3499-3512 (2022).
325. Zhang, Y. et al. Cross-linked polyphosphazene nanospheres boosting long-lived organic room-temperature phosphorescence. *J. Am. Chem. Soc.* **144**, 6107-6117 (2022).
326. Zhang, Y. et al. Photo-controlled reversible multicolor room-temperature phosphorescent solid supramolecular pseudopolyrotaxane. *Adv. Opt. Mater.* **10**, 2102169 (2022).
327. Zhou, Y. et al. Dual promotion of phosphorus groups for ultralong room temperature phosphorescence with high efficiency. *Adv. Opt. Mater.* **11**, 2201904 (2022).
328. Zhu, T., Yang, T., Zhang, Q. and Yuan, W. Z. Clustering and halogen effects enabled red/near-infrared room temperature phosphorescence from aliphatic cyclic imides. *Nat. Commun.* **13**, 2658 (2022).
329. Chen, B., Huang, W. and Zhang, G. Observation of chiral-selective room-temperature phosphorescence enhancement via chirality-dependent energy transfer. *Nat. Commun.* **14**, 1514 (2023).
330. Chen, H. et al. Modulating triplet excited states of organic semiconductors via tuning molecular conformation for dual-ratiometric thermometers. *Angew. Chem. Int. Ed.* **62**, e202302629 (2023).
331. Gao, M. et al. The effect of molecular conformations and simulated "self-doping" in phenothiazine derivatives on room-temperature phosphorescence. *Angew. Chem. Int. Ed.* **62**, e202214908 (2023).
332. Ishi-I, T., Kichise, R., Park, I. S., Yasuda, T. and Matsumoto, T. Room temperature phosphorescence in longer-wavelength red light region found in benzothiadiazole-based dyes. *J. Mater. Chem. C.* **11**, 3003-3009 (2023).
333. Jiang, D. et al. Modulating room temperature phosphorescence through intermolecular halogen bonding. *J. Mater. Chem. C.* **11**, 4203-4209 (2023).
334. Kong, L. et al. Color-tunable and ultralong organic room temperature phosphorescence from poly(acrylic acid)-based materials through hydrogen bond engineering. *J. Mater. Chem. C.* **11**, 1960-1970 (2023).
335. Li, J. A. et al. Switchable and highly robust ultralong room-temperature phosphorescence from polymer-based transparent films with three-dimensional covalent networks for erasable light printing. *Angew. Chem. Int. Ed.* **62**, e202217284 (2023).
336. Li, S. et al. Macrocyclization-induced phosphorescence enhancement of pyridinium-based macrocycles. *J. Mater.*

Chem. A. **11**, 4957-4962 (2023).

337. Liang, Y. et al. Color-tunable dual-mode organic afterglow from classical aggregation-caused quenching compounds for white-light-manipulated anti-counterfeiting. *Angew. Chem. Int. Ed.* **62**, e202217616 (2023).

338. Marin-Beloqui, J. M. et al. Generating long-lived triplet excited states in narrow bandgap conjugated polymers. *J. Am. Chem. Soc.* **145**, 3507-3514 (2023).

339. Niu, Y. et al. A universal strategy for achieving dual cross-linked networks to obtain ultralong polymeric room temperature phosphorescence. *Sci. China Chem.* **66**, 1161-1168 (2023).

340. Song, X. et al. Phosphine-manipulated p-π and π-π synergy enables efficient ultralong organic room-temperature phosphorescence. *Angew. Chem. Int. Ed.* **62**, e202300980 (2023).

341. Wang, T., Gupta, A. K., Wu, S., Slawin, A. M. Z. and Zysman-Colman, E. Conjugation-modulated excitonic coupling brightens multiple triplet excited states. *J. Am. Chem. Soc.* **145**, 1945-1954 (2023).

342. Wang, X. et al. A high-contrast polymorphic difluoroboron luminogen with efficient RTP and TADF emissions. *Chem. Commun.* **59**, 1377-1380 (2023).

343. Wei, J. et al. Conformation-dependent dynamic organic phosphorescence through thermal energy driven molecular rotations. *Nat. Commun.* **14**, 627 (2023).

344. Wu, X., Peng, X., Chen, L., Tang, B. Z. and Zhao, Z. Through-space conjugated molecule with dual delayed fluorescence and room-temperature phosphorescence for high-performance OLEDs. *ACS Mater. Lett.* **5**, 664-672 (2023).

345. Xia, Y. et al. Host-guest doping in flexible organic crystals for room-temperature phosphorescence. *Angew. Chem. Int. Ed.* **62**, e202217547 (2023).

346. Xiao, H., Zheng, D. S., Zhang, L. Y., Xu, L. J. and Chen, Z. N. Ultra-long room temperature phosphorescence with the efficiency over 64% induced by 1‰ impurity doping. *Adv. Funct. Mater.* **33**, 2214241 (2023).

347. Xu, P., Hojo, R. and Hudson, Z. M. Thermally activated delayed fluorescence and room-temperature phosphorescence in materials with imidazo-pyrazine-5,6-dicarbonitrile acceptors. *Chemistry.* **29**, e202203585 (2023).

348. Yang, Z. et al. Pressure-induced room-temperature phosphorescence enhancement based on purely organic molecules with a folded geometry. *Chem. Sci.* **14**, 2640-2645 (2023).

349. Ye, W. et al. Respiration-responsive colorful room-temperature phosphorescent materials and assembly-induced phosphorescence enhancement strategies. *Small.* **19**, 2207403 (2023).

350. Zhang, X. et al. A class of organic units featuring matrix-controlled color-tunable ultralong organic room temperature phosphorescence. *Adv. Sci.* **10**, e2206482 (2023).

351. Zhang, X. et al. Cellulose-based ultralong room-temperature phosphorescence nanomaterials with tunable color and high quantum yield via nano-surface confining effect. *Research.* **6**, 0029 (2023).

352. Lu, T. & Chen, F. Multiwfn: A multifunctional wavefunction analyzer. *J. Comput. Chem.* **33**, 580-592 (2012).

353. Johnson, E. et al. Revealing noncovalent interactions. *J. Am. Chem. Soc.* **132**, 6498-6506 (2010).

354. Frisch, M. et al. Gaussian 09 revison C.01; Gaussian, Inc.: Wallingford, CT, 2009.
