# Supplementary material for: Abnormal thermally-stimulated dynamic organic phosphorescence
Source: Nat Commun. 2024 Mar 8;15:2134. doi: 10.1038/s41467-024-45811-0 (PMC10923930; doi:10.1038/s41467-024-45811-0)
Supplement: Supplementary file 2 — Description of Additional Supplementary Files [file 41467_2024_45811_MOESM2_ESM.pdf]

Description of Additional Supplementary Files

File Name: Supplementary Movie 1
Description: Temperature dependent colorful phosphorescence of FPO crystals. As temperature increases from 293 to 343 K, the afterglow color was tuned from yellow to cyan-blue.

File Name: Supplementary Movie 2
Description: Colourful afterglow display. Under the guidance of microcontroller, various patterns were displayed in succession. Notably, in addition to microcontroller control, the patterns also show dynamic changes at different times.
